# Reappraisal of the Eocene whiptail stingrays (Myliobatiformes, Dasyatidae) of the Bolca Lagerstätte, Italy

**DOI:** 10.1111/zsc.12330

**Published:** 2018-11-28

**Authors:** Giuseppe Marramà, Giorgio Carnevale, Gavin J. P. Naylor, Jürgen Kriwet

**Affiliations:** ^1^ Department of Palaeontology University of Vienna Vienna Austria; ^2^ Dipartimento di Scienze della Terra Università degli Studi di Torino Torino Italy; ^3^ Florida Museum of Natural History University of Florida Gainesville Florida

**Keywords:** Monte Bolca, myliobatiformes, Neotrygoninae, phylogeny, Tethys, *Tethytrygon* gen. n.

## Abstract

The Eocene whiptail stingrays of the family Dasyatidae from the Bolca Lagerstätte, NE Italy, are revised herein in detail. The analysis of the anatomical and morphometric features allows us to identify the species “*Dasyatis*”* zigni* (Molin, 1861) as a junior synonym of “*D.*”* muricatus *(Volta, 1796), and to assign it to the new genus *Tethytrygon *gen. n. This new taxon exhibits a unique combination of features (e.g., rhombic disc wider than long, elongated tail folds fail to reach the tip of the tail, thorns absent, single serrated tail sting, “caniniform” teeth on upper jaw, tooth crown ornamentation absent, 175–179 vertebrae, 108–117 pectoral radials, 24–27 pelvic radials and other features of clasper anatomy) that clearly support its attribution to the subfamily Neotrygoninae of the stingray family Dasyatidae. The morphological and phylogenetic affinities of *Tethytrygon* gen. n. with the living neotrygonines (*Neotrygon* and *Taeniura*) suggest a close association of this taxon with the tropical shallow‐water habitats hypothesized for the Bolca palaeoenvironment during the early Eocene. Moreover, the analysis of the fossil occurrences of the neotrygonines provides new insights into the role of the Tethys for the origin and evolutionary history of certain whiptail stingrays.

## 1 INTRODUCTION

The family Dasyatidae, whose representatives are known as whiptail stingrays, includes about 86 living species in 19 genera of small to large stingrays (up to 2.6 m disc width, weighing up to 600 kg) living in demersal inshore habitats of continental and insular shelves up to a depth of 600 m (Last, Naylor, & Manjaji‐Matsumoto, 2016; Last, White, Carvalho, et al., 2016). Morphological characters traditionally used to distinguish whiptail stingrays from other myliobatiformes include a variably depressed circular to rhombic disc not more than 1.3 times as broad as long, an angular to obtuse and sometimes very elongated snout, absent caudal and dorsal fins, greatly elongated and slender to whip‐like tail, one to four long venomous spines, and a skin ranging from being completely smooth to covered with dermal denticles and thorns (Cappetta, 2012; Last, Naylor et al., 2016; Last, White, Carvalho, et al., 2016; Nelson, Grande, & Wilson, 2016). Although some previous studies suggest that the Dasyatidae might be a non‐monophyletic group (e.g., Aschliman, Claeson, & McEachran, 2012; Carvalho, Maisey, & Grande, 2004; Lovejoy, 1996), recent morphological and molecular analyses proposed that this family should be actually regarded as monophyletic (e.g., Aschliman, Nishida, et al., 2012; Bertozzi, Lee, & Donnellan, 2016; Marramà, Klug, Vos, & Kriwet, 2018; Naylor et al., 2012) consisting of four major subgroups at subfamilial level: the Dasyatidae, Hypolophinae, Urogymninae and Neotrygoninae (Last, Naylor et al., 2016). The latter subfamily was created by Last, Naylor, et al. (2016) and comprises species of *Neotrygon* and *Taeniura*, two genera today restricted to the Indian Ocean and Indo‐Australian Archipelago, which are unique among dasyatids in having a series of enlarged caniniform teeth on the upper jaw in both sexes and a short tail that is <1.7 times the disc width (Cappetta, 2012; Last, Naylor et al., 2016; Last, White, Carvalho, et al., 2016).

Although the fossil record of stingrays is well represented and dates back to the Early Cretaceous, it is heavily biased towards isolated teeth, dermal denticles and caudal spines (Cappetta, 2012; Underwood, Mitchell, & Veltcamp, 1999). Nearly complete and articulated fossil stingrays have been only recovered from Palaeogene marine sediments of the Bolca Lagerstätte in Italy (Marramà, Carnevale, Engelbrecht, et al., 2018) and Grube Unterfeld in Germany (Hovestadt, Hovestadt‐Euler, & Micklich, 2010), from Eocene freshwater deposits of the Green River Formation, USA (Carvalho et al., 2004), and from Miocene marine deposits of Indonesia (Marramà et al., 2018). The celebrated Eocene (Ypresian, ca. 49 Ma; Papazzoni, Carnevale, Fornaciari, Giusberti, & Trevisani, 2014) Bolca Konservat‐Lagerstätte from north‐eastern Italy is mainly known for the outstanding diversity and preservational quality of bony fish species that provide evidence of the recovery of shallow marine settings associated with reefs after the K‐Pg boundary (Carnevale, Bannikov, Marramà, Tyler, & Zorzin, 2014; Friedman & Carnevale, 2018; Marramà, Garbelli, & Carnevale, 2016b). However, Bolca is also one of the few fossiliferous sites in which fossils of chondrichthyan fishes are exquisitely preserved and represented by nearly complete and articulated skeletons. Recent studies have provided a new perspective about the chondrichthyan palaeobiodiversity of this deposit, which includes possibly more than a dozen of species‐level taxa belonging to a variety of holocephalan, selachian and batoid lineages, including chimaeriformes, carcharhiniformes, lamniformes, torpediniformes, rhinopristiforms and myliobatiformes (Fanti, Minelli, Larocca Conte, & Miyashita, 2016; Marramà et al., 2018; Marramà, Carnevale, & Kriwet, 2018; Marramà, Claeson, Carnevale, & Kriwet, 2018; Marramà, Engelbrecht, Carnevale, & Kriwet, 2017). However, since the comprehensive account of cartilaginous fishes from Bolca written by Jaekel (1894), no modern systematic studies have been carried out on the Bolca stingrays. The goal of this paper is therefore to redescribe the anatomy of the two species from Bolca included in the family Dasyatidae (“*Dasyatis*”* muricatus* and “*D*.” *zigni*) in detail, to review their taxonomic status and to discuss their relationships within the Myliobatiformes.

## 2 MATERIALS AND METHODS

The present study is based on 13 nearly complete and articulated specimens, which are currently housed in the Museo Civico di Storia Naturale di Verona (MCSNV), Museo dei Fossili di Bolca (technically part of the MCSNV), registered private collection of Cerato Massimo Cipriano (CMC), Museo di Geologia e Paleontologia dell'Università degli Studi di Padova (MGP‐PD), Museum National d'Histoire Naturelle, Paris (MNHN), Museo Geologico Giovanni Capellini, Università degli Studi di Bologna (MGGP), Carnegie Museum, Pittsburgh (CMNH), and Museum of Comparative Zoology, Harvard University (MCZ). The studied material includes both the historical specimens and new specimens collected from excavations carried out in the second half of 20th century. Some of the specimens were examined under ultraviolet light in order to distinguish the preserved soft tissues from grout or pigments used in historical reconstruction. Measurements were taken to the nearest 0.1 mm, and body proportions are detected based on disc width (DW) following Last, Naylor, & Manjaji‐Matsumoto (2016); Last, White, Carvalho, et al. (2016); Last, White, and Naylor (2016). Osteological and tooth terminology primarily follow Herman, Hovestadt‐Euler, Hovestadt, and Stehmann (1998), Herman, Hovestadt‐Euler, Hovestadt, and Stehmann (1999), Herman, Hovestadt‐Euler, Hovestadt, and Stehmann (2000), Lovejoy (1996), Nishida (1990), and Carvalho et al. (2004). Morphometric terminology is adopted and modified from Last, Naylor, & Manjaji‐Matsumoto (2016); Last, White, Carvalho, et al. (2016); Last, White, and Naylor (2016). Biometric analyses (Supporting information Appendix S1), performed to test the homogeneity of the sample and to confirm its assignment to a single species, follow those from Marramà and Carnevale (2015), Marramà, Lombardo, Tintori, and Carnevale (2017) and Cawley, Marramà, Carnevale, and Kriwet (2018).

The phylogenetic analysis is based on the morphological data set of Marramà et al. (2018), which in turn is based on the matrices of Carvalho et al. (2004) and Claeson et al. (2010), and supplemented with characters from Herman et al. (1998), Herman et al. (1999), Herman et al. (2000), Schaefer and Summers (2005), Aschliman, Claeson, et al. (2012), Lim, Lim, Chong, and Loh (2015), Last, White, Carvalho, et al. (2016); Last, Naylor, & Manjaji‐Matsumoto (2016) and Underwood, Kolmann, and Ward (2017) (Supporting information Appendix S1). The matrix was compiled in mesquite v.3.03 (Maddison & Maddison, 2008), and the phylogenetic analysis was performed with tnt v.1.5 using the branch‐and‐bound method (Goloboff, Farris, & Nixon, 2008). All characters are unordered and given equal weight. Tree length, consistency and retention indices, and Bremer support were subsequently calculated for the single parsimonious tree retrieved.

### 2.1 Extant comparative material examined

*Taeniura lymma*, IUWP (Department of Palaeontology of the University of Vienna) uncatalogued specimen; *Neotrygon* sp. IPUW 7355: these two specimens were cleared and stained at the Department of Palaeontology of the University of Vienna following the procedure used by Walker & Kimmel (2007) and investigated non‐invasively with a micro‐CT device SkyScan/Bruker 1173 at the Department of Palaeontology of University of Vienna. The processing of the tiff‐image stacks was performed with amira v.5.4.1 (Visualization Sciences Group); *Neotrygon kuhlii*, BMNH (Natural History Museum of London) 2015.1.25.6 (CT scan renders provided by C. Underwood); *Potamotrygon tigrina*, IUWP 7361; *Rhinoptera* sp., IUWP uncatalogued, dissected specimen.

## 3 REASSESMENT OF THE WHIPTAIL STINGRAYS OF BOLCA

**Figure 1:**
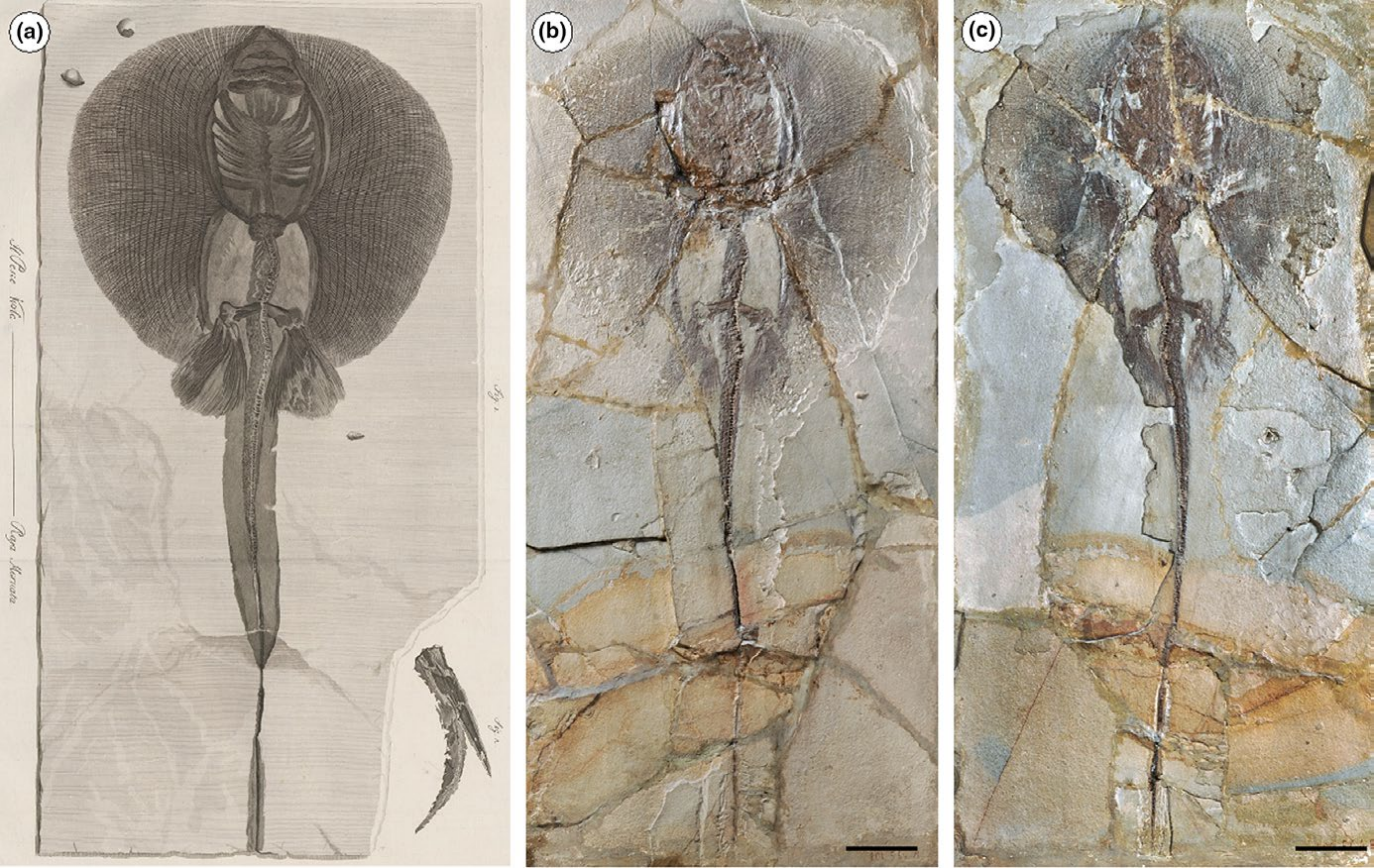
(a‐c) *Tethytrygon muricatus* (Volta, 1796) from the Eocene of Bolca Lagerstätte. (a) Historical plate of the holotype of *T. muricatus* MNHN F.Bol.564 illustrated and specified as *Raja muricata* in Volta (1796, pl. 9); photo: courtesy of Roberto Zorzin and Museo Civico di Storia Naturale di Verona. (b and c) The holotype MNHN F.Bol.564 in part and counterpart. Scale bars 50 mm [Colour figure can be viewed at wileyonlinelibrary.com]

**Figure 2:**
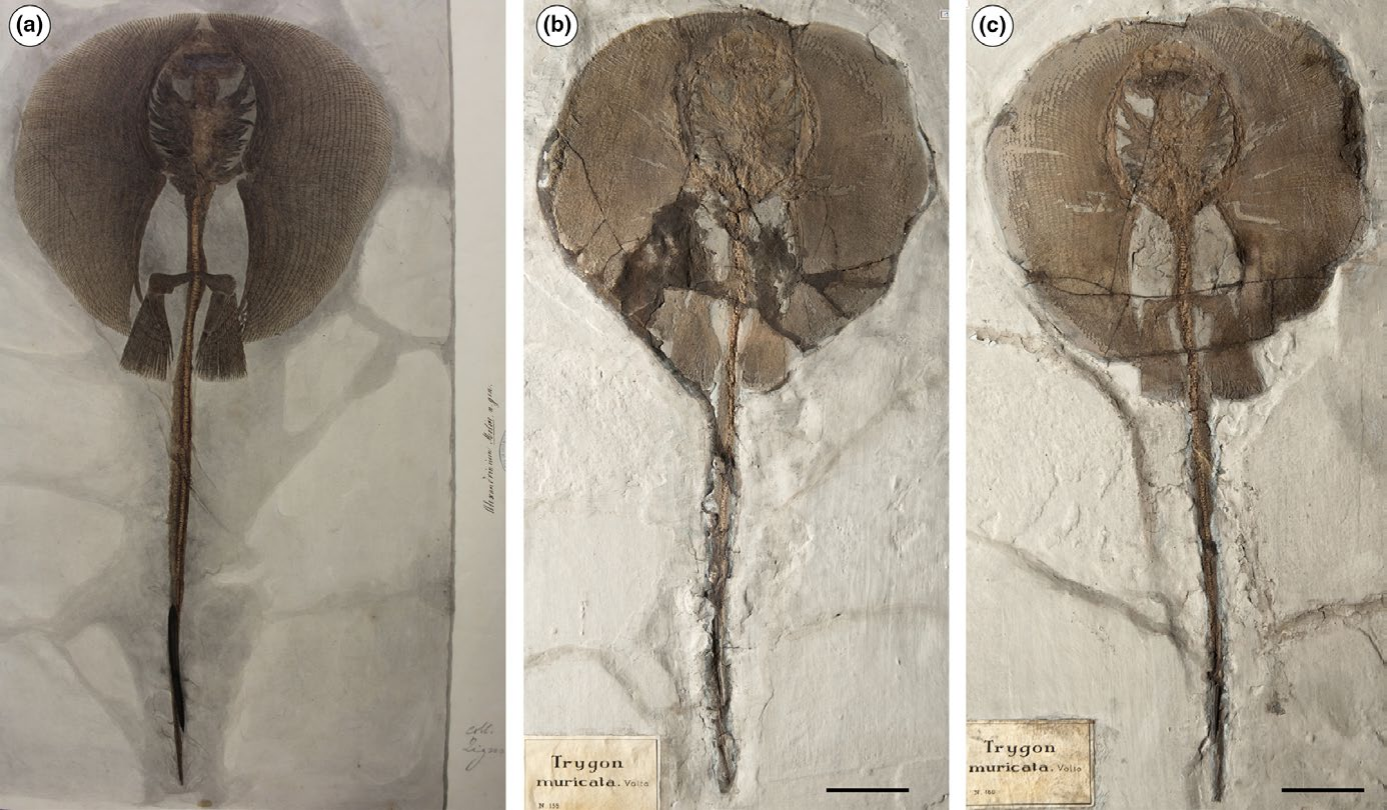
(a‐c) *Tethytrygon muricatus *(Volta, 1796) from the Eocene of Bolca Lagerstätte. (a) Unpublished plate of the specimen MGP‐PD 159Z/160Z illustrated and specified as *Alexandrinum molinii *by Achille de Zigno (1813–1892); photo: courtesy of Università degli Studi di Padova. (b and c) Part and counterpart of the specimen MGP‐PD 159Z/160Z. Scale bars 50 mm [Colour figure can be viewed at wileyonlinelibrary.com]

Whiptail stingrays of the family Dasyatidae are among the first cartilaginous fishes described from the Bolca Lagerstätte, but their taxonomic history is characterized by an intricate and complex scenario. The first taxon was described and figured by Volta (1796, pl. 9, fig. 1) under the name *Raja muricata* based on a single specimen from the Gazola collection in part and counterpart, today housed in the Museum National d'Histoire Naturelle in Paris, registered under MNHN F.Bol.564 (Figure 1). On the same plate, Volta (1796, pl. 9, fig. 2) figured an incomplete portion of a tail preserving a caudal sting using the same name that was later assigned to *Taeniura knerii* by Molin (1861), a taxon considered a synonym of *Urolophus crassicaudatus* by Eastman (1904, 1905a, 1905b). Later, de Blainville (1818) assigned MNHN F.Bol.564 to *Trygonobatus vulgaris*, and Agassiz (1835), Agassiz (1833–1844) to *Trygon gazzolae*, without any reliable new description or anatomical interpretation. Subsequently, based on specimen MGP‐PD 159Z/160Z, Molin (1861) created the genus *Alexandrinum*, which was specified as *Alexandrinum molini *by de Zigno (1874a) and figured in unpublished material (Figure 2). All these taxa and their respective specimens were referred to *Trygon* by Jaekel (1894), with *R. muricata* being considered as the holotype (*Trygon* Cuvier, 1816 is today regarded as a junior synonym of *Dasyatis *Rafinesque, 1810). The synonymy of *Trygon muricatus* was subsequently confirmed by Eastman (1904, 1905a, 1905b, 1911).

**Figure 3:**
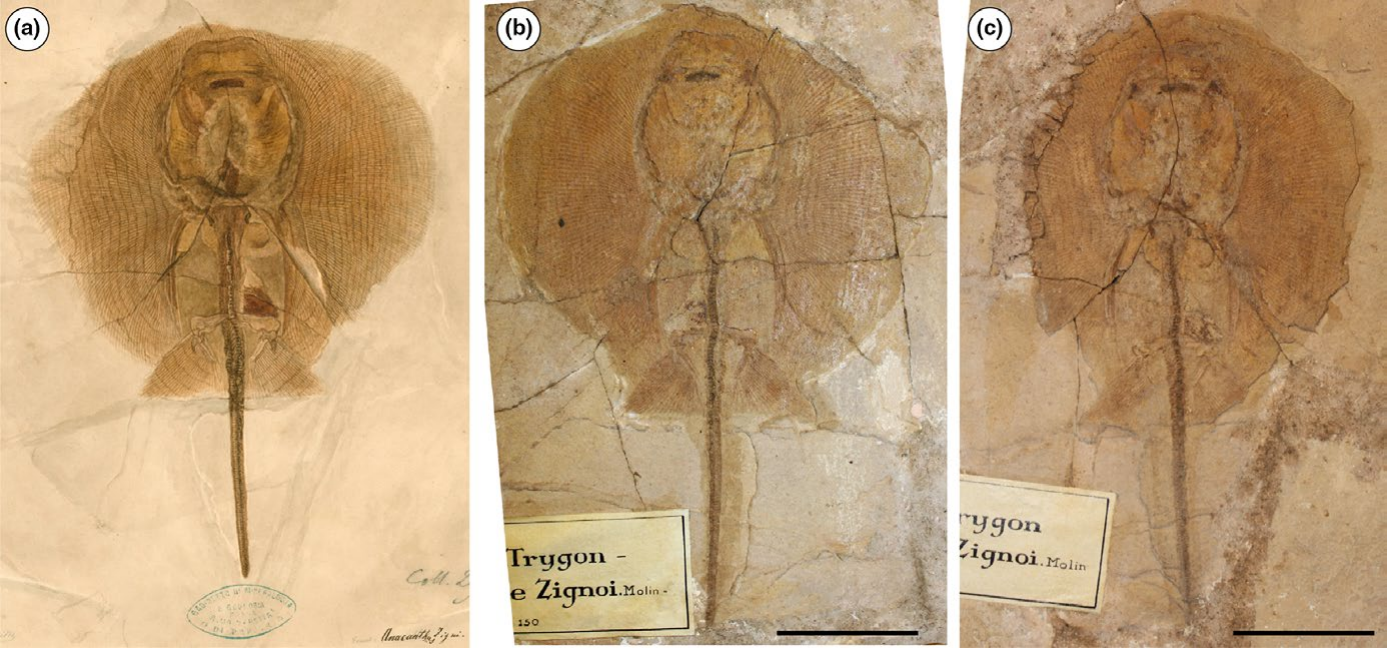
(a‐c) *Tethytrygon muricatus* (Volta, 1796) from the Eocene of Bolca Lagerstätte. (a) Unpublished plate of the specimen MGP‐PD 150Z/151Z illustrated and specified as *Anacanthus zignii *by Achille de Zigno (1813–1892); photo: courtesy of Università degli Studi di Padova. (b and c) Part and counterpart of the specimen MGP‐PD 150Z/151Z. Scale bars 50 mm [Colour figure can be viewed at wileyonlinelibrary.com]

**Figure 4:**
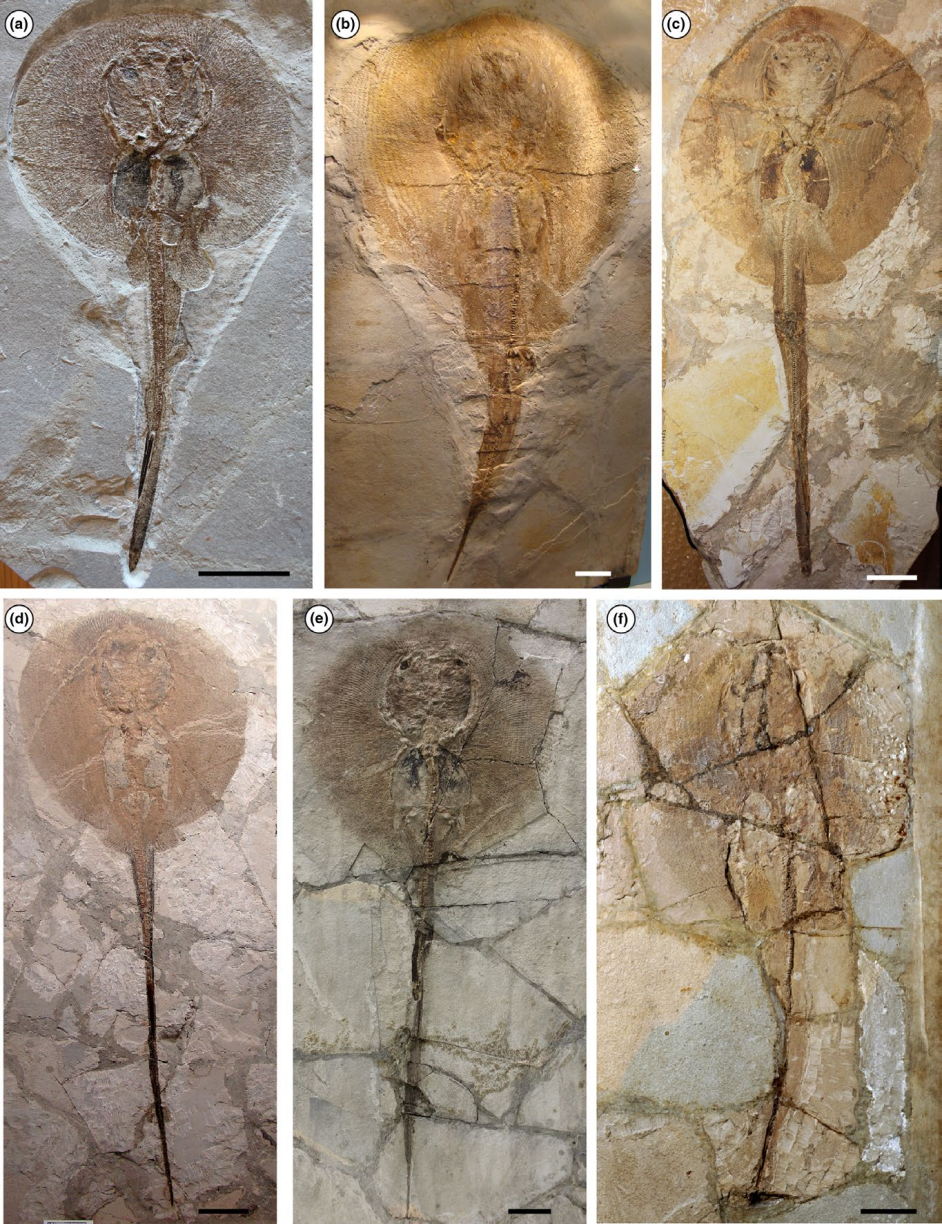
(a‐f) Selected specimens of *Tethytrygon muricatus* (Volta, 1796) from the Eocene of Bolca Lagerstätte. (a) CMC2, juvenile female individual. (b) MCSNV IG.23194, adult male. (c) MCSNV IG.186653, adult female. (d) MCSNV T.1021, subadult female. (e) MCSNV II.B.92, subadult female. (f) MNHN F.Bol568, adult female. Scale bars 50 mm [Colour figure can be viewed at wileyonlinelibrary.com]

**Figure 5:**
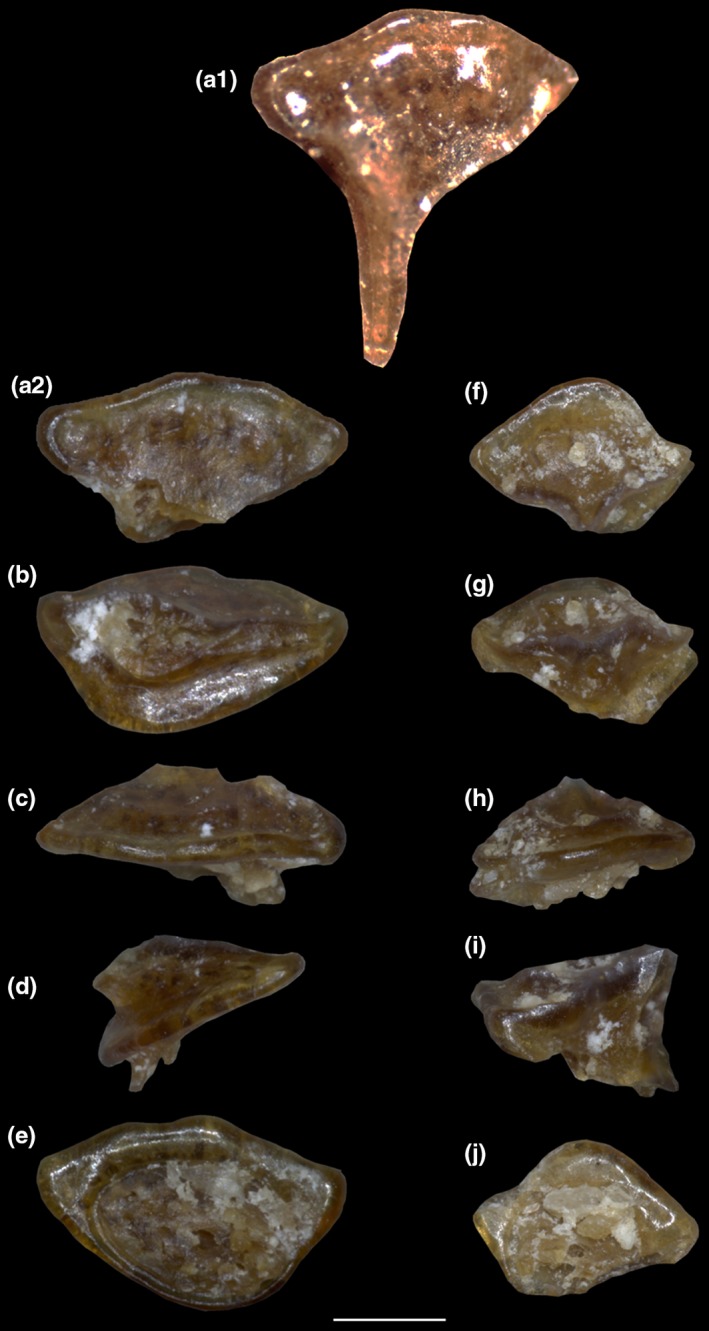
(a‐j) *Tethytrygon muricatus* (Volta, 1796) from the Eocene of Bolca Lagerstätte (specimen MCSNV IG.186653) . (a‐e) A single tooth from the file of “caniniform” teeth in (a) occlusal, (b) lingual, (c) labial, (d) lateral and (e) basal view; the picture depicted in A1 represent the same tooth still in place (the cusp has broken during the extraction). (f‐j) Another isolated tooth (not coming from the file of “caniniform” teeth) in (f) occlusal, (g) lingual, (h) labial, (i) lateral and (j) basal view. Scale bar is 500 μm [Colour figure can be viewed at wileyonlinelibrary.com]

Based on another single incomplete specimen lacking part of the tail and the sting (MGP‐PD 150Z/151Z), Molin (1861) described another dasyatid taxon as *Anacanthus zigni*. As reported later by de Zigno (1874a, 1874b), who also figured the specimen in unpublished material (Figure 3), Molin diagnosed and distinguished the new taxon from *R. muricata* based on the supposed absence of a caudal sting and a tail that is shorter than the disc length. Subsequently, Jaekel (1894) recognized that the presence of a very short tail and the absence of a sting in MGP‐PD 150Z/151Z were due to the incompleteness of the specimen, and assigned the species to the genus *Trygon*. However, he maintained and distinguished *Trygon zigni* (Molin, 1861) from *T. muricatus* (Volta, 1796) based on the “much smaller size […] a less rounded outline of the disc […] and pelvic fins triangular in shape.” Since then, no detailed anatomical descriptions and taxonomic interpretation of the whiptail stingrays of Bolca were carried out. Our revision of the material showed that no substantial morphological differences support the hypothesis that MGP‐PD 150Z/151Z (nor MGP‐PD 159Z/160Z) should be recognized as a different species. The analysis of the anatomical and morphometric features allow us to recognize the species “*Dasyatis*”* zigni* (Molin, 1861) as a junior synonym of “*Dasyatis*”* muricatus *(Volta, 1796) and to assign it to the new genus *Tethytrygon *gen. n. The new taxon is represented by 13 partially complete and articulated skeletons (Figures 1, 2, 3, 4). The large number of available specimens and their good preservation allowed for the recognition and description of several skeletal and dental characters, which are useful to distinguish and separate the taxon from any other known living and fossil dasyatid (see the detailed anatomical description in the Supporting information Appendix S1). The specimens examined comprise different ontogenetic stages, with the largest one (an adult male) being characterized by 60 cm disc width and possibly reaching 150 cm in total length. The disc of *Tethytrygon* gen. n. is rhombic, not wing‐like, reaching the maximum width in the anterior third of disc length. The disc length is slightly shorter than the disc width (0.9 times), whereas the total length is about 2.6 and 2.8 times those of the disc width and disc length, respectively. The tail is long and about 1.8 times the disc width. *Tethytrygon muricatus* lacks dorsal fins, whereas a single serrated sting can be recognized in most of the specimens. The placement within the subfamily Neotrygoninae is particularly supported by the presence of files of “caniniform” teeth in the upper jaw (Figure 5), which represent a unique and derived trait for *Neotrygon* and *Taeniura* among stingrays (Cappetta, 2012; Last, Naylor, & Manjaji‐Matsumoto, 2016; Last, White, Carvalho, et al., 2016) and supports the grouping of *Tethytrygon* gen. n. with these genera in our phylogeny. *Tethytrygon muricatus* is a unique neotrygonine in having the following autapomorphic traits: large size (up to 60 cm DW and possibly 150 cm TL), long tail (170.4%–184.7% DW) and low number of monospondylous trunk vertebrae (23–26). Additionally, *Tethytrygon* gen. nov is also characterized by a unique combination of morphological and meristic characters that allow to distinguish it from the other neotrygonines (Table 1). These features include a disc rhombic in shape, disc length 87.2%–95.2% DW, total length 249.5%–263.0% DW, subtriangular pelvic fins 24.6%–29.9% DW and eye diameter 2.7%–4.5% DW. The skin of *T. muricatus* is mostly smooth without thorns but with small scattered star‐shaped dermal denticles in largest individuals. A single serrated sting of 26.1%–32.7% DW can be recognized in most of the specimens. The vertebral column is composed of 175–179 vertebrae. The pectoral disc contains 108–117 pectoral radials of which 49–53 are propterygial, 16–20 are mesopterygial, and 40–45 are metapterygial radials. The pelvic fins have 24–26 radials. The tail folds are located posterior to caudal sting origin and fail to reach the tip of the tail as in *Neotrygon*. The teeth are rhombic in occlusal view and possess well‐marked concavely arched cutting edges. Their lingual surface is low and strongly concave and the crown ornamentation is absent.

**Table 1:** Morphological and meristic characters useful to distinguish *Tethytrygon *gen. n. from the living neotrygonines *Taeniura* and *Neotrygon*. All measurements as percentage of disc width (%DW) and mean values are within parentheses. The living species include *Neotrygon annotata, N. australiae, N. caeruleopunctata, N. indica, N. kuhli*, *N. leylandi, N. ningalooensis*, *N. orientalis, N. picta*, *N. trigonoides*, *N. varidens*, *Taeniura lymma *and *T. lessoni*. Data from Schwartz (2005), Schwartz, (2007), Schwartz (2008), Last and White (2008), Last, White, Carvalho, et al. (2016), Last, Naylor, et al. (2016), Last, White, and Naylor (2016) and Pavan‐Kumar, Kumar, Pitale, Shen, and Borsa (2018)

Morphometric character	*Tethytrygon*	*Taeniura*	*Neotrygon*
Max disc width (cm)	60.3	37.0	47.0
Max total length (cm)	≈150	75.0	70.0
Clasper length	19.0 (19.0)	21.2	20.0–23.3
Disc length	88.2–95.3 (92.3)	110.5–120.4	79.2–87.3
Snout to pectoral‐fin insertion	77.2–85.8 (81.9)	92.6–106.1	68.1–77.2
Orbit to pectoral‐fin insertion	61.5–68.7 (64.7)	63.4–74.1	44.5–55.9
Snout (preorbital) length	15.1–18.5 (16.9)	21.5–25.7	13.3–18.5
Pectoral‐fin insertion to sting	73.9–80.1 (79.1)	71.9–88.3	32.6–44.5
Eye diameter	2.7–4.5 (3.7)	6.6–8.4	5.1–6.6
Inter‐eye width	10.7–17.7 (14.6)	17.3–20.0	12.7–18.8
Snout to max disc width	36.3–43.5 (40.1)	51.7–57.8	36.8–41.5
Pelvic fin length	24.5–30.0 (27.6)	28.8–34.3	13.6–22.4
Pelvic girdle width	21.3–23.5 (22.7)	17.6–24.1	13.7–22.1
Preoral length	13.4–16.1 (15.0)	17.7–20.7	15.9–18.8
Prescapular distance (head length)	42.9–50.0 (45.8)	49.8–57.1	38.2–42.3
Sting length	25.3–32.8 (29.7)	20.4–29.1	13.4–19.2
Tail length	170.4–184.7 (178.2)	150.0–170.0	110.0–150.0
Total length	249.5–263.0 (255.0)	232.5–265.6	159.1–224.6
Mouth–scapulocoracoid distance	28.1–31.5 (30.0)	?	?
Neurocranial length	22.5–24.9 (23.9)	?	?
Neurocranial width	14.7–18.3 (16.2)	?	?
Pelvics to tip of tail length	141.8–156.7 (149.0)	?	?
Prepelvic distance	71.7–78.2 (75.4)	?	?
Presting length	152.6–173.4 (162.3)	?	?
Scapulocoracoid width	19.7–22.8 (21.1)	?	?
Meristic and body characters	*Tethytrygon*	*Taeniura*	*Neotrygon*
Propterygial radials	49–53 (51)	47–50	40–51
Mesopterygial radials	16–20 (18)	15–18	12–17
Metapterygial radials	40–45 (43)	47–50	44–50
Total pectoral radials	108–117 (112)	110–115	101–113
Pelvic radials	24–27 (25)	18–25	19–24
Monospondylous trunk vertebrae (excl. synarcual)	23–26 (24)	37–39	34–46
Diplospondylous vertebrae (anterior to sting)	100–109 (105)	90–101	57–67
Diplospondylous vertebrae (posterior to sting)	45–54 (48)	40–55	14–40
Total vertebrae	175–179 (177)	175–184	109–145
Number of stings	1 (1)	1–2	1–2
Sting serrations (total)	48–90 (69)	59–69	?
Tooth ornamentation	Absent	Present	Absent
Denticles	Absent/present	Absent/present	Absent/present
Thorns	Absent	Present	Absent/present

## 4 PHYLOGENETIC ANALYSIS

**Figure 6:**
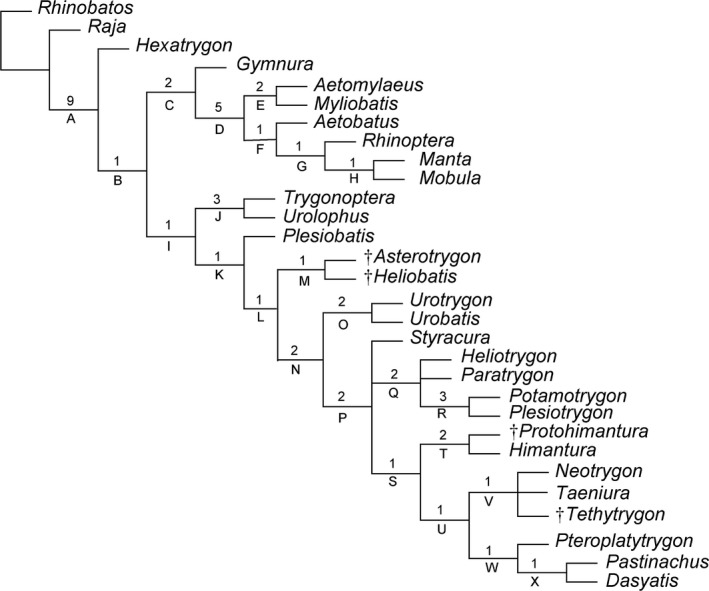
The single parsimonious tree retrieved in tnt v.1.5 based on 103 morphological characters showing the hypothetical relationships of *Tethytrygon muricatus* (Volta, 1796) within the Myliobatiformes. Numbers on nodes indicate the Bremer support. Extinct taxa are marked with a dagger. The list of synapomorphies on each node (capital letters) is given in Table 2

Carvalho et al. (2004, fig. 51) tentatively placed *T. muricatus *(as “*Dasyatis*”* muricata*) within the Myliobatiformes. Though they recognized its affinities with dasyatids, they placed it conservatively in an unsolved polytomy together with *Dasyatis*, *Himantura*, *Styracura* (as “*Himantura*”), *Pteroplatytrygon* and *Taeniura*. Our analysis of 103 traits coded for 30 taxa produced a single parsimonious tree (length 216 steps, C.I 0.65, R.I 0.79) that resolved many of the systematic affinities of *T. muricatus* (Figure 6). A complete list of synapomorphies at each node is listed in Table 2. The tree is similar to the one depicted in by Marramà et al. (2018, fig. 8b) and only differs in the improved resolution of the positions of *Plesiobatis* as well as of the Eocene freshwater stingrays *Asterotrygon* and *Heliobatis*. The monophyly of the Myliobatiformes, as recognized by McEachran, Dunn, and Miyake (1996), Carvalho et al. (2004), McEachran and Aschliman (2004), Aschliman, Claeson, et al. (2012) and Marramà et al. (2018), is confirmed and strongly supported herein (Bremer value 9) by 10 synapomorphies: basihyal as a single element, but separate from first hypobranchials (character 19[1]); presence of a median projection of the basibranchial medial plate (ch. 22[1]); presence of levator and depressor rostri muscles (ch. 66[1]), serrated tail stings (ch. 67[1]); thorns absent (ch. 69[1]); rostral cartilage vestigial or absent (ch. 73[1]); postorbital process very broad and shelf‐like (ch. 74[1]); jugal arch absent (ch. 75[1]); presence of ball and socket articulation between scapular process and synarcual (ch. 78[1]); presence of a thoracolumbar synarcual (ch. 79[1]).

**Table 2:** List of synapomorphies for each node depicted in Figure 6. See the explanation of characters and states in Supporting information Appendix S1

Node	Clade	Synapomorphies
A	Myliobatiformes	19(1), 22(1), 66(1), 67(1), 69(1), 73(1), 74(1), 75(1), 78(1), 79(1),
B	–	12(1), 21(1), 43(1)
C	Myliobatoidea	10(1), 27(1), 28(1), 34(2), 81(3), 100(0), 101(1)
D	Myliobatidae	7(1), 11(1), 15(1), 17(1), 18(1), 19(3), 21(2), 22(0), 23(1), 25(1), 33(1), 35(1), 37(1), 38(1), 44(1), 45(1), 46(1), 48(1), 54(1), 57(1), 60(1), 61(1), 70(3), 71(2), 76(3), 96(1), 97(1), 98(1)
E	–	55(1), 92(2)
F	–	9(1), 24(1), 27(2), 51(1)
G	–	5(1), 6(1), 28(0)
H	–	44(0), 95(1)
I	Dasyatoidea	88(1)
J	Urolophidae	8(1), 29(2), 99(1)
K	–	68(1)
L	–	69(0)
M	Heliobatidae	34(1)
N	–	19(2), 25(1), 76(1)
O	Urotrygonidae	1(1), 41(1)
P	–	33(1), 34(2), 81(2), 85(1), 88(2)
Q	Potamotrygonidae	3(2), 25(0), 30(1), 39(1), 40(1)
R	–	3(1), 14(1), 24(1), 26(1), 34(1), 36(2), 71(1)
S	Dasyatidae	87(1), 89(1)
T	Urogymninae	99(1), 102(1)
U	–	34(1), 83(0), 84(1)
V	Neotrygoninae	36(1), 92(1), 103(1)
W	–	82(1), 88(1)
X	–	57(1&2)

The absence of ribs (ch. 80), traditionally recognized as a synapomorphy of stingrays, does not support the clade in our phylogeny, since skates (here represented by *Raja*) lack ribs as well (Marramà, Schultz, & Kriwet, 2018). *Hexatrygon* (Sixgill stingray) is inferred to be the sister to all other stingrays in most analyses based on morphological data (Aschliman, Claeson, et al., 2012; Aschliman, Nishida, et al., 2012; Carvalho et al., 2004; Claeson et al., 2010; Marramà et al., 2018), but not in recent molecular phylogenies, where it was recovered nested within myliobatiformes being sister to the urolophids (Naylor, Caira, Jensen, Rosana, Straube et al., 2012; Naylor, Caira, Jensen, Rosana, White et al., 2012) or to *Gymnura* (Bertozzi et al., 2016).

Our phylogeny detected a dichotomous nature of remaining myliobatiformes as determinated by Marramà et al. (2018). The dichotomy is formed by two main clades that correspond in part to the superfamilies Myliobatoidea and Dasyatoidea. The nature of the dichotomy is possibly linked to the different calcifications of radial cartilages, body shapes and swimming modes detected in these two main groups by Schaefer and Summers (2005). The monophyly of the myliobatoids, including *Gymnura* as sister to pelagic stingrays, is supported herein by seven synapomorphies: short orbital region with more anteriorly placed supraorbital and postorbital process (ch. 10[1]); mesopterygium fragmented (ch. 27[1]); lateral expansion of radials in pectoral region (ch. 28[1]); caudal fin absent (ch. 34[2]); first segment of propterygium adjacent to anterior margin of antorbital cartilage or anterior to margin of nasal capsule (ch. 81[3]); “crustal” calcification pattern of radials (ch. 100[0]); and wing‐like body shape with pectoral fins greatly expanded (ch. 101[1]). This clade includes those stingrays with crustal calcification of radials and a wing‐like body shape that possibly reflect their unique oscillatory swimming mode (Schaefer & Summers, 2005).

The tree presents a hypothesis that contrasts with more recent analyses (e.g., Aschliman, Nishida, et al., 2012; Naylor, Caira, Jensen, Rosana, Straube et al., 2012; Naylor, Caira, Jensen, Rosana, White et al., 2012) in resurrecting the *Gymnura *+ Myliobatidae clade, whose relationship is only weakly supported according to Aschliman (2014) because of the limited set of taxa and ambiguous character states. Recent molecular analyses resolved *Gymnura* as sister to *Urolophus* (Aschliman, Nishida, et al., 2012), *Plesiobatis* (Naylor et al., 2012), *Hexatrygon* (Bertozzi et al., 2016), or placed it much closer to the base of all myliobatiformes (Last, White, Carvalho, et al., 2016). The family Myliobatidae (including *Aetomylaeus*, *Myliobatis*, *Aetobatus*, *Rhinoptera*, *Manta* and *Mobula*) is herein detected as monophyletic and well supported (Bremer value 5) by 28 characters (see Table 2). The monophyly of the clade Dasyatoidea (including all remaining stingrays) is weakly supported (Bremer value 1) by a single character, the spiracularis split into lateral and medial bundles, with the medial bundle inserting on to the posterior surface of Meckel's cartilage and the lateral bundle inserting onto the dorsal edge of the hyomandibula (ch. 88[1]). This group includes stingrays having rhomboidal or oval disc shapes and “catenated” calcification of radials, which reflect their undulatory swimming mode and benthic habits (Schaefer & Summers, 2005). The family Urolophidae (*Urolophus *+ *Trygonoptera*) is sister to all dasyatoids, and its monophyly as detected by Carvalho et al. (2004) and Bertozzi et al. (2016) is confirmed and well supported herein (Bremer value 3) by three characters: very enlarged foramen for the optic (II) nerve (ch. 8[1]); external margin of mesopterygium highly sinuous, fused with articulating radial elements (ch. 29[2]); and presence of a second transverse tooth keel (ch. 99[1]). In Marramà et al. (2018, Figure 8) the systematic position of *Plesiobatis*, *Asterotrygon* and *Heliobatis* was poorly resolved and the analysis detected two different hypotheses. Possibly due to the recoding of some characters (Supporting information Appendix S1) and to the inclusion of the new taxon described herein, our new analysis detected a single tree in which *Plesiobatis* is more basal than the Eocene freshwater stingrays *Asterotrygon* and *Heliobatis*. These two fossil taxa form a monophyletic clade supported by a single character (caudal fin reduced to tail folds; ch. 34[1]). In fact, since this character is absent in the outgroups, *Hexatrygon*, urolophids and *Plesiobatis* (all of them having a fully developed caudal fin), the reduction of the caudal fin to tail folds seems to have been achieved originally in the common ancestor of *Asterotrygon* and *Heliobatis *and later, independently, in more advanced dasyatids. Although the relationship between *Asterotrygon* and *Heliobatis* is weakly supported (Bremer value 1), this might corroborate the hypothesis that the two genera diverged after their common ancestor invaded the freshwater system of Green River Formation, contrary to the hypothesis of Carvalho et al. (2004), who hypothesized that *Asterotrygon* and *Heliobatis* might have invaded independently the Eocene freshwaters of Fossil Lake. It is therefore reasonable to recognize a single monophyletic family, which includes these two extinct genera (Heliobatidae Marsh, 1877). The family Urotrygonidae (*Urotrygon *+ *Urobatis*) is monophyletic as detected in Aschliman, Claeson, et al. (2012), Naylor et al. (2012) and Bertozzi et al. (2016), and sister to a polytomy that includes *Styracura*, freshwater potamotrygonids and dasyatids. Representatives of this grouping are supported by the following traits: presence of a cartilaginous rod in tail (ch. 33[1]); caudal fin absent (ch. 34[2]); first segment of propterygium adjacent to the nasal capsule (ch. 81[2]); cartilage forming component claw in claspers absent (ch. 85[1]); and spiracularis that extends beyond the hyomandibula and Meckel's cartilage (ch. 88[2]). Although a close relationship between the freshwater potamotrygonids and the marine stingray *Styracura* is almost certainly true as detected by morphological, molecular and chrono/geographic evidences (Aschliman, Claeson, et al., 2012; Bertozzi et al., 2016; Carvalho et al., 2004; Carvalho, Loboda, & Silva, 2016; Lovejoy, 1996; Naylor, Caira, Jensen, Rosana, Straube et al., 2012; Naylor, Caira, Jensen, Rosana, White et al., 2012), our phylogeny did not recognize *Styracura* as a genuine member of the family Potamotrygonidae, due to the fact that the *Styracura* lacks some characters of the lateral‐line, and pectoral and pelvic fin skeleton typically found in freshwater potamotrygonids (Carvalho et al., 2016).

**Figure 7:**
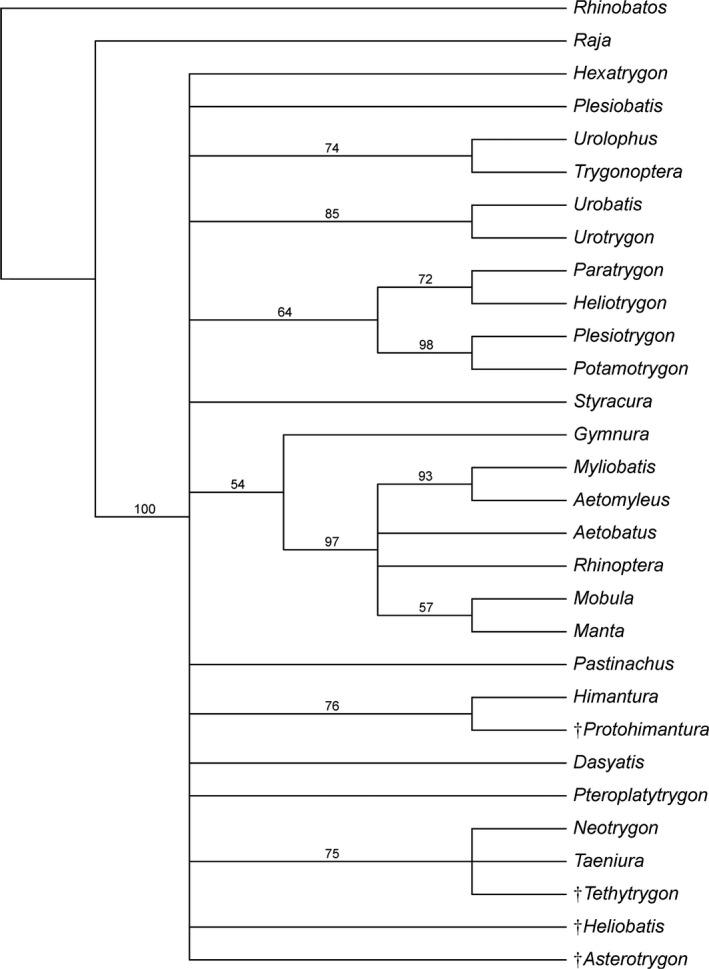
Bootstrap consensus tree based on the same data matrix. Numbers on nodes are bootstrap values. Extinct taxa are marked with a dagger

The monophyletic status of whiptail stingrays of the family Dasyatidae (including here *Himantura*, *Neotrygon*, *Taeniura*, *Pteroplatytrygon*, *Pastinachus* and *Dasyatis*), as recognized by Aschliman, Nishida, et al. (2012), Naylor et al. (2012), Bertozzi et al. (2016), Marramà et al. (2018), but not by Carvalho et al. (2004), Aschliman, Claeson, et al. (2012) and Lim et al. (2015), is recognized and supported herein by two features: ventral terminal cartilage free of axial cartilage (ch. 87[1]) and presence of sexual heterodonty (ch. 89[1]). The presence of tail folds used to diagnose the family Dasyatidae by Bigelow and Schroeder (1953), Compagno and Roberts (1982, 1984) and Nishida (1990) is not supportive of the clade because they are also present in *Styracura*, some freshwater potamotrygonids and extinct heliobatids. The sister‐group relationship between the urogymnines *Protohimantura* and *Himantura* is again recognized as in Marramà et al. (2018) supported by a second transverse tooth keel in these taxa (ch. 99[1]), and mid‐dorsal surface of disc covered by heart‐shaped denticles arranged in an antero‐posteriorly directed patch with sharply defined outlines (ch. 102[1]). The placement of urogymnines as the sister of all other dasyatids is in accordance with molecular analysis presented by Puckridge, Last, White, and Andreakis (2013) but inconsistent with the molecular and morphological phylogenetic results of Lim et al. (2015) and Last, Naylor, et al. (2016). *Tethytrygon* gen. n. is clearly a genuine member of the subfamily Neotrygoninae (including the living *Neotrygon* and *Taeniura*) which is supported herein by three synapomorphies: spiracularis muscle projecting ventrally and posteriorly beyond hyomandibulae and both sets of jaws to insert dorsal to coracomandibularis (ch. 36[1]), presence of anterior process of the Meckel's cartilage (ch. 92[1]), and file of “caniniform” teeth in the upper jaw (ch. 103[1]). It is interesting to note that the monophyly of living neotrygonines has been also detected by the molecular analyses of Aschliman, Nishida, et al. (2012), Puckridge et al. (2013), Lim et al. (2015) Bertozzi et al. (2016) and Last, Naylor, et al. (2016), as well as in the morphology‐based study by Marramà et al. (2018). Although *Tethytrygon* gen. n., *Neotrygon* and *Taeniura* have been resolved in a polytomy, the analysis detected some autapomorphic characters (not shown) useful to distinguish the three genera. For example, *Tethytrygon* gen. n. is unique in the absence of thorns (ch. 69[1]), which instead are present in at least a single antero‐posteriorly directed row of thorns dorsally on disc in living neotrygonine genera (e.g., Last, White, Carvalho, et al., 2016), whereas *Neotrygon* can be distinguished from *Taeniura* in the absence of levator and depressor rostri muscles (ch. 66[0]), and in the presence of a jugal arch (ch. 75[0]). Finally the clade *Pteroplatytrygon *+ (*Dasyatis *+ *Pastinachus*) is recovered here as the most derived clade among dasyatoids, supported by two synapomorphies: pseudosiphon present in claspers (ch. 82[1]) and spiracularis split into lateral and medial bundles, with the medial bundle inserting onto the posterior surface of Meckel's cartilage and the lateral bundle inserting onto the dorsal edge of the hyomandibula (ch. 88[1]). The bootstrap tree (Figure 7) looses resolution as expected, but a close relationship between *Tethytrygon*, *Neotrygon* and *Taeniura* (Neotrygoninae) is still retrieved.

**Figure 8:**
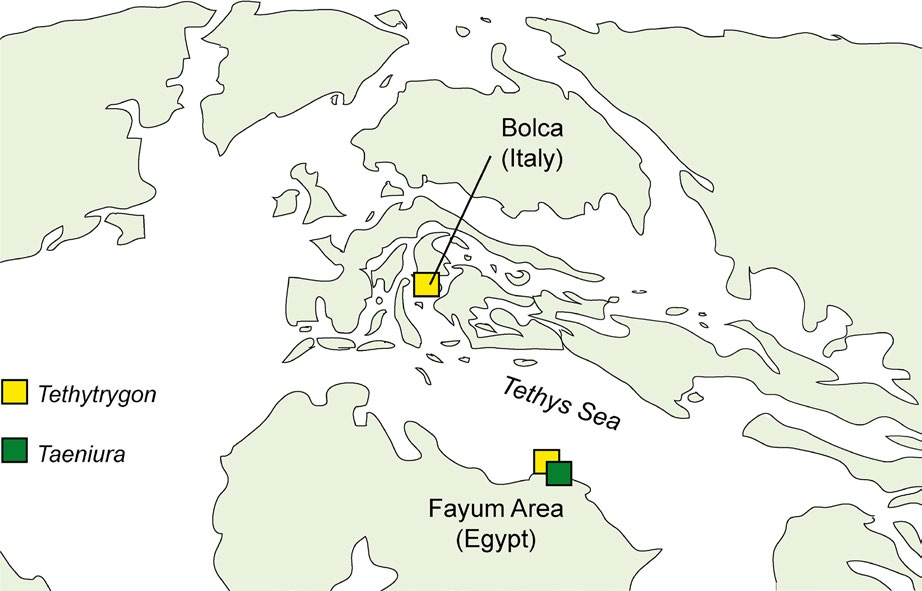
Schematic map of the Tethys area during the Eocene showing the oldest and reliable only occurrences of fossil neotrygonines. Map adopted and modified from Scotese (2002) [Colour figure can be viewed at wileyonlinelibrary.com]

## 5 DISCUSSION

### 5.1 Comparison and relationships

The detailed morphological analysis of *T. muricatus *(Supporting information Appendix S1), has revealed the presence of a number of characters that strongly support its inclusion within the order Myliobatiformes, including the absence of rostral cartilage, presence of a broad and shelf‐like postorbital process, thoracolumbar synarcual, serrated tail sting and basihyal separated from first hypobranchials (e.g., Compagno, 1977; Carvalho et al., 2004; Aschliman, Claeson, et al., 2012). The placement of *T. muricatus *within the Dasyatidae is supported by the ventral terminal cartilage that is free of the axial cartilage, and presence of sexual heterodonty. Moreover, a combination of several plesiomorphic characters argues against the placement of *T. muricatus *in other clades. For example, the presence of tail folds excludes its assignment to myliobatids and dasyatoids characterized by developed caudal fin (e.g., urolophids and urobatis). The absence of angular and secondary cartilages separate the new genus from potamotrygonids, whereas the first segment of the propterygium adjacent to the anterior margin of the antorbital cartilage or anterior to the margin of the nasal capsule separate *T. muricatus *from non‐dasyatids dasyatoids (posterior to the mouth, between mouth and antorbital cartilage, or adjacent to the nasal capsule in these latter). An external margin of the mesopterygium that is more or less straight and not fused to radials exclude any relationship between *T. muricatus* and *Gymnura* (undulated, not fused to radials) or the Urolophidae (highly sinuous, fused with radials; e.g., Carvalho et al., 2004). Moreover, the absence of all the shared derived traits characterizing *Gymnura* and pelagic stingrays (Table 2) supports the exclusion of *Tethytrygon* gen. n. from the group of myliobatoid stingrays.

The morphological and phylogenetic analysis identified *Tethytrygon* gen. n. as a genuine member of the Neotrygoninae, in a polytomous relationship with the extant representatives of this subfamily *Neotrygon* and *Taeniura*. The placement within the subfamily is supported by the presence of the anterior processes of the Meckel's cartilage and the file of “caniniform” teeth in the upper jaw. *Tethytrygon* gen. n. differs from the two living neotrygonine genera by its larger size, longer tail and lower number of trunk vertebrae in addition to morphometric and meristic features (Table 1). *Tethytrygon* gen. n. can be readily separated from *Taeniura* by proportional measurements in disc length, snout to pectoral‐fin insertion, preorbital and preoral length, eye diameter, snout to maximum disc width, and tail length (Table 1). Moreover, the absence of any tooth ornamentation and thorns, and tail folds failing to reach the tip of the tail in *Tethytrygon* gen. n. distinguish it furthermore from *Taeniura*. *Tethytrygon* gen. n. differs from *Neotrygon* in having different proportions of the snout and orbit to pectoral‐fin insertions, pectoral‐fin insertion to sting length, eye diameter, pelvic fin, sting, tail and total lengths, and vertebral counts (Table 1).

### 5.2 Palaeoecology, palaeobiogeography and evolutionary significance

Extant stingrays of the subfamily Neotrygoninae are demersal, benthic marine batoids occurring inshore on continental or insular shelves at depths up to 90 m (Last, White, Carvalho, et al., 2016; Nelson et al., 2016). *Neotrygon* and *Taeniura* mainly inhabit warm‐temperate and tropical shallow waters, and are often associated with the coral reefs of the Indian Ocean, and Indo‐Australian Archipelago, feeding mainly on small bony fishes, crustaceans, worms and bivalves (Last, White, Carvalho, et al., 2016). In this perspective, the presence of several specimens of *Tethytrygon* gen. n., which represents the most common batoid in the Bolca palaeobiotope, suggests close affinities of this taxon with the shallow‐water habitats, possibly associated with coral reefs, hypothesized for the Pesciara palaeobiotope (Marramà, Bannikov, Tyler, Zorzin, & Carnevale, 2016; Papazzoni & Trevisani, 2006).

Although the fossil record of Dasyatidae is extensive and well documented, probably dating back at least to the Early Cretaceous (Cappetta, 2012; Underwood et al., 1999), fossils of the subfamily Neotrygoninae are rare and, with the exception of *Tethytrygon *gen. n., solely represented by isolated teeth. However, the paucity of the fossils probably represents an artefact, since many neotrygonine teeth might have been misassigned to *Dasyatis*, which has been traditionally used as catch‐all genus for many fossil teeth exhibiting “dasyatoid” morphology (Cappetta, 2012; Underwood et al., 1999). Fossils of the genus *Neotrygon* have been reported so far only from the middle to late Eocene deposits of the Fayum area, Egypt. The single tooth figured by Underwood et al. (2011, fig. 7p) is very similar to teeth of *Tethytrygon* gen. nov. Based on palaeobiogeographic, palaeoecological and palaeoenvironmental evidences, we do not exclude that teeth reported as *Neotrygon* sp. by Underwood et al. (2011) may belong to *Tethytrygon* gen. n. Conversely, the genus *Taeniura* was reported from several localities. However, the relative abundance of *Taeniura* in the fossil record from the Miocene to the Pliocene might be an artefact since teeth traditionally reported as *Taeniura grabata* and *Taeniura cavernosa* should be referred to the dasyatid genus *Taeniurops *(subfamily Dasyatinae), recently resurrected by Last and Stevens (2009) based on unambiguous morphological and dental differences with respect to *Taeniura* (see Cappetta, 2012). Thus, reliable reports of *Taeniura* (as *T*. sp.) appear to be solely restricted from the middle to late Eocene of the Fayum area, Egypt (Underwood et al., 2011). Teeth of *Taeniura* sp. are also reported from the lower Miocene of Brazil (Aguilera et al., 2017) and from the Pliocene of Libya (Pawellek et al., 2012), although it is not clear whether the authors recognized their affinities with *Taeniurops *(*T. grabata* or *T. cavernosa*) or *Taeniura *(*T. lymma* or *T. lessoni*). Therefore, the oldest remains referable to neotrygonines are from Eocene tropical shallow Tethyan localities (Bolca and Fayum area; Figure 7).

The Bolca chondrichthyan assemblage is remarkably different from those of other contemporaneous Boreal (London Clay, Paris basin, Lede Sand Formation, Fürstenau Formation, Lillebælt Clay) or Tethyan (SW France and Northern Morocco) deposits, suggesting that its taxonomic composition is largely influenced by the different palaeoenvironmental setting (Marramà et al., 2018). Conversely, the Bolca palaeoenvironmental and palaeoecological characters appear to be more consistent with the tropical shallow settings reported from south‐western Morocco and, even more, with those of the Fayum area in Egypt (Marramà et al., 2018). Like the latter, in particular, the Bolca fauna is characterized by the presence of small odontaspidids, small carcharhinids, and juvenile triakids, all generalist feeders preying on small nectobenthic preys and zooplanktivorous coastal bony fishes. Among batoids, the Fayum area and the Bolca Lagerstätte share the presence of thornbacks (Platyrhinidae) and, as detected in the present study, neotrygonines, which are absent in other deposits (Marramà et al., 2018; Underwood et al., 2011).

Today, the neotrygonines *Neotrygon* and *Taeniura* are restricted to continental and insular shelves of the Indian Ocean and Indo‐Australian Archipelago (Last, White, Carvalho, et al., 2016). Divergence time estimates indicate that living neotrygonines diverged from other dasyatids in the Late Cretaceous and that *Neotrygon* diverged from *Taeniura* around the K‐Pg boundary (Puckridge et al., 2013). However, Aschliman, Nishida, et al. (2012) placed the divergence of neotrygonines from *Dasyatis* around 50 million years ago. Later, a series of rapid cladogenetic events (triggered by tectonics and eustatism) were probably responsible for the isolation and high diversity of *Neotrygon* species in the Indo‐West Pacific area (Puckridge et al., 2013). The authors also suggested an austral origin for the genus *Neotrygon*. Although collecting and taphonomic biases must be considered, since the earliest known neotrygonines appear to be the Ypresian to Priabonian occurrences of Bolca and Fayum area, one can suppose a Tethyan origin for the group and an eastward migration of its representatives from the Tethys during the Eocene, to the Arabian Peninsula and the Indo‐Australian Archipelago in the Miocene, following the shift of the centre of marine biodiversity across the globe from the Eocene to today (Renema et al., 2008). This pattern was also highlighted at least for two other dasyatid subfamilies, the Hypolophinae and the Urogymninae, whose more abundant fossil record indicates an Eocene origination in the Tethys, followed by a widespread colonization of the proto‐ Mediterranean Sea and Indo‐Pacific from late Palaeogene to the early Neogene (Adnet et al., 2018; Marramà et al., 2018).

## 6 CONCLUSIONS

The revision of the Eocene stingrays from the Bolca Lagerstätte traditionally referred to “*Dasyatis*”* muricatus* and “*D*.” *zigni* allowed a detailed reinterpretation of their morphology and taxonomic status. A unique combination of morphological features allowed the recognition of a new genus of the family Dasyatidae, *Tethytrygon* gen. n. The phylogenetic analysis suggested close affinity to the living representatives of the subfamily Neotrygoninae. The scarce fossil record of neotrygonines seems to suggest a Tethyan origin for the group, and that their modern distribution restricted to the Indian Ocean and Indo‐Australian Archipelago may be the final result of their spatial dynamics across the Palaeogene and Neogene, following the eastward shift of the marine centre of palaeobiodiversity across the globe, a model also detected for hypolophines and urogymnines, among stingrays.
