# Supplementary material for: Reappraisal of the Eocene whiptail stingrays (Myliobatiformes, Dasyatidae) of the Bolca Lagerstätte, Italy
Source: Zool Scr. 2018 Nov 28;48(2):168–84. doi: 10.1111/zsc.12330 (PMC6446806; doi:10.1111/zsc.12330)
Supplement: Supplementary file 1 [file ZSC-48-168-s001.pdf]

SUPPLEMENTARY MATERIAL TO:

Reappraisal of the Eocene whiptail stingrays (Myliobatiformes, Dasyatidae) of the Bolca Lagerstätte, Italy

Giuseppe Marramà[1], Giorgio Carnevale[2], Gavin J. P. Naylor[3], Jürgen Kriwet[1]

Eocene whiptail stingrays of Bolca

Marramà *et al*.

[1]University of Vienna, Department of Palaeontology, Althanstrasse 14, 1090, Vienna, Austria
[2] Università degli Studi di Torino, Dipartimento di Scienze della Terra, Via Valperga Caluso 35, 10125 Torino, Italy
[3] University of Florida, Florida Museum of Natural History, 1659 Museum Road, 32611 Gainesville, USA

GEOLOGICAL SETTING

Lithological and sedimentological evidence, as well as the notes in historical archives of museums, suggest that all the specimens included in this study were collected from the fossiliferous layers of the Pesciara site of the Bolca Konservat-Lagerstätte, located in the Lessini Mountains (in southern Alps), about 2 km north-east of the village of Bolca, Verona Province, north-eastern Italy (Fig. S1). Several authors traditionally referred the stratigraphic sequence of the Pesciara site to the 'Calcari Nummulitici', an informal unit of Eocene age widely distributed in north-eastern Italy (Papazzoni & Trevisani, 2006). The succession consists of a less than 20-m-thick cyclic alternation of finely laminated micritic limestones, with exquisitely preserved fishes, plants and invertebrates, and coarse-grained biocalcarenite/biocalcirudite containing a rich benthic fauna. Its benthic foraminiferan content suggests that the fish-bearing limestone of the Pesciara site belongs to the *Alveolina dainelli* Zone or SBZ 11 Biozone (Papazzoni & Trevisani, 2006; Papazzoni et al., 2014), corresponding to the late Cuisian (late Ypresian, about 49 Ma). Results of the quantitative palaeoecological study by Marramà et al. (2016c) allowed a detailed definition of the palaeoecological and palaeoenvironmental setting of the Pesciara palaeobiotope, confirming that the Pesciara fish assemblage was characterized by a sharp oligarchic structure dominated by zooplanktivorous fishes. Taphonomic features confirm that the sediments were deposited in an intraplatform basin in which benthic anoxic conditions and the development of a biofilm acted as promoters of the high-quality preservation of the fossils (Marramà et al., 2016c).

SYSTEMATIC PALAEONTOLOGY

Class Chondrichthyes Huxley, 1880
Superorder Batomorphii Cappetta, 1980
Order Myliobatiformes Compagno, 1973
Family Dasyatidae Jordan, 1888
Subfamily Neotrygoninae Last et al., 2016b
Genus *Tethytrygon* gen. n.

urn:lsid:zoobank.org:pub:38C93935-079D-4517-86EB-E4C0B1A6FAAD

Type species. *Raja muricata* Volta, 1796

Etymology. After Tethys, the ancient sea where the Bolca palaeobiotope was located, and from the ancient Greek word '*trūgṓn*', or Latin '*trȳgōn*', meaning 'stingray'; gender masculine; hence 'a stingray from Tethys'.

Diagnosis. Neotrygonine stingray unique and derived in having the following autapomorphic traits: large size (up to 60 cm DW and possibly 150 cm TL), long tail (170.4 - 184.7% DW), and low number of monospondylous trunk vertebrae (23 - 26). Additionally, *Tethytrygon* gen. nov is also characterized by the following combination of morphological and meristic characters: disc rhombic in shape; disc length 87.2 - 95.2% DW; total length 249.5 - 263.0% DW; subtriangular pelvic fins 24.6 - 29.9% DW; eye diameter 2.7 - 4.5% DW; skin mostly smooth without thorns; small scattered star-shaped dermal denticles only in largest individuals; single serrated sting 26.1 - 32.7% DW; 175 to 179 vertebrae; 108 -117 pectoral radials (49 - 53 propterygial, 16 - 20 mesopterygial, 40 - 45 metapterygial); 24 - 26 pelvic radials; tail folds posterior to caudal sting origin and failing to reach the tip of the tail; enlarged 'caniniform' teeth on upper jaw; teeth rhombic in occlusal view; well-marked concavely arched cutting edges; lingual surface low and strongly concave; crown ornamentation absent; broad and convex basal rim on crown.

Included species. Type species only.

Tethytrygon muricatus (Volta, 1796)

Figs S2-S14

Raja muricata Volta, 1796: 37, pl. 9, fig. 1 (original occurrence of name, description and drawing); Catullo 1827: 334; Woodward 1889: 153.

Trygonobatus vulgaris de Blainville, 1818: 336; Bronn 1831: 8.

Trygon gazzolae Agassiz, 1833-1844: 38; Agassiz 1835: 14; Giebel 1848: 283; de Zigno 1874b: 180.

Anacanthus zigni Molin, 1861: 578; de Zigno 1874a: 12, pl. 2; de Zigno 1874b: 179; Woodward 1889: 153.

Alexandrinum Molin, 1861: 579.

Alexandrinum molinii de Zigno, 1874a: 13, pl. 3; de Zigno 1874b: 181.

Trygon muricatus (Volta, 1796): Jaekel 1894: 142, fig. 32, pl. 4; Eastman 1904: 23; Eastman 1905a: 8; Eastman 1905b: 352; Leriche 1906: 378.

Trygon zignii (Molin, 1861): Jaekel 1894: 145, fig. 33.

Trygon zignoi (Molin, 1861): Leriche 1906: 378.

Trygon muricata (Volta, 1796): Eastman 1911: 316, pl. 43; D'Erasmo 1922: 21; Blot 1980: 344; Frickhinger 1991: 214.

Trygon dezignoi (Molin, 1861): D'Erasmo 1922: 21; Blot 1980: 344.

Dasyatis muricata (Volta, 1796): Cappetta 1987: 163 (misspelt as *D. muricatat*).

'*Dasyatis*' *muricata* (Volta, 1796): Carvalho et al. 2004: 11, fig. 49b; Carnevale et al. 2014: 41, fig. 2b; Marramà et al. 2018b: 287, fig. 7a-b.

'*Dasyatis*' *dezignoi* (Molin, 1861): Carvalho et al. 2004: 11; Carnevale et al. 2014: 41.

'*Dasyatis*' *zigni* (Molin, 1861): Marramà et al. 2018b: 287, fig. 8a-b.

Holotype. MNHN F.Bol.564, nearly complete articulated skeleton lacking the terminal portion of tail, in part and counterpart; 282.0 mm DW (Fig. S2).

Referred material. MGP-PD 159/160, nearly complete articulated skeleton, in part and counterpart, 283.0 mm DW (Fig. S3); MGP-PD 150Z/151Z, incomplete articulated skeleton lacking most of the tail, 143.3 mm DW (Fig. S4); CMNH 4521, nearly complete articulated skeleton, in part and counterpart, 203.9 mm DW; CMC2, nearly complete skeleton, 146 mm DW (Fig. S5A); MCSNV IG.23194, nearly complete articulated skeleton, 603.0 mm DW (Fig. S5B); MCSNV IG.186653, nearly complete articulated skeleton, 281.9 mm DW (Fig. S5C); MCSNV T.1020/1, nearly complete articulated skeleton, in part and counterpart, 288.7 mm DW (Fig. S5D); MCSNV VII.B.92/3, nearly complete articulated skeleton, in part and counterpart, 251.4 mm DW (Fig. S5E); MCZ 13183, incomplete and poorly preserved articulated skeleton, 122.1 mm DW; MGGC 7456, nearly complete disarticulated skeleton in a single slab, 288.7 mm DW (Fig. S6); MNHN F.Bol.568, incomplete articulated skeleton, in part and counterpart (Fig. S5F); MNHN F.Bol.584, incomplete disarticulated skeleton.

Type locality and horizon. Pesciara site, Bolca Konservat-Lagerstatte, Italy; early Eocene, late Ypresian, middle Cuisian, SBZ 11, *Alveolina dainelli* Zone (see Papazzoni et al., 2014).

Diagnosis. As for the genus.

Description

Tethytrygon muricatus (Volta, 1796) is represented by 13 partially complete and articulated skeletons, most of them lacking the distal portion of tail; the latter is preserved and recognizable in MCSNV T.1020/1 (Fig. S5D), MCSNV VII.B.92/3 (Fig. S5E), and MGGC 7456 (Fig. S6). The large number of available specimens and their good preservation allowed the recognition and description of several skeletal and dental characters, which are useful to distinguish and separate the taxon from any other known living and fossil dasyatid. Counts and measurements for *T. muricatus* are listed in Table S1. The specimens examined comprise different ontogenetic stages, with the largest one (an adult male lacking the distal portion of the tail) being characterized by 60 cm disc width and possibly reaching 150 cm in total length.

The disc of *Tethytrygon* gen. nov. is rhombic, not wing-like, as detected in MCSNV IG.23194, MGP-PD 150Z/151Z, MGGC 7456, reaching the maximum width in the anterior third of disc length. However, in most of the specimens the disc mostly appears ovoid or circular in shape, likely due to the poor preservation of the distal-most pectoral-fin radials or inaccurate preparation of the historical specimens. The disc length is slightly shorter than the disc width (0.9 times), whereas the total length is about 2.6 and 2.8 times those of the disc width and disc length, respectively. The tail is long and about 1.8 times the disc width. *Tethytrygon muricatus* lacks dorsal fins, whereas a single serrated sting can be recognized in most of the specimens. The skeleton is highly calcified in large individuals and most of the skeletal elements, including jaws, hyomandibulae, synarcuals, pectoral and pelvic girdles, show the typical prismatic calcification of elasmobranchs (Dean & Summers, 2006).

Neurocranium. The neurocranium is antero-posteriorly elongate, longer than wide, with the greatest width at the level of the nasal capsules (Figs S7-S8). The rostral cartilage is absent as in all adult stingrays (e.g., Compagno, 1977; Miyake et al., 1992). The nasal capsules are transversely broad and ovoid in shape. Their anterior margin is rounded and biconvex with a small and triangular anterior median indentation, but lacking the anterior process of the neurocranium typical of *Rhinoptera* and *Mobula*. The preorbital processes are small, posteriorly directed, and protrude from the posterolateral aspect of nasal capsules (Fig. S8). The supraorbital process is difficult to detect, whereas the postorbital processes is long, distally broad and shelf-like (Fig. S7). Foramina for the rami of the superficial ophthalmic nerve through the supraorbital crest are not visible in the available specimens. The orbital region is longer than wide. The neurocranium has its smallest width at the level of the otic region; the narrowest part of the neurocranium is about 3.5 times in the

neurocranial length. The otic capsules are robust and short, and provide articulation for the proximal portion of the hyomandibulae. The dorsal fontanelle is visible in dorsally exposed specimens as MCSNV VII.B.92/93 (Fig. S8). It is antero-posteriorly elongated and covers about 70-80% of the neurocranial length. The fontanelle exhibits a median constriction, which represents a remnant of the epiphysial bar of stingrays that separates an anterior precerebral fontanelle from a posterior fronto-parietal fontanelle, resembling the condition of *Taeniura* and *Neotrygon* among dasyatids (Fig. S9) (Miyake, 1988; Rosenberg, 2001; Carvalho et al., 2004). A pair of internal carotid artery foramina is located on the ventral surface of the neurocranium in MGP-PD 150Z/151Z (Fig. S7), like in most of the stingrays (Nishida, 1990). The antorbital cartilages are thin and long, not branched and subtriangular in shape. Their maximum width is located at the level of the articulation with the nasal capsules. They taper distally and articulate with the propterygia through their external margin.

Jaws. The jaws of *Tethytrygon* gen. n. are massive and robust, being reinforced with prismatic calcification as in most stingrays (Fig. S7). Upper and lower jaws extend laterally and occupy almost the entire space between the propterygia. Their antimeres are narrow and separated at the symphysis. The occlusal width appears to be greater than the diastema width. In smaller individuals the outlines of the jaws appear to be gently curved, whereas the contours are more squared and delineated in adult individuals.

The palatoquadrate is labio-lingually compressed, narrower and smaller than Meckel's cartilage, and is relatively straight on its dorsal flange, resembling the condition in potamotrygonids, *Dasyatis*, *Himantura*, *Taeniura*, and urolophids (Carvalho et al., 2004). Small anterior processes of the Meckel's cartilage can be recognized at least in the holotype MNHN F.Bol.564 and in MGP-PD 150Z/151Z, MCSNV T.1020/1 and MCSNV IG.186653, therefore resembling the condition of some dasyatoids, including *Trygonoptera*, *Taeniura* and *Neotrygon* (Underwood et al., 2017). By contrast, the medial symphyseal processes typical of *Dasyatis* and *Himantura* (Underwood et al., 2017), or the ventro-lateral processes of the mandibular cartilage present in *Asterotrygon*, *Taeniura*, potamotrygonids, and other non-myliobatid stingrays (Carvalho et al., 2004) are difficult to discern in the available material. However, the lateral projections of the lower jaws ('wing-like processes' of Carvalho et al., 2004) typical of myliobatids, are clearly absent in *Tethytrygon* gen. n.

Hyoid and gill arches. Although the hyomandibulae are slender when observed in dorso-ventral view and stouter when observed from a lateral perspective in living stingrays (Carvalho et al.,

2004). In *Tethytrygon* gen. n. they appear relatively stout and robust in most of the specimens (Figs S7-S8). The hyomandibulae are slightly arched and with a concave inner margin, compressed and narrow at about their midlength. The hyomandibulae project antero-laterally, reaching the mesial wall of the propterygia just posterior to the postero-ventral corner of the lower jaw. The distal end of the hyomandibulae articulates with the lower jaw through a strong and stout terminal portion, whereas their proximal portion at the articulation with the otic region of the neurocranium is enlarged and stouter than its mesial part. There is no trace of the angular cartilages typical of potamotrygonids or of the secondary hyomandibular cartilages characteristic of *Urolophus* and pelagic stingrays (see Lovejoy, 1996; Carvalho et al., 2004, Claeson et al., 2010). The ventral gill arches of *Tethytrygon* gen. n. appear to be partially preserved in some of the specimens and their morphology consistent, at least in part, to that of *Dasyatis*, *Himantura* and *Taeniura* as documented in Miyake & McEachran (1991, fig. 8), and Carvalho et al. (2004, fig. 35A). The outline of the central medial plate, which results from the fusion of the basibranchial copula and the basibranchial components (Miyake & McEachran, 1991; Carvalho et al., 2004), can be observed at least in MGP-PD 159/160, and MGP-PD 150Z/151Z (Fig. S7). The medial plate appears tubular and slightly compressed laterally. Its posterior distal tip seems to taper into small median projections. The basihyal appears to be segmented in at least two small plates that are clearly separated from the first hypobranchials, these latter appearing considerably stout and robust. There are five pairs of ceratobranchials. The first one is fused to the pseudohyoid, as in most of the dasyatoids whereas all the other ceratobranchial pairs articulate with small rami along the lateral margin of the medial plate. The last two ceratobranchials appear ankylosed to each other but not fused in their proximal portion. The fifth ceratobranchial pair articulates with the anterior margin of the scapulocoracoid. Filamentous branchial rays associated with the ceratobranchials can be recognized in most of the specimens that preserve the hyoid region, although their number on each ceratobranchial is difficult to detect.

Synarcuals and vertebral column: Anterior (cervicothoracic) and posterior (thoracolumbar) synarcual cartilages are both preserved and easy to recognize. The cervicothoracic synarcual is particularly strongly calcified as detected by its prismatic calcification, and articulates with the occipital condyles of the chondrocranium. The dorsal medial crest of the anterior synarcual runs antero-posteriorly along almost its entire length, and can be recognized at least in the dorsally exposed specimen MCSNV VII.B.92/93 (Fig. S8). The distal portions of lateral stays of the cervicothoracic synarcual are visible in the same specimen. They are located in the posterior half of the synarcual, and possibly formed in origin a U-shaped structure, as in all myliobatiforms

(Aschliman et al., 2012a). It is not possible to determine the synarcual foramina or the number of fused vertebrae that constitute the first synarcual. The thoracolumbar synarcual, which is uniquely present in stingrays, articulates anteriorly with the anterior synarcual but contrary to this cervicothoracic, it has a simpler structure, being triangular in shape and tapering posteriorly. The second synarcual is as long as the first synarcual, ending at about midlength between the scapulocoracoid and pelvic girdle. About 10-15 unfused individual vertebral centra can be recognized throughout the vertebral column length. The vertebral column of *Tethytrygon* gen. n. consists of about 175-179 vertebral centra. There are 23-26 trunk centra (from the first distinguishable centrum to the anterior margin of the puboischiadic bar), 100-109 can be recognized from the anterior margin of the puboischiadic bar to sting origin, and 45-54 are those posterior to the sting origin. The vertebral centra are small, subrectangular in shape and antero-posteriorly short. Neural spines are visible in the abdominal cavity just anteriorly and posteriorly to the pelvic girdle in most of the specimens. In adult individuals, the neural spines are long, laterally compressed, and postero-obliquely oriented in relation to the centra, whereas their length is proportionally reduced in juveniles. Ventral arches, being much smaller in comparison to the neural arches in living stingrays (Carvalho et al., 2004) are difficult to distinguish in the examined material. The distal portion of the vertebral column posterior to the caudal sting is stiffened by the presence of a cartilaginous rod, which is typically present in dasyatids, potamotrygonids and pelagic stingrays (Carvalho et al., 2004), conferring a straight and rigid posture to the tail. The ribs are absent, like in all myliobatiforms (McEachran et al., 1996; Aschliman et al., 2012a).

Pectoral fins and girdle. As in all stingrays, the scapulocoracoid of *Tethytrygon* gen. n. is composed of a transverse ventral coracoid bar, and lateral, dorsally projecting scapular processes. The coracoid bar is well preserved in ventrally exposed specimens as a transverse structure in the middle of the disc (Fig. S7). The coracoid bar is a single straight and robust transverse bone, located ventral to the cervicothoracic synarcual, and just anterior to the articulation between the two synarcuals. The length of its lateral margins is contained about 2-2.5 times in its width. The scapular processes are best preserved in dorsally exposed specimens (Fig. S8), where they appear as large subtriangular structures, obliquely oriented in relation to the synarcual cartilage, and articulating with this latter through large articular surfaces via the so called ball-and-socket articulation, a unique feature of stingrays (Compagno, 1973, 1977; Carvalho et al., 2004). The scapular fossa (or foramen) cannot be recognized in the available material. In *Tethytrygon* gen. n. the suprascapulae are fused to the median crest of the cervicothoracic synarcual (Fig. S8) and are possibly continuous with the

posterior margin of the lateral stays, creating a well-defined bridge that is unique to stingrays (Miyake, 1988; Lovejoy, 1996; Aschliman et al. 2012a).

Laterally, the scapulocoracoid bar articulates with the internal skeleton of the pterygia. The propterygium is long, arched, tapering distally and extending to the anterior disc margin. The ridge and the sulcus for the insertion of the pectoral fin muscles can be recognized along almost the entire length of the propterygium in several specimens. The propterygium is distally segmented and the first small segment is adjacent to the nasal capsules resembling the condition observed in the stingray genera *Hexatrygon*, *Neotrygon*, *Taeniura*, *Styracura*, and *Potamotrygon* (Lovejoy, 1996, fig. 10; Rosenberger, 2001, fig. 2; Aschliman et al. 2012a). The proximal portion of the propterygium is large, articulating with the anterior portion of the lateral margin of the scapulocoracoid, and with the anterior mesial margin of the mesopterygium, contrary to the condition of *Plesiotrygon* and *Potamotrygon* in which this latter articulation is absent (Lovejoy, 1996). The mesopterygium is a single, small and subtriangular bone, whose external margins are more or less straight and not fused to the radials. The metapterygium is slightly shorter and more slender than the propterygium, arched and tapering posteriorly, ending slightly posteriorly to the anterior margin of the puboischiadic bar. There are about 108-117 pectoral radials of which 49-53 are propterygial, 16-20 mesopterygial, and 40-45 metapterygial. Each radial is composed of at least 20 segments and bifurcates at least once. The radials of *Tethytrygon* gen. n. are calcified in a chain-like patterns, forming the so-called ‘catenated calcification’ typical of batoids with undulatory swimming mode, including most of the benthic stingrays, except *Plesiobatis* (Schaefer & Summers, 2005). Furthermore, *Tethytrygon* gen. n. does not show the condition observed in *Gymnura* and myliobatids (see Nishida, 1990; Schaefer & Summers, 2005) in which the pectoral radials have laterally expanded elements that articulate with the adjacent radials.

Pelvic girdle and fins. The pelvic fins are single-lobed, protruding well beyond the disc, and with almost pointed apices. Their length equals about 28% of disc width. The anterior margin of the pelvic fins is straight and partially overlapped by the posterior margin of the pectoral fins. The puboischiadic bar (Fig. S10) is robust, relatively wide (about 23% of disc width), and only moderately arched, forming an obtuse angle of about 130-140° at its mid-length, although the condition is not comparable to the strong arching of the puboischiadic bar in *Gymnura*, *Rhinoptera* and *Mobula* (Carvalho et al., 2004). The bar is enlarged at its distal corners, where one to four obturator foramina can be recognized in most of the specimens. The long median prepelvic process typical of freshwater potamotrygonids is clearly absent, although a small and short triangular process similar to those of most dasyatids might be present, although mostly obscured by vertebrae.

Small and triangular ischial processes are present on the inner corners of the posterior margin of the puboischiadic bar, although they are not as developed as in potamotrygonids. The iliac processes are preserved in most of the specimens, although they are sometimes partially obscured by the basipterygium or by the pelvic radials. The iliac processes are quite developed, and project posteriorly from the posterior corners of the puboischiadic bar. They are elongated, slightly curved and tapering posteriorly, with the distal tips projecting mesially. The basipterygia are slightly shorter than the puboischiadic bar width, and are more or less straight or with a slightly concave inner margin. Each basipterygium supports about 24-27 pelvic fin radials (mostly 25). The first radial, which supports the anterior margin of the pelvic fin, is enlarged and articulates with the lateral aspect of the puboischiadic bar. As in all myliobatiforms, the first radial is band-like, slightly expanded distally, and articulating with two radial segments in a parallel way. Each pelvic-fin radial bifurcates distally once. A single specimen (MCSNV IG.23194) shows two elongate claspers (about 19% DW), articulating with the distal tip of the basipterygium (Fig. S11). Their apex is enlarged and pointed, extending beyond the posterior margin of the pelvic fin lobes. The axial cartilage is thin, straight and represents the longest element of the clasper. However, its length does not extend beyond the posterior margin of the ventral and dorsal terminal cartilages, resembling the condition of *Taeniura* (see Aschliman et al. 2012a, fig. 3.6). In the apex it is possible to recognize proximally the small dorsal and ventral marginal cartilages. Distally the larger dorsal and ventral terminal cartilages form the external-most components of the clasper apex. It is not possible to recognize the presence of a pseudosiphon or the presence of medial flange in the dorsal marginal cartilage. The dorsal terminal cartilage clearly shows the crenate margins that are typical of several dasyatids, whereas the ventral terminal cartilage appears to be free of axial cartilage at least along its distal-most portion, thereby supporting the placement of *Tethytrygon* gen. n. within the family Dasyatidae in the phylogenetic analysis.

Dorsal and caudal fins. Dorsal fins are usually absent in dasyatids, and this condition is also clearly observable in *Tethytrygon* gen. n. given that the specimens do not show any appreciable structures (i.e., radials) anteriorly to the sting that may suggest the presence of a dorsal fin. A completely developed caudal fin is also absent as is the case in many stingrays, and the caudal fin of *Tethytrygon* gen. n. is reduced to tail folds. In extant dasyatids, the tail region posterior to the caudal stings exhibits small dorsal and ventral elements (rudimentary radials of Nishida, 1990) reminiscent of rudimentary radial elements supporting dorsal and ventral folds. The presence of tail folds, possibly ventrally and dorsally to the tail, is clearly observable in specimen MGGC 7456, in which the UV light emphasized the presence of dark pigmented structures along both sides of the vertebral

column posterior to sting and that do not reach the tip of the tail (Fig. S6C-D). It is interesting to note that the presence of tail folds in *Tethytrygon* gen. n. was also noticed by Jaekel (1894; "[…] *comb of well-developed lower spinous processes on the tail speaks for the original presence of a ventral longitudinal fin*") who tentatively hypothesized that the specimens might have been referred to *Taeniura* (see synonymy). However, contrary to *Taeniura* in which the tail folds extend to tip of the tail, *Tethytrygon* gen. n. seems to show the condition characteristic of *Neotrygon* and most dasyatids, in which the tail folds fail to reach the end of the tail (see Last et al., 2016a).

Dentition. The teeth of *Tethytrygon* gen. n. are small, with crown width up to 1.5 mm and arranged in up to 50 rows and about 10 files per jaw in the largest specimens (e.g., MCSNV IG. 23194). However, these values may be subject to ontogenetic variation and cannot be determined in several specimens due to inadequate preservation. The dentition is gradient monognathic heterodont with lateral teeth becoming smaller and enlarged labio-lingually. Sexually dimorphic heterodonty is recognized based on the analysis of the dentition of the only male, which presents longer cusps in all teeth. Ontogenetic heterodonty is present, revealed by the cusps of the teeth of juveniles (e.g., MGP-PD 150Z/151Z) that are not as long as in adult individuals with the crown more rounded in occlusal view.

We were able to isolate two single teeth from MCSNV IG.186653 (an adult female) one of which comes from the file with 'caniniform' teeth (Fig. S12). The presence of enlarged 'caniniform' teeth is diagnostic of neotrygonines (Last et al., 2016a, b) and supports the grouping of *Tethytrygon* gen. n. with *Taeniura* and *Neotrygon* in our phylogeny. The fact that MCSNV IG.186653 is recognized as an adult female (claspers are absent) excludes a priori the hypothesis that the morphology of the 'caniniform' tooth extracted from the specimen is due to sexual dimorphism, usually revealed in other stingrays by longer tooth cups in males. 'Caniniform teeth' are also present in female neotrygonine stingrays (e.g., Nishida, 1990, fig. 26; Cappetta, 2012; Underwood et al., 2015, fig. 2H). The tooth morphology is consistent, at least in part, with that of *Neotrygon kuhli* figured by Herman et al. (1998, pls 1-3 as *Amphotistius kuhli*) and, even more, with the tooth referred to *Neotrygon* sp. from middle-late Eocene of Egypt figured by Underwood et al. (2011, fig. 7P). In occlusal view, the teeth are rhombic in outline. The occlusal surface is not completely flat but undulated. The large 'caniniform' teeth are mesio-distally more elongate, and present a very large cusp that is as long as the crown width. The 'caniniform' cusp overhangs the lingual margin of the crow and is slightly distally directed. The teeth show well-marked concavely arched cutting edges that separate the labial and lingual crown surfaces. The lingual surface is low and strongly concave, whereas in labial view the crown appears extremely narrow and convex.

Crown ornamentation is completely absent, resembling the condition in teeth of *Neotrygon*. The basal view of the crown shows a broad and convex crown rim, which is larger at the distal margin, gradually narrowing toward the mesial margin. The crown-root junction is located in a depression at the centre of the basal surface of the crown. The root is only partially preserved in the examined teeth and only in its proximal part. However, it is probably holaulacorhizous and with two lobes. Root foramina and coating are not detectable.

Squamation and sting. Most of the specimens lack dermal denticles and thorns. However, some of the largest adult individuals dorsally exposed (e.g., MGGC 7456) show very small and scattered star-shaped dermal denticles on the dorsal disc. Depending on the species, *Taeniura* and *Neotrygon* can have or lack dermal denticles, although at least a single antero-posteriorly directed row of thorns dorsally on disc is usually present in *Taeniura* (Last et al., 2016a). A single serrated caudal sting is present in most of the specimens (Fig. S13). The sting is elongate, dorso-ventrally flattened and tapers toward the apex. The caudal sting origin is at about mid-length of the tail, and at about 60% of the total body length in specimens preserving the tip of the tail. The sting length is about 30% of the disc width. Serrations are present along both sides of the sting and their total number varies with ontogeny. In our sample, this number ranges from 48 in smaller specimens to about 90 in the largest ones, with an average total number of about 70 (ca. 35 per side) serrations. The serrations are small, hook shaped, and directed transversely to the main axis of the caudal sting, forming with it an acute angle varying from 27°(in smaller) to 23° (in adult) individuals.

Soft tissues and gut content. Just posteriorly to the nasal capsules, most of the specimens preserve traces of the eyes as brown-coloured carbon films contoured by their optic capsules (Fig. S14A). However, in some specimens the eyes are hidden and obscured by the strongly calcified distal extremities of the upper and lower jaws. The eyeballs are mostly rounded in shape, or slightly antero-posteriorly elongated, and possibly consist of a dense accumulation of lens pigments exhibiting a pattern very similar to that reported in modern and fossil elasmobranchs (Zigman, 1991; Hueter et al., 2004; Vullo et al., 2016; Marramà et al., 2018a, c).

The presence of further soft tissues can be recognized in the abdominal cavity in most of the specimens. In the area posterior to the scapulocoracoid and between the metapterygia, a large semi-oval or crescent-shape reddish or brownish area is interpreted as representing the residue of the liver, occupying the same anatomical position of this organ in extant stingrays (Fig. S14B-D). The preservation of traces of the liver can be explained by its rich iron content, which provides a reddish

or brownish colour to the limestone matrix, as reported in other fossil vertebrates (e.g., Dal Sasso & Maganuco, 2011; Martin et al., 2015).

A single specimen (MNHN F.Bol.584) shows abdominal gut content consisting of a single partial vertebral column of about 40 mm of a bony fish, including associated arches, ribs and intermuscular bones (Fig. S14E-F). The vertebral column of this bony fish is totally preserved in the abdominal cavity of the stingray between the pectoral and pelvic girdles on one side of the vertebral column, in a position comparable to that occupied by the gut-intestine tract in extant stingrays (Fig. S14D). The bones of the head and caudal region of the bony fish appear disarticulated and dispersed around the vertebral column, therefore showing evidence of digestion and suggesting that consumption occurred well before the death of the stingray. Due to the considerable degree of disarticulation of the head skeleton, it was not possible to detect the taxonomic identity of the bony fish, although its small size and general physiognomy of the vertebral centra appear to be comparable to those of clupeoid fishes (i.e. sardines, anchovies), which are the most common fishes of the Pesciara palaeobiotope (Marramà & Carnevale, 2015a, b, 2016, 2018; Marramà et al., 2016c). This suggests that the food preference of *Tethytrygon* gen. n. were similar to those of extant neotrygonines (Last et al., 2016a), and that this Eocene stingray possibly occupied the same ecological niche.

Biometric remarks

A regression analysis in which morphometric characters were regressed against disk width supported the hypothesis that only a single taxon should be recognized within the genus *Tethytrygon* gen. n. The least square regressions of the morphometric characters (Figs S15-S16; Table S2) were useful in demonstrating that the specimen MGP-PD 150Z/151Z, traditionally regarded as a different species ('*Dasyatis*' *zigni*), readily fits within the ontogenetic series of *T. muricatus*. All regressions show high coefficient of determination ranging from 0.76 to 0.99, indicating a very good alignment of the points around the line and, consequently, suggesting a significant real dependence between the disc width and each morphometric character ($p < 0.05$). Although the regressions between disc width with pelvics to tip of tail length, tail and total length also show high coefficient of determinations, their relationships are not significant ($p > 0.05$), but this is probably due to the reduced number of specimens (three) showing these characters. Both the holotype of *Tethytrygon muricatus* (MNHN F.Bol.564), and the holotype of '*Dasyatis*' *zigni* (MGP-PD 150Z/151Z) always fall within the cloud of points and near the regression lines, mostly within the 95% confidence interval, thereby suggesting that morphometric characters are not supporting

differentiation of two different species, and that MGP-PD 150Z/151Z rather represents a juvenile individual within the ontogenetic series of *T. muricatus*. Thus, since no other reliable morphological or meristic characters are useful to distinguish the two species (see also Remarks), '*Dasyatis*' *zigni* should be consequently regarded as a junior synonym of *Tethytrygon muricatus* (Volta, 1796).

FIGURES AND TABLES

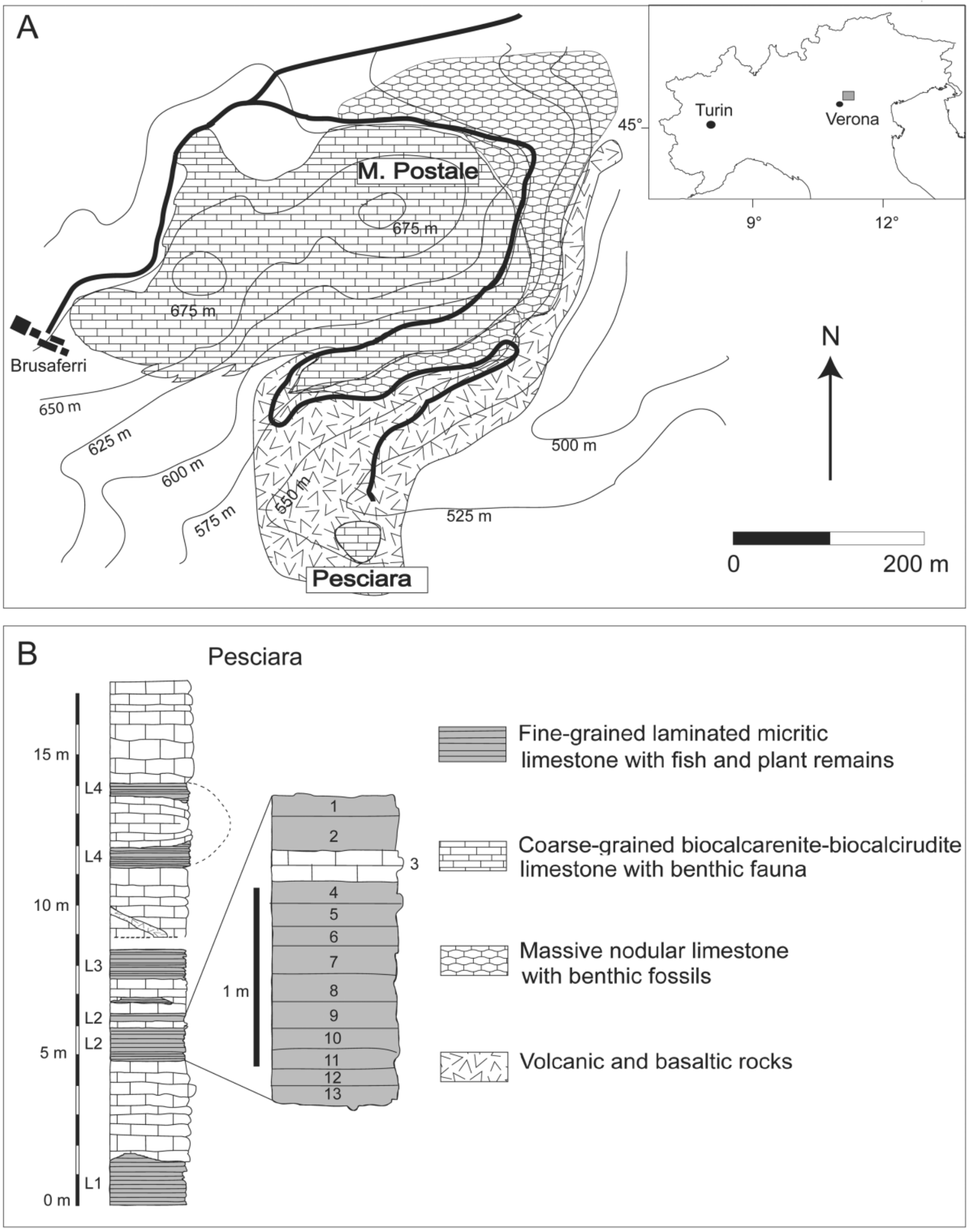

Fig. S1. A. Location (North Italy) and geological map of the Bolca area - B. Stratigraphic section of the Pesciara sequence and legend. Adopted and modified from Papazzoni & Trevisani (2006), Trevisani (2015) and Marramà et al. (2016c).

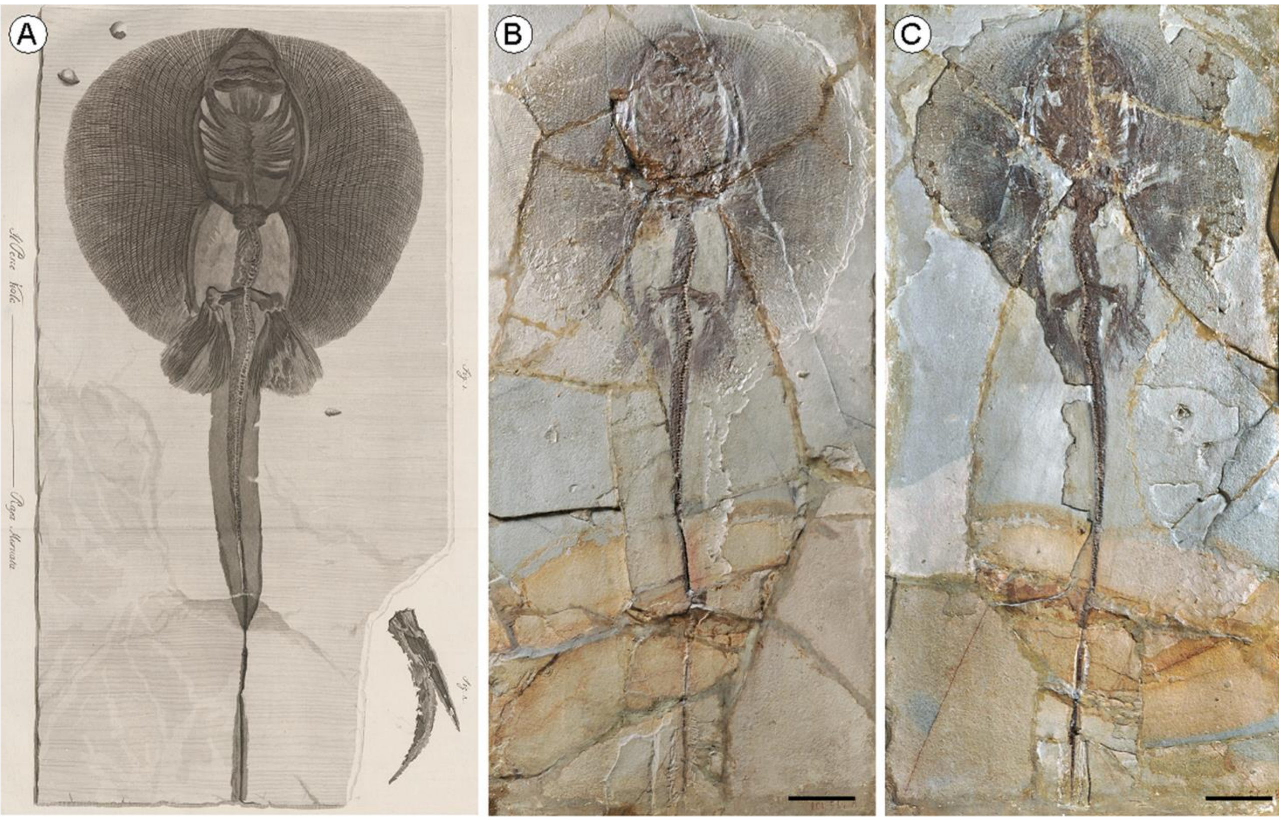

Fig. S2. A-C. *Tethytrygon muricatus* (Volta, 1796) from the Eocene of Bolca Lagerstätte - A. Historical plate of the holotype of *T. muricatus* MNHN F.Bol.564 illustrated and specified as *Raja muricata* in Volta (1796, pl. 9); photo: courtesy of Roberto Zorzin and Museo Civico di Storia Naturale di Verona - B, C. The holotype MNHN F.Bol.564 in part and counterpart - Scale bars 50 mm.

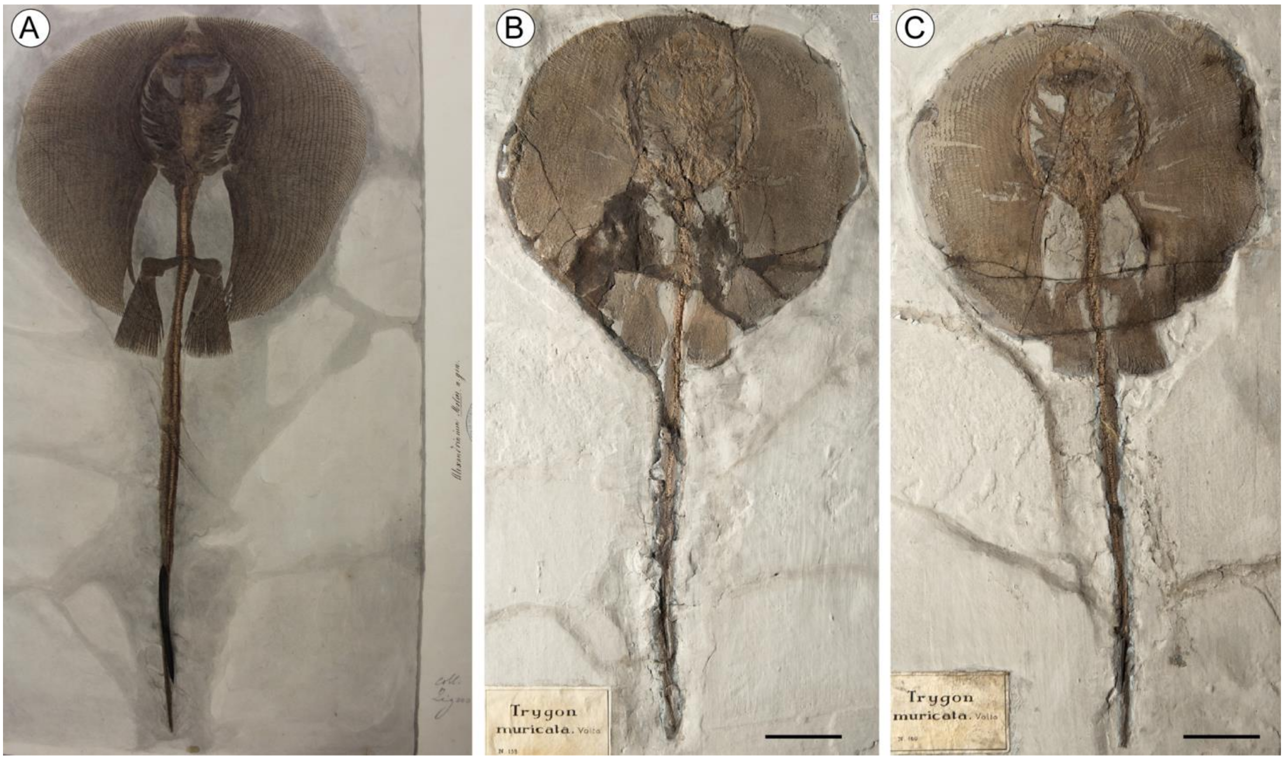

Fig. S3. A-C. *Tethytrygon muricatus* (Volta, 1796) from the Eocene of Bolca Lagerstätte - A. Unpublished plate of the specimen MGP-PD 159Z/160Z illustrated and specified as *Alexandrinum molinii* by Achille de Zigno (1813-1892); photo: courtesy of Università degli Studi di Padova - B, C. Part and counterpart of the specimen MGP-PD 159Z/160Z - Scale bars 50 mm.

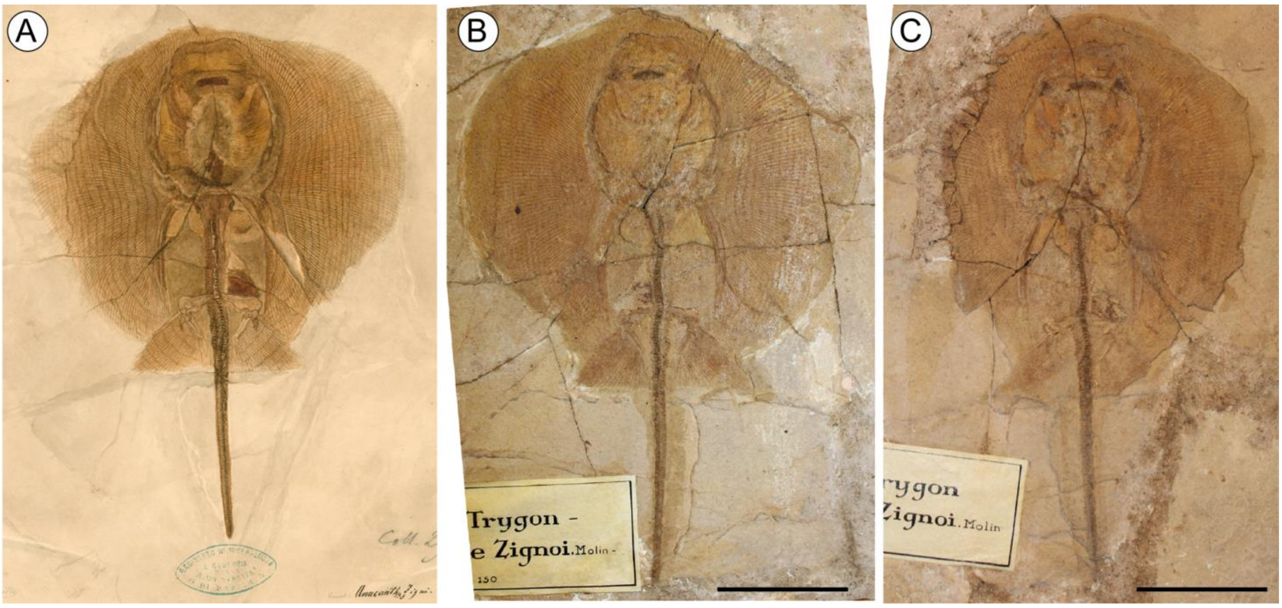

Fig. S4. A-C. *Tethytrygon muricatus* (Volta, 1796) from the Eocene of Bolca Lagerstätte - A. Unpublished plate of the specimen MGP-PD 150Z/151Z illustrated and specified as *Anacanthus zignii* by Achille de Zigno (1813-1892); photo: courtesy of Università degli Studi di Padova - B, C. Part and counterpart of the specimen MGP-PD 150Z/151Z - Scale bars 50 mm.

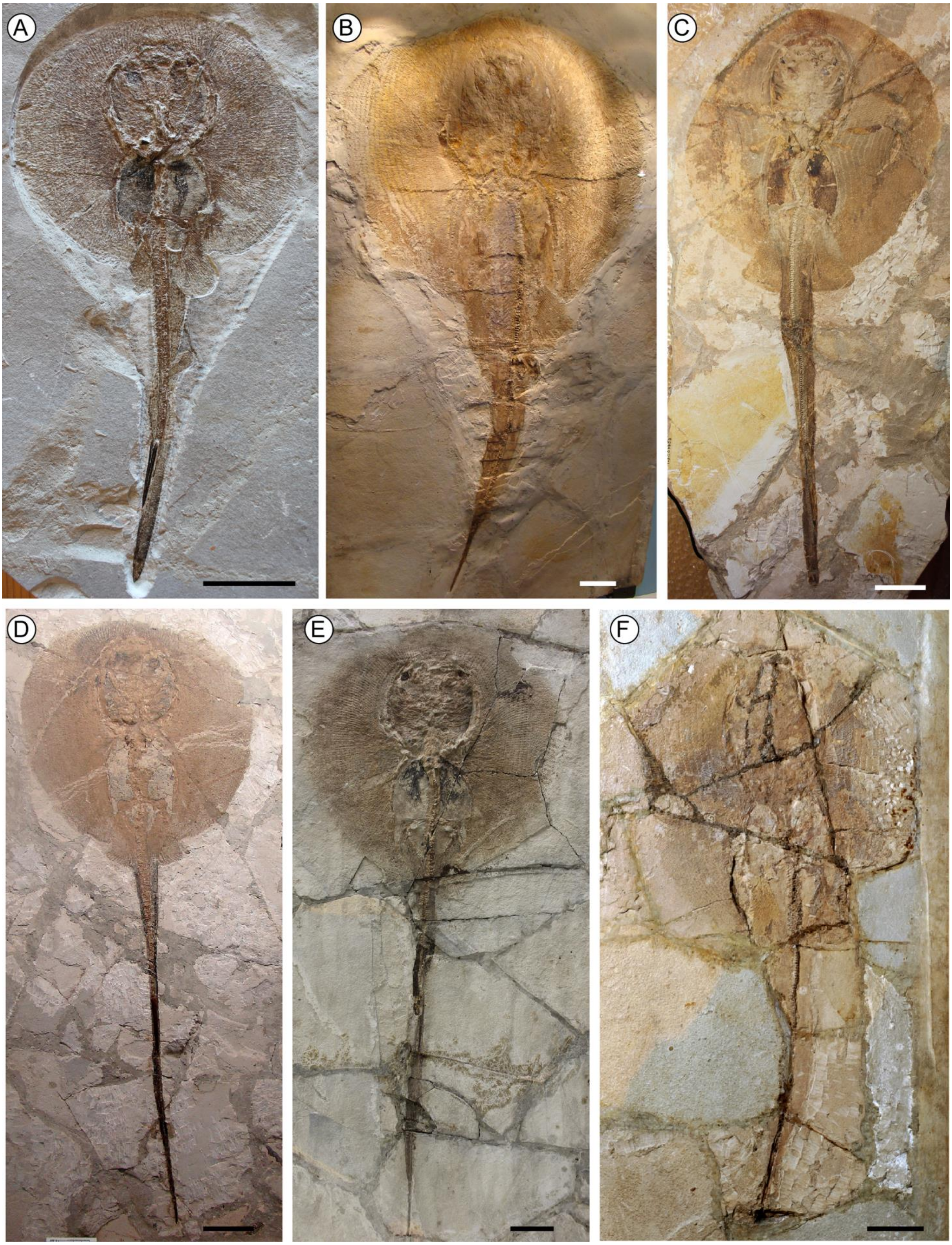

Fig. S5. A-F. Selected specimens of *Tethytrygon muricatus* (Volta, 1796) from the Eocene of Bolca Lagerstätte - A. CMC2, juvenile female individual - B. MCSNV IG.23194, adult male - C. MCSNV IG.186653, adult female - D. MCSNV T.1021, subadult female - E. MCSNV II.B.92, subadult female - F. MNHN F.Bol568, adult female - Scale bars 50 mm.

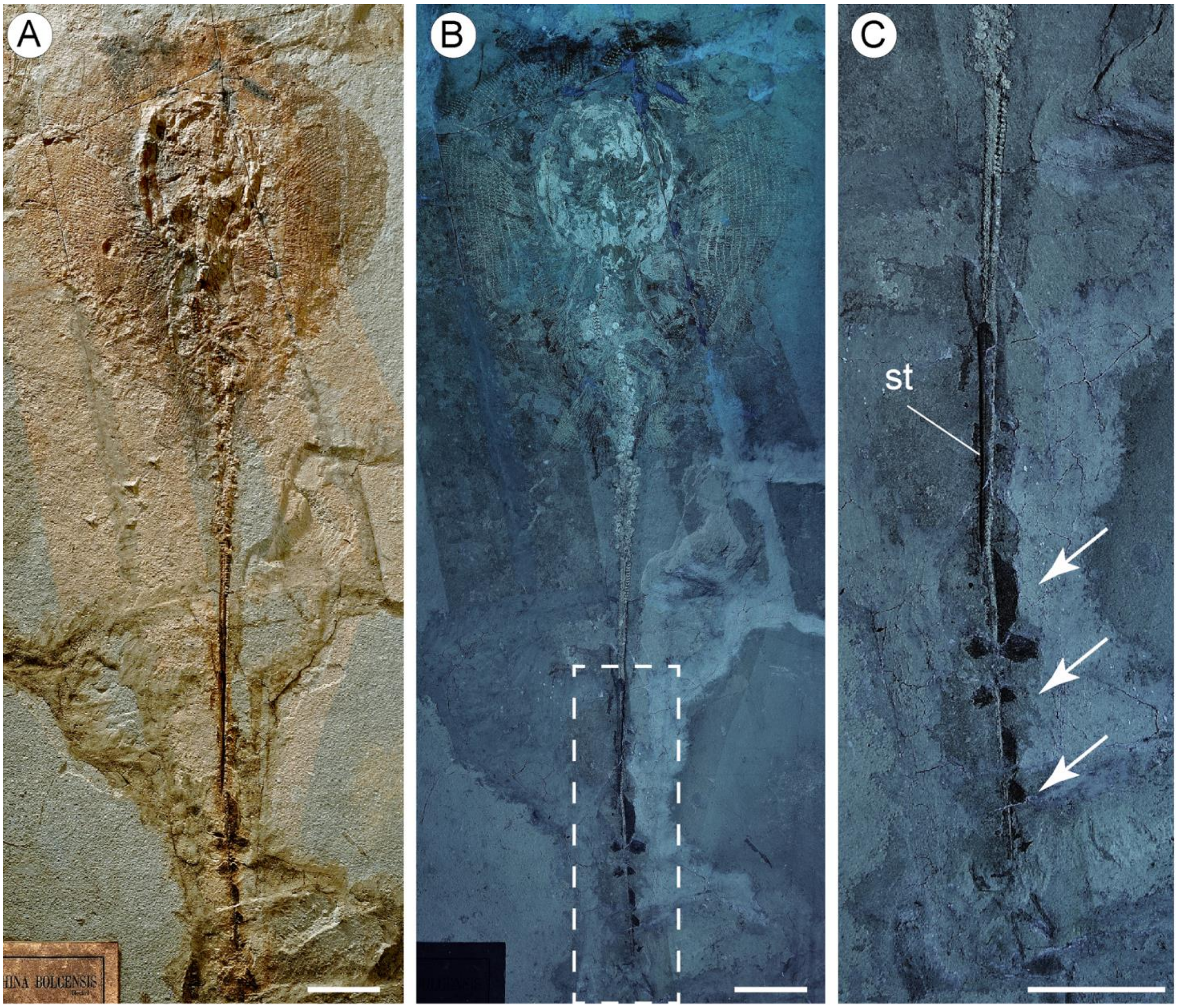

Fig. S6. A-C. *Tethytrygon muricatus* (Volta, 1796) from the Eocene of Bolca Lagerstätte. Specimen MGGC 7456 under A) normal and B) UV light - C. Detail of the terminal portion of the tail showing dark pigmented structures recognized as tail folds (arrows) which fail to reach the tip of tail. Abbreviation: st, caudal sting - Scale bars 50 mm.

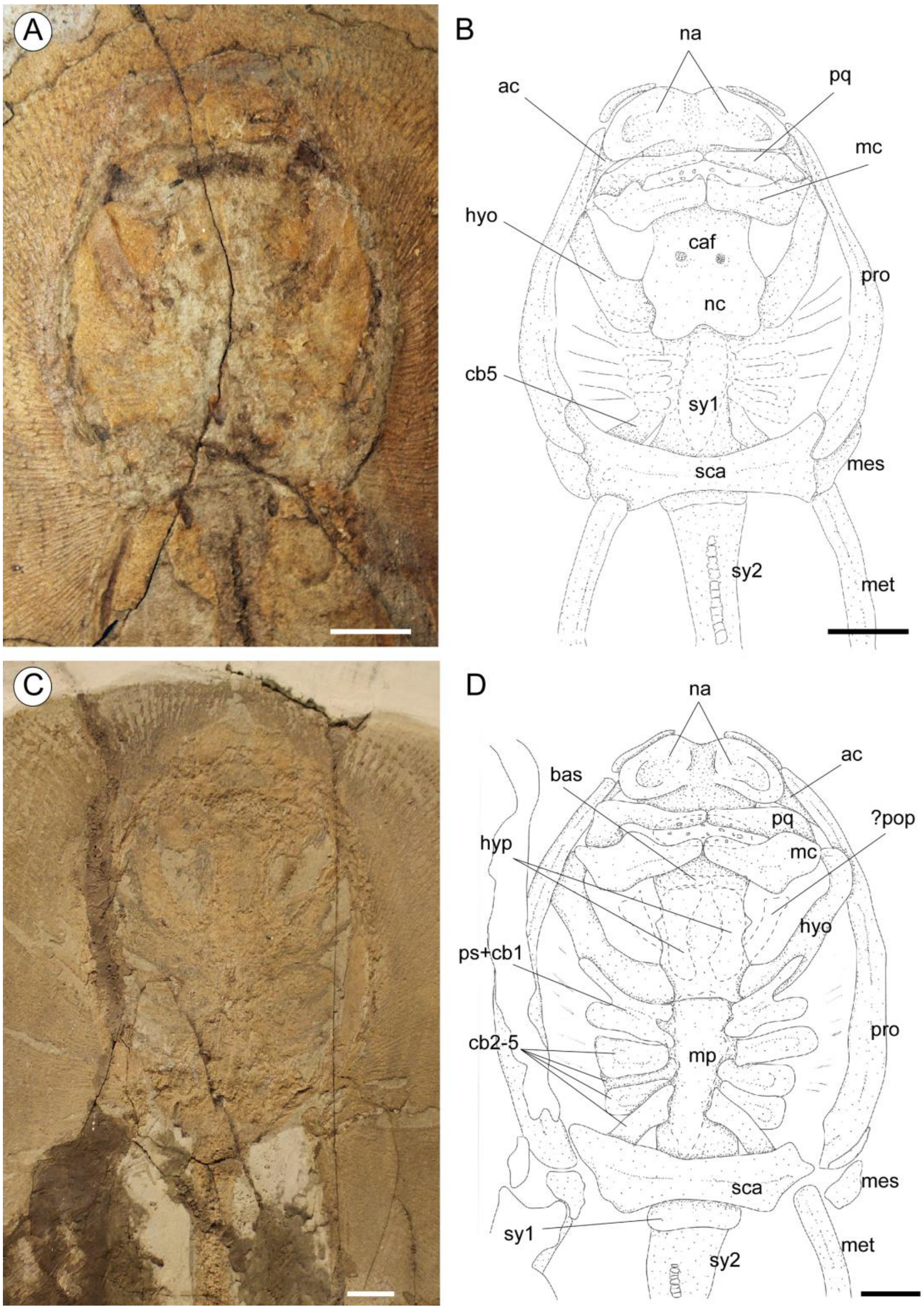

Fig. S7. A-D. Cranial region and pectoral girdle of *Tethytrygon muricatus* (Volta, 1796) from the Eocene of Bolca Lagerstätte - A. MGP-PD 151Z, juvenile female ventrally exposed - B. Reconstruction, pectoral radials omitted - C. MGP-PD 159Z, adult female ventrally exposed - D. Reconstruction, pectoral radials omitted - Scale bars 10 mm. Abbreviations: ac, antorbital cartilage; bas, basihyal; caf, internal carotid artery foramina; cb, ceratobranchials; hyo, hyomandibula; hyp, hypobranchials; mc, Meckel's cartilage; mes, mesopterygium; met, metapterygium; mp, medial plate; na, nasal capsules; nc, neurocranium; pop, postorbital process; pq, palatoquadrate; pro, propterygium; ps, pseudohyoid; sca, scapulocoracoid; sy1, cervicothoracic synarcual; sy2, thoracolumbar synarcual.

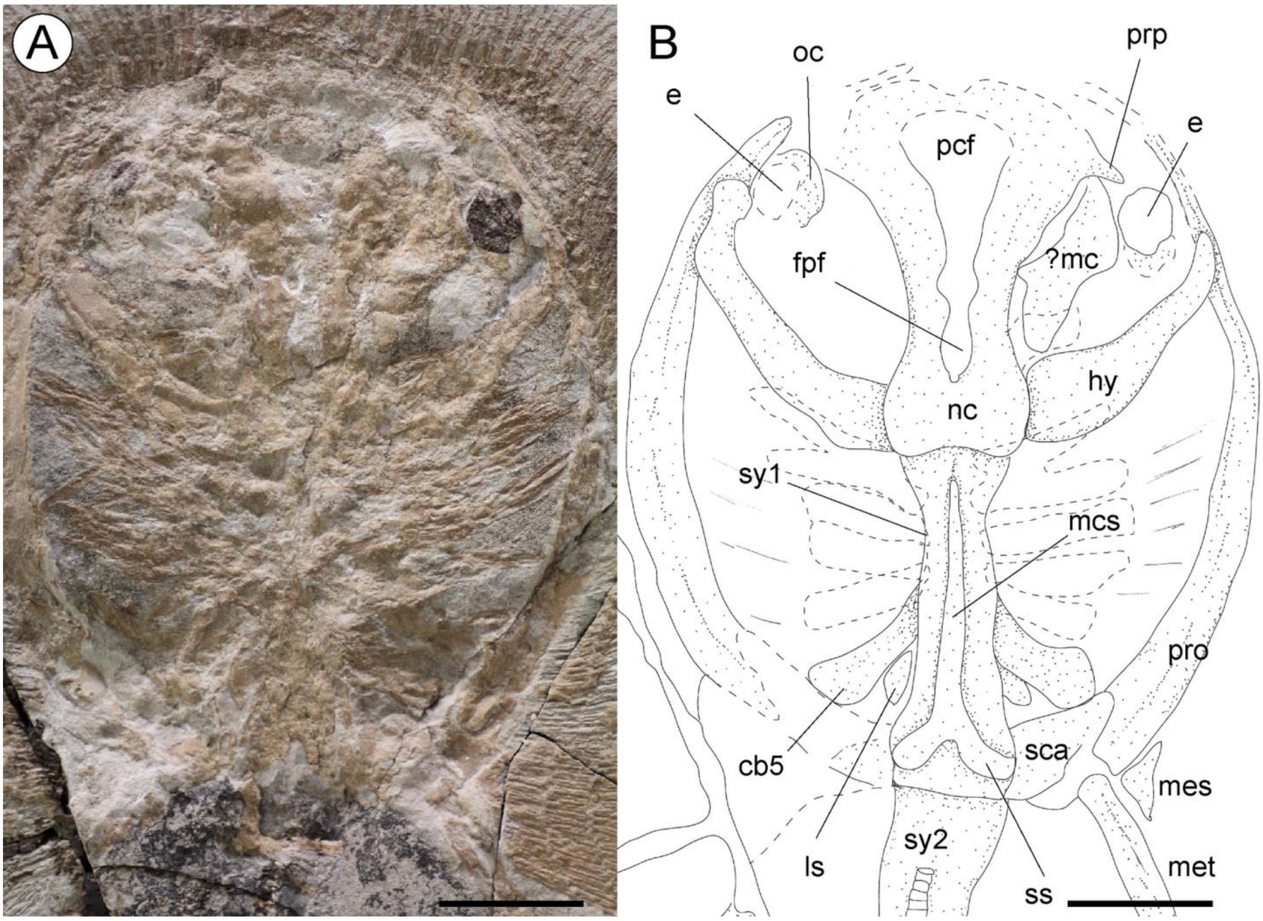

Fig. S8. A-B. Cranial region and pectoral girdle of *Tethytrygon muricatus* (Volta, 1796) from the Eocene of Bolca Lagerstätte - A. MCSNV VII.B.93, adult female dorsally exposed - B. Reconstruction, pectoral radials omitted - Scale bars 50 mm. Abbreviations: cb, ceratobranchials; e, eye; fpf, fronto-parietal fontanelle; hyo, hyomandibula; ls, lateral stay of synarcual; mc, Meckel's cartilage; mcs, median crest of synarcual; mes, mesopterygium; met, metapterygium; nc, neurocranium; oc, optic capsule; pcf, precerebral fontanelle; pro, propterygium; prp, preorbital process; sca, scapulocoracoid; ss, suprascapulae; sy1, cervicothoracic synarcual; sy2, thoracolumbar synarcual.

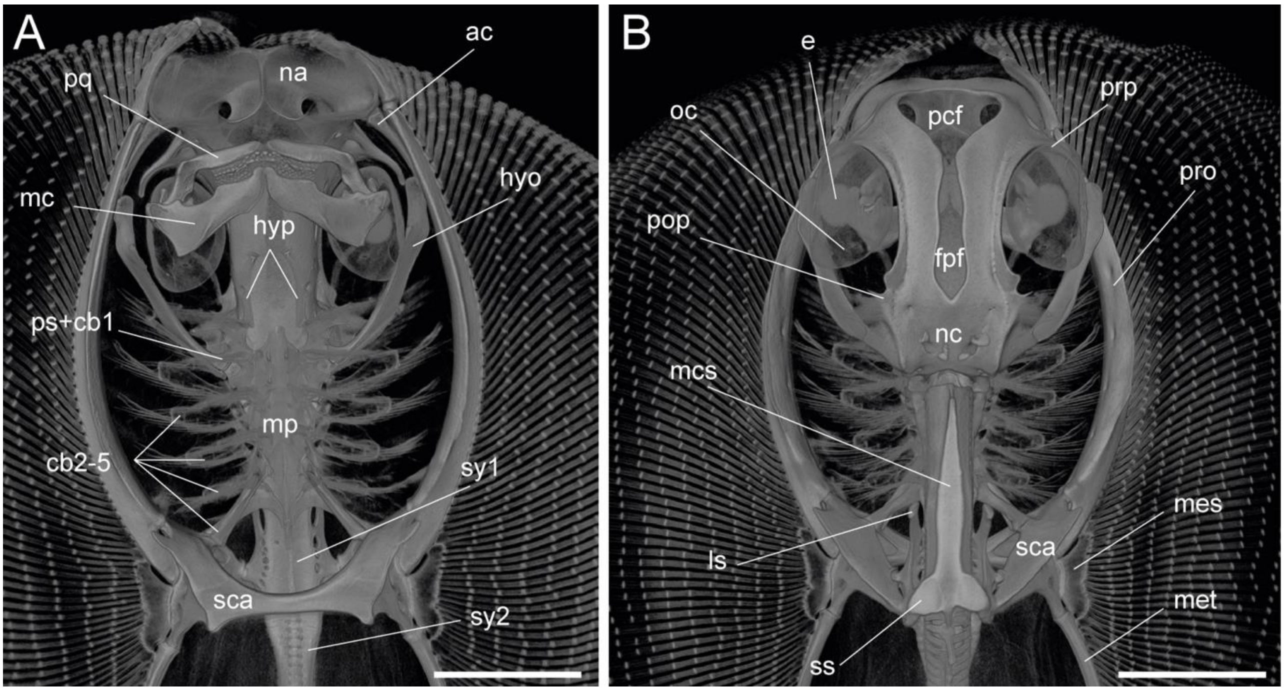

Fig. S9. A-B. Anatomical details of some of a living neotrygonine used for comparisons. A, B. *Taeniura lymma* (Forsskål, 1775) (IUWP uncatalogued specimen), in A) ventral and B) dorsal view - Scale bars 10 mm. Abbreviations: ac, antorbital cartilage; cb, ceratobranchials; e, eye; fpf, fronto-parietal fontanelle; hyo, hyomandibula; hyp, hypobranchials; ls, lateral stay of synarcual; mc, Meckel's cartilage; mcs, median crest of synarcual; mes, mesopterygium; met, metapterygium; mp, medial plate; na, nasal capsules; nc, neurocranium; oc, optic capsule; pcf, precerebral fontanelle; pop, postorbital process; pq, palatoquadrate; pro, propterygium; prp, preorbital process; ps, pseudohyoid; sca, scapulocoracoid; sy1, cervicothoracic synarcual; sy2, thoracolumbar synarcual; ss, suprascapulae.

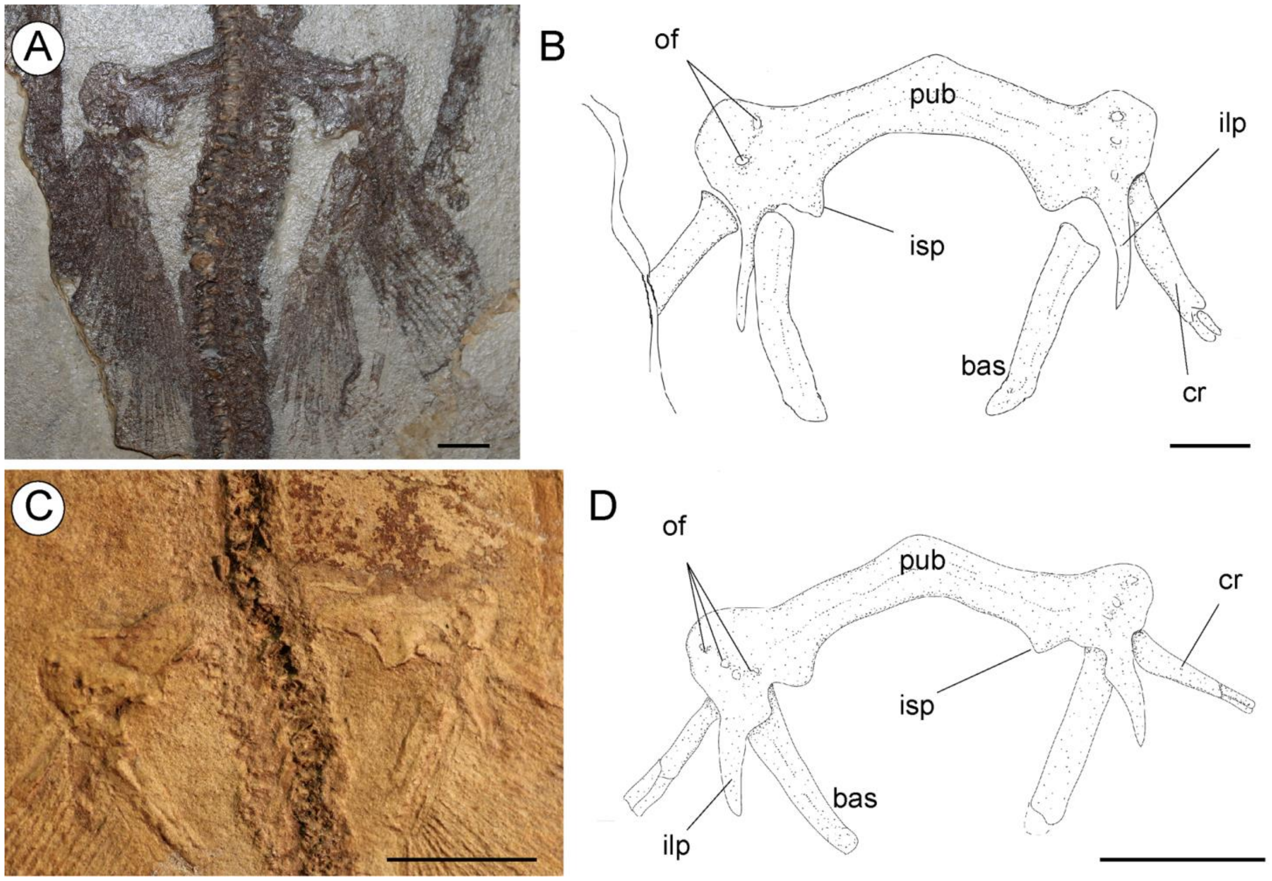

Fig. S10. A-D. Pelvic girdle of *Tethytrygon muricatus* (Volta, 1796) from the Eocene of Bolca Lagerstätte - A. MNHN F.Bol.564, holotype, adult female - B. Reconstruction, pelvic radials omitted - C. MGP-PD 151Z, juvenile female - D. Reconstruction, pelvic radials omitted - Scale bars 10 mm. Abbreviations: bas, basipterygium; cr, compound radial; ilp, iliac process; isp, ischial process; of, obturator foramina; pub, puboischiadic bar.

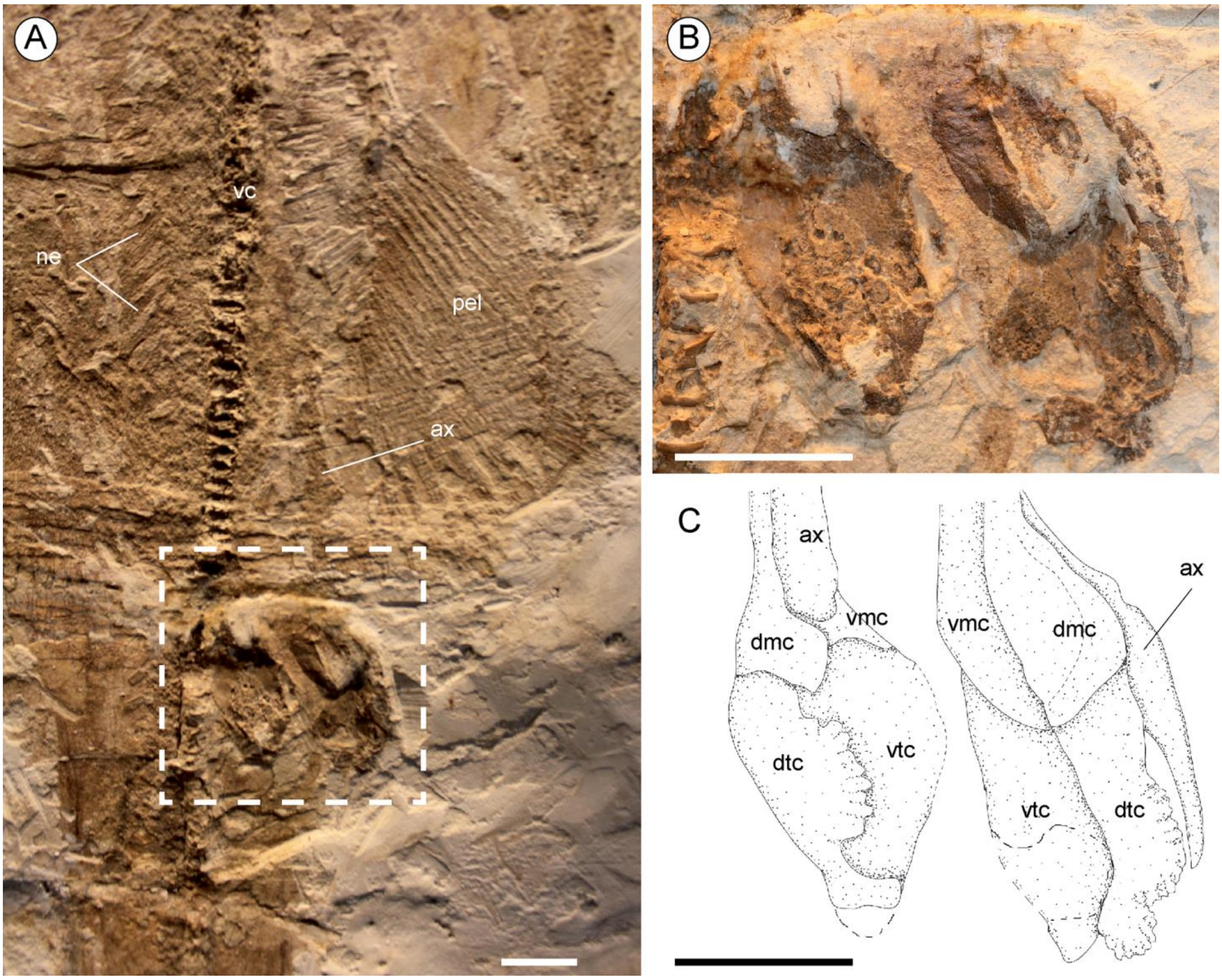

Fig. S11. A-C. *Tethytrygon muricatus* (Volta, 1796) from the Eocene of Bolca Lagerstätte - A. Pelvic region of the adult male MCSNV IG.23194 showing the claspers - B. Close up of the apices - C. Reconstruction - Scale bars 20 mm. Abbreviations: ax, axial cartilage; dmc, dorsal medial cartilage; dtc, dorsal terminal cartilage; ne, neural arches; pel, pelvic fin; vc, vertebral column; vmc, ventral medial cartilage; vtc, ventral terminal cartilage.

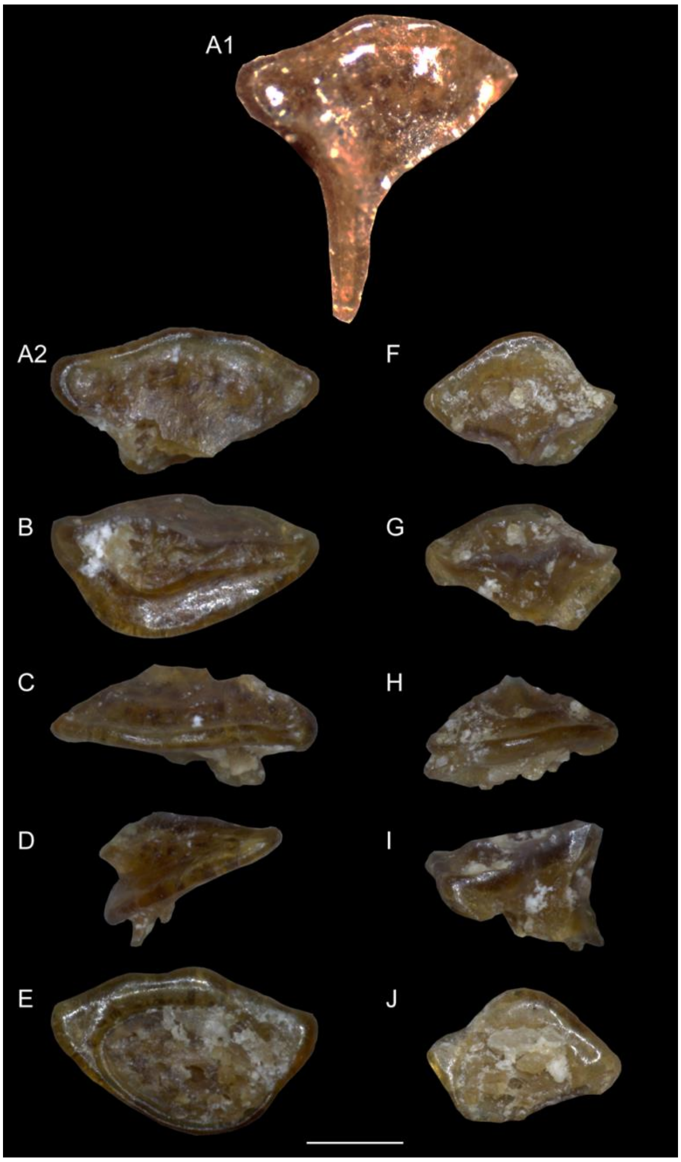

Fig. S12. A-J. *Tethytrygon muricatus* (Volta, 1796) from the Eocene of Bolca Lagerstätte - A-E. A single tooth from the file of 'caniniform' teeth in A) occlusal, B) lingual, C) labial, D) lateral, and E) basal view; the picture depicted in A1 represent the same tooth still in place (the cusp has broken during the extraction) - F-J. Another isolated tooth (not coming from the file of 'caniniform' teeth) in F) occlusal, G) lingual, H) labial, I) lateral, and J) basal view - Scale bar is 500 µm.

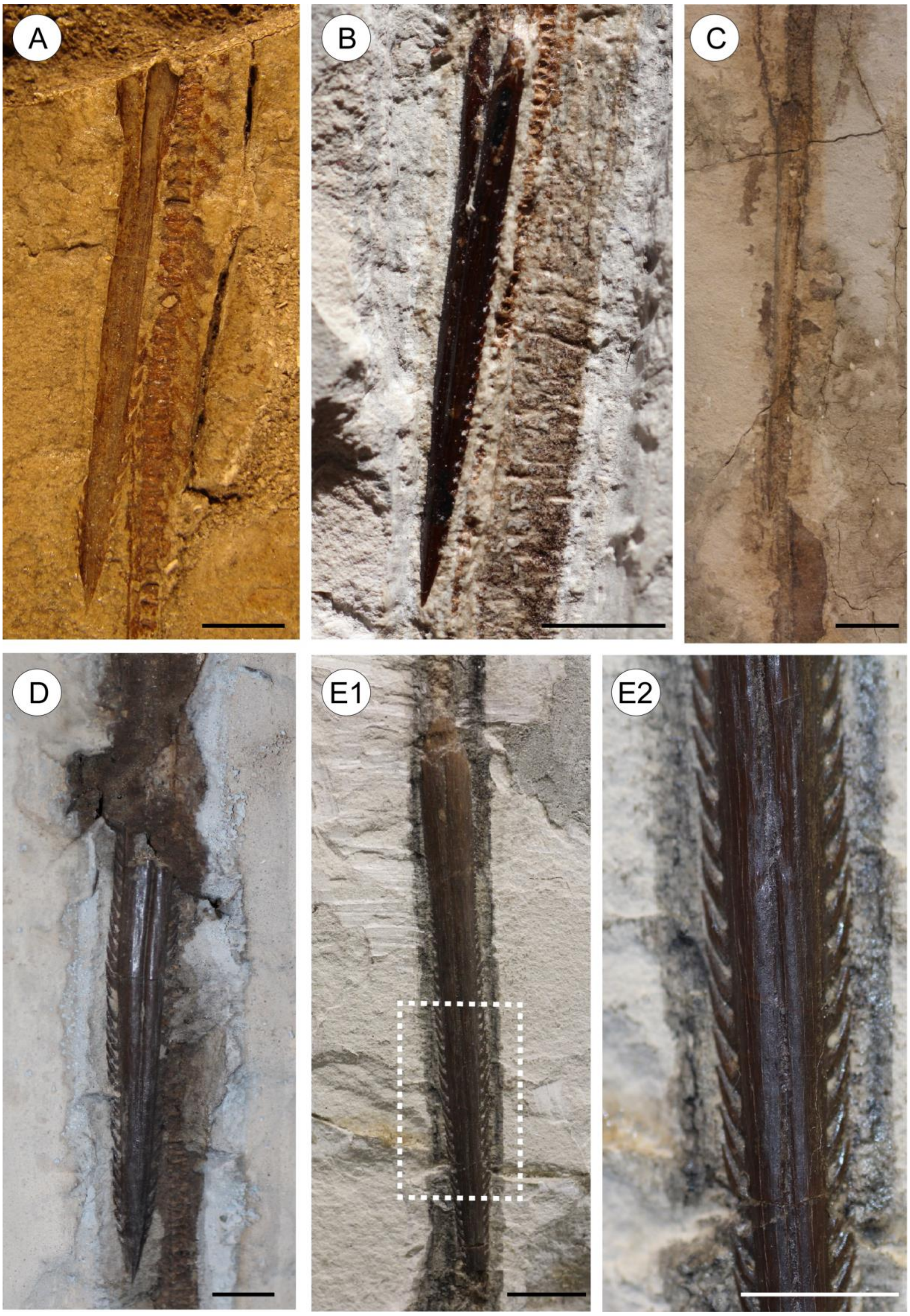

Fig. S13. A-E. *Tethytrygon muricatus* (Volta, 1796) from the Eocene of Bolca Lagerstätte. Serrated caudal stings in A. CMNH 4521 - B. CMC 2 - C. MGGC 7456 - D. MGP-PD 159Z/160Z - E1. MCSNV T.1020 - E2. Close up of the serrations in MCSNV T.1020 - Scale bars 10 mm.

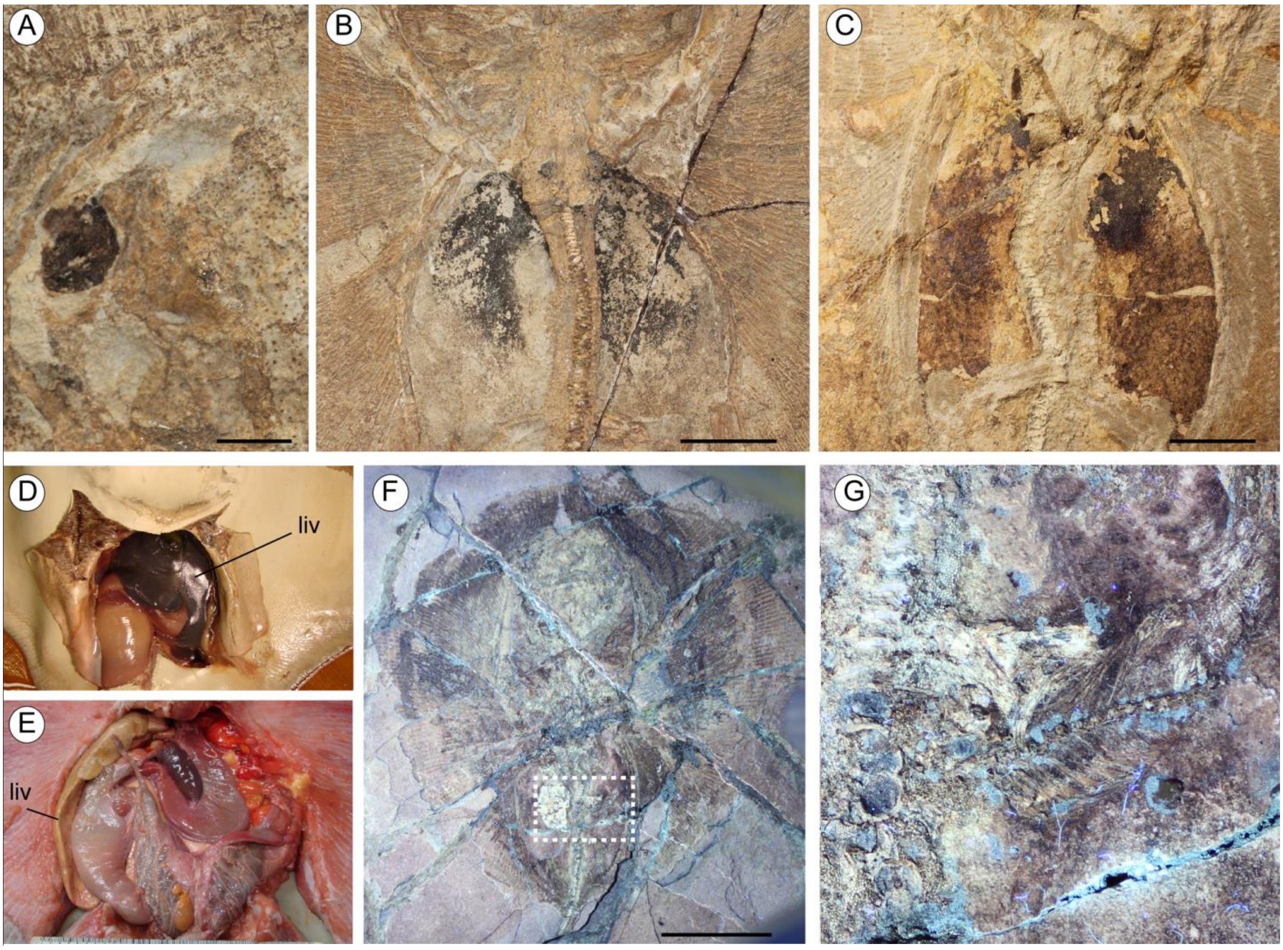

Fig. S14. A-G. *Tethytrygon muricatus* (Volta, 1796) from the Eocene of Bolca Lagerstätte. A. Eye in MCSNV VII.B.92/93 - B. Traces of the liver in MCSNV VII.B.92/93 - C. Traces of the liver in MCSNV IG.186653 - D. Dissected specimen of *Rhinoptera* sp. (IUWP uncatalogued specimen) showing the position of the liver (liv) - E. Dissected specimen of *Potamotrygon tigrina* (IUWP 7361) showing the position of the liver (liv) - F. Specimen MNHN F.Bol.584 under UV light - G. Close up of the abdominal region of MNHN F.Bol.584 showing a partial vertebral column of a bony fish - Scale bars: A, G = 10 mm, B, C = 20 mm, F = 50 mm.

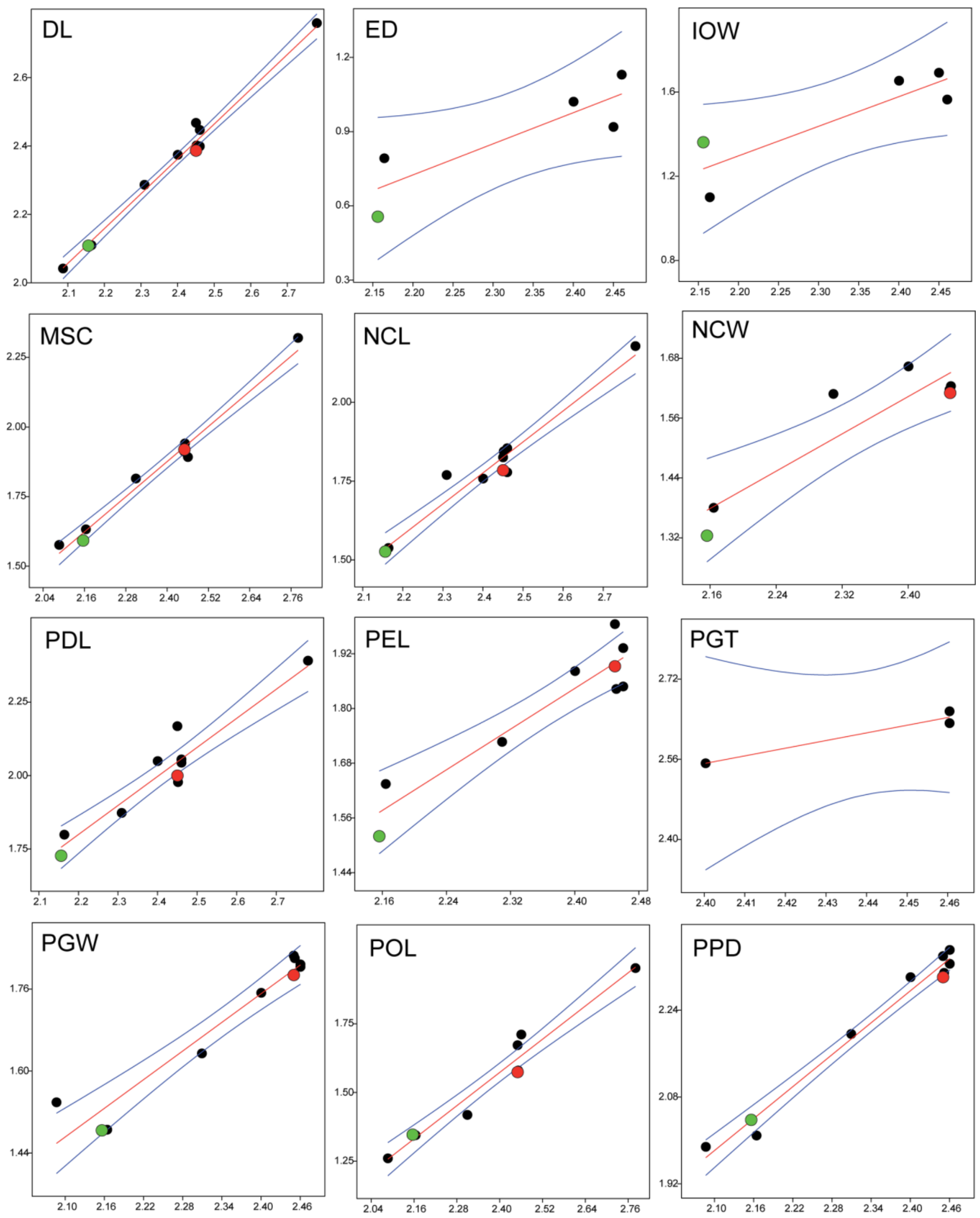

Fig. S15. Scatterplots and regression lines (in red) with 95% confidence bands (in blue) of the relationship between the disc width (on x-axis) and selected morphometric character (on y-axis) *Tethytrygon muricatus* (Volta, 1796). The holotype of *T. muricatus* (MNHN F.Bol564) and the holotype of "*D.*" *zigni* (Molin, 1861) (MGP-PD 150Z/151Z) are marked with red and green circles, respectively. All measurements are log-transformed. Abbreviations: DL, disc length; ED, eye diameter; IOW, interorbital width; MSC, mouth-scapulocoracoid distance; NCL, neurocranial length; NCW, neurocranial width; PDL, distance from tip of disc to max width disc; PEL, pelvic fin length; PGT, pelvics-tip of tail length; PGW, pelvic girdle width (width across pelvic-fin base); POL, preoral length; PPD, prepelvic distance.

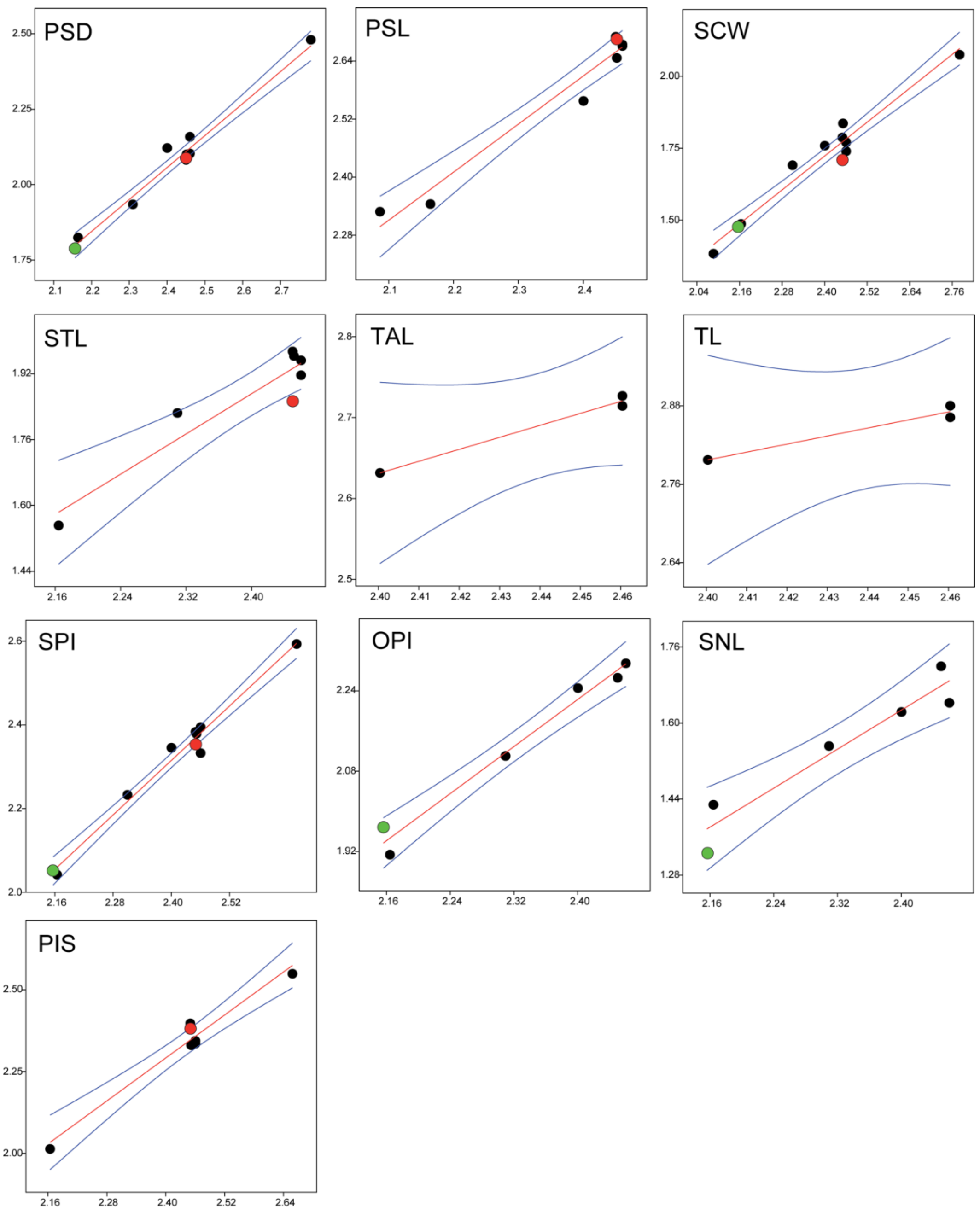

Fig. S16. Scatterplots and regression lines (in red) with 95% confidence bands (in blue) of the relationship between the disc width (on x-axis) and selected morphometric character (on y-axis) *Tethytrygon muricatus* (Volta, 1796). The holotype of *T. muricatus* (MNHN F.Bol564) and the holotype of "*D.*" *zigni* (Molin, 1861) (MGP-PD 150Z/151Z) are marked with red and green circles, respectively. All measurements are log-transformed. Abbreviations: OPI, orbit to pectoral fin insertion; PIS, pectoral-fin insertion to sting length; PSD, prescapular distance (head length); PSL, pre-sting length; SCW, scapulocoracoid width; SNL, snout (preorbital) length; SPI, snout to pectoral fin insertion; STL, sting length; TAL, tail length; TL, total length.

Table S1. Morphological and meristic characters useful to distinguish *Tethytrygon* gen. n. from the living neotrygonines *Taeniura* and *Neotrygon*. All measurements as percentage of disc width (%DW) and mean values are within parentheses. The living species include *Neotrygon annotata, N. australiae, N. caeruleopunctata, N. indica, N. kuhli, N. leylandi, N. ningalooensis, N. orientalis, N. picta, N. trigonoides, N. varidens, Taeniura lymma,* and *T. lessoni*. Data from Schwartz (2005, 2007, 2008), Last & White (2008), Last et al. (2016a, b, c), and Pavan et al. (2018).

Morphometric character	*Tethytrygon*	*Taeniura*	*Neotrygon*
Max disc width (cm)	60.3	37.0	47.0
Max total length (cm)	≈ 150	75.0	70.0
Clasper length	19.0 (19.0)	21.2	20.0 - 23.3
Disc length	88.2 - 95.3 (92.3)	110.5 - 120.4	79.2 - 87.3
Snout to pectoral fin insertion	77.2 - 85.8 (81.9)	92.6 - 106.1	68.1 - 77.2
Orbit to pectoral fin insertion	61.5 - 68.7 (64.7)	63.4 - 74.1	44.5 - 55.9
Snout (preorbital) length	15.1 - 18.5 (16.9)	21.5 - 25.7	13.3 - 18.5
Pectoral-fin insertion to sting	73.9 - 80.1 (79.1)	71.9 - 88.3	32.6 - 44.5
Eye diameter	2.7 - 4.5 (3.7)	6.6 - 8.4	5.1 - 6.6
Inter-eye width	10.7 - 17.7 (14.6)	17.3 - 20.0	12.7 - 18.8
Snout to max disc width	36.3 - 43.5 (40.1)	51.7 - 57.8	36.8 - 41.5
Pelvic fin length	24.5 - 30.0 (27.6)	28.8 - 34.3	13.6 - 22.4
Pelvic girdle width	21.3 - 23.5 (22.7)	17.6 - 24.1	13.7 - 22.1
Preoral length	13.4 - 16.1 (15.0)	17.7 - 20.7	15.9 - 18.8
Prescapular distance (head length)	42.9 - 50.0 (45.8)	49.8 - 57.1	38.2 - 42.3
Sting length	25.3 - 32.8 (29.7)	20.4 - 29.1	13.4 - 19.2
Tail length	170.4 - 184.7 (178.2)	150.0 - 170.0	110.0 - 150.0
Total length	249.5 - 263.0 (255.0)	232.5 - 265.6	159.1 - 224.6
Mouth-scapulocoracoid distance	28.1 - 31.5 (30.0)	?	?
Neurocranial length	22.5 - 24.9 (23.9)	?	?
Neurocranial width	14.7 - 18.3 (16.2)	?	?
Pelvics to tip of tail length	141.8 - 156.7 (149.0)	?	?
Prepelvic distance	71.7 - 78.2 (75.4)	?	?
Presting length	152.6 - 173.4 (162.3)	?	?
Scapulocoracoid width	19.7 - 22.8 (21.1)	?	?

Meristic and body characters	*Tethytrygon*	*Taeniura*	*Neotrygon*
Propterygial radials	49 - 53 (51)	47 - 50	40 - 51
Mesopterygial radials	16 - 20 (18)	15 - 18	12 - 17
Metapterygial radials	40 - 45 (43)	47 - 50	44 - 50
Total pectoral radials	108 - 117 (112)	110 - 115	101 - 113
Pelvic radials	24 - 27 (25)	18 - 25	19 - 24
Monospondylous trunk vertebrae (excl. synarcual)	23 - 26 (24)	37 - 39	34 - 46
Diplospondylous vertebrae (anterior to sting)	100 - 109 (105)	90 - 101	57 - 67
Diplospondylous vertebrae (posterior to sting)	45 - 54 (48)	40 - 55	14 - 40

Total vertebrae	175 - 179 (177)	175 - 184	109 - 145
Number of stings	1 (1)	1 - 2	1 - 2
Sting serrations (total)	48 - 90 (69)	59 - 69	?
Tooth ornamentation	absent	present	absent
Denticles	absent/present	absent/present	absent/present
Thorns	absent	present	absent/present

Table S2. Relationships between disc width (log x) and the various morphometric characters (log y) using least squares regression for *Tethytrygon muricatus* (Volta, 1796) from the Eocene of Bolca Lagerstätte. All $p < 0.05$, except variables indicated with asterisk ($p > 0.05$).

Variable character log(y)	Slope (m)	Intercept (b)	Coefficient of determination (r^2)	95% CI on m	95% CI on b
Disc length	1.04 ± 0.04	-0.13 ± 0.10	0.99	0.97 1.10	-0.27 0.02
Eye diameter	1.28 ± 0.41	-2.04 ± 0.96	0.76	0.61 2.15	-4.23 -0.53
Interorbital width	1.41 ± 0.44	-1.80 ± 1.03	0.77	0.64 4.30	-8.82 0.09
Mouth-scapulocoracoid distance	1.07 ± 0.05	-0.69 ± 0.13	0.98	0.99 1.23	-1.07 -0.48
Neurocranial length	1.00 ± 0.08	-0.61 ± 0.19	0.95	0.91 1.29	-1.32 -0.39
Neurocranial width	0.94 ± 0.18	-0.64 ± 0.43	0.84	0.67 1.89	-3.00 -0.05
Orbit to pectoral fin insertion	1.17 ± 0.09	-0.59 ± 0.20	0.98	1.00 1.35	-1.00 -0.19
Distance from tip of disc to max width disc	1.02 ± 0.11	-0.44 ± 0.27	0.91	0.84 1.23	-0.93 -0.02
Pelvic fin length	1.12 ± 0.16	-0.83 ± 0.38	0.88	0.81 1.70	-2.23 -0.13
Pelvics-tip of tail length *	1.53 ± 0.34	-1.12 ± 0.83	0.95	1.33 3.06	-1.59 -0.65
Pelvic girdle width (width across pelvic-fin base) *	0.93 ± 0.09	-0.49 ± 0.21	0.93	0.70 1.14	-0.98 0.06
Pectoral-fin insertion to sting	1.10 ± 0.11	-0.34 ± 0.26	0.96	0.94 6.99	-14.81 0.02
Preoral length	1.01 ± 0.08	-0.84 ± 0.19	0.96	0.84 1.17	-1.21 -0.47
Prepelvic distance	0.98 ± 0.05	-0.08 ± 0.12	0.98	0.86 1.07	-0.30 0.20
Prescapular distance (head length)	1.08 ± 0.06	-0.54 ± 0.15	0.98	1.00 1.20	-0.82 -0.33
Pre-sting length	1.00 ± 0.08	0.22 ± 0.20	0.96	-0.10 1.34	-0.60 2.89
Scapulocoracoid width	0.97 ± 0.07	-0.61 ± 0.16	0.96	0.84 1.11	-0.95 -0.32
Snout (preorbital) length	1.03 ± 0.15	-0.83 ± 0.35	0.92	0.74 1.33	-1.56 -0.18
Snout to pectoral fin insertion	1.08 ± 0.05	-0.29 ± 0.13	0.98	1.01 1.23	-0.65 -0.12
Sting length	1.22 ± 0.20	-1.06 ± 0.47	0.89	-0.73 4.41	-8.91 3.73
Tail length *	1.48 ± 0.18	-0.92 ± 0.44	0.99	1.38 3.00	-1.17 -0.67
Total length *	1.23 ± 0.26	-0.16 ± 0.63	0.96	1.08 2.47	-0.52 0.20

CHARACTER LIST

Here below is the list of morphological characters used for the phylogenetic analysis, based on Marramà et al. (2018a). Data are taken from Claeson et al. (2010) (from 1 to 65), Aschliman et al. (2012a) (from 66 to 88), Underwood et al. (2017) (from 89 to 97). Characters 98 to 102 are based on Marramà et al. (2018a), which in turn are based on Herman et al. (1998, 1999, 2000), Schaefer & Summers (2005), Lim et al. (2015), Last et al. (2016a, b). Character 103 has been newly added. Characters for all genera are coded following Claeson et al. (2010), except *Aetomylaeus*, *Pastinachus* and *Neotrygon* which are not present in Claeson et al. (2010) and are therefore coded following Carvalho et al. (2016) and Underwood et al. (2017). Some characters for some genera (indicated with *) have been also recoded based on new observation of the comparative material, and following previous or more recent literature (see also **Remarks** below).

Characters 1 to 65 are from Claeson et al. (2010), which in turn are based on those of Carvalho *et* al. (2004) (CMG). Dental character information for *Aetomylaeus, Taeniura*, *Neotrygon*, and *Pastinachus* is taken from Herman et al. (1998, 1999, 2000).

1. (01 of CMG) Tubules of subpleural components of hyomandibular lateral line canals: (0) not branched at extremities; (1) extremities dichotomously branched
2. (02 of CMG) Subpleural components of the hyomandibular lateral line canals: (0) posterior branch extends caudally more or less parallel to longitudinal body axis; (1) posterior branch inflects towards midline to form a lateral hook; (2) posterior branch inflects to continue anteriorly almost parallel to anterior branch, forming a large indentation
3. (03 of CMG) Suborbital components of infraorbital lateral line canals: (0) projecting posteriorly lateral to mouth; (1) projecting posteriorly lateral to mouth and anteriorly lateral to nasal openings; (2) forming a complex web−like pattern on lateral aspects of the anteroventral disc region
4. (04 of CMG) Scapular loops formed by scapular components of trunk lateral line canals: (0) absence of loops; (1) presence of scapular loops
5. (05 of CMG) Anterior process of neurocranium: (0) absent; (1) present
6. (06 of CMG) Preorbital process: (0) present; (1) absent
7. (07 of CMG) Preorbital canal for passage of superficial ophthalmic nerve: (0) dorsally located; (1) anteriorly located
8. (08 of CMG) Foramen for the optic (II) nerve: (0) moderately sized; (1) very enlarged

9. (09 of CMG) Postorbital process of neurocranium: (0) infraorbital lateral line canal separates postorbital process from small, anterior triangular outgrowth (supraorbital process) of the supraorbital crest; (1) postorbital process with small foramen for passage of infraorbital lateral line canal
10. (10 of CMG) Extent of orbital region: (0) orbital region of neurocranium long; (1) shortened orbital region with more anteriorly placed supraorbital and postorbital process
11. (11 of CMG) Postorbital process: (0) without ventrolateral projection; (1) continuing ventrolaterally to form a cylindrical projection
12. (12 of CMG) Ventrolateral expansion of nasal capsules: (0) nasal capsules laterally expanded; (1) nasal capsules ventrolaterally expanded
13. (13 of CMG) Articulation between hyomandibula and Meckel's cartilage: (0) hyomandibulae directly attached to lower jaws; (1) hyomandibulae articulating with lower jaws through strong, stout ligament (hyomandibular–Meckelian ligament) at distal tip
14. (14 of CMG) Angular cartilages: (0) absence of angular cartilages within hyomandibular–Meckelian ligament; (1) presence of angular cartilages within ligament
15. (15 of CMG) Secondary hyomandibular cartilages: (0) absent; (1) present
16. (16 of CMG) Symphysial fusion of upper and lower jaws: (0) antimeres separate at symphysis; (1) both antimeres of jaws symphysially fused
17. (17 of CMG) Mandibular width at symphysis: (0) lower jaws slender at symphysis; (1) lower jaws symphysially thickened
18. (18 of CMG) Lateral projections of lower jaws: (0) absent; (1) present
19. (20 of CMG) Basihyal cartilage: (0) basihyal laterally elongated, fused to first hypobranchialis; (1) basihyal a single element, but separate from first hypobranchials; (2) basihyal separate from first hypobranchials but fragmented into more than one component; (3) basihyal absent
20. (21 of CMG) Fusion of ventral pseudohyoid and first ceratobranchial: (0) absent; (1) present
21. (22 of CMG) Arrangement of posterior ceratobranchials: (0) separate from each other; (1) ankylosis between fourth and fifth ceratobranchials; (2) fourth and fifth ceratobranchials fused to each other.
22. (23 of CMG) Median projection of the basibranchial medial plate: (0) absent; (1) present
23. (24 of CMG) Articulation between fifth epi– and ceratobranchial elements to scapulocoracoid: (0) close together; (1) widely separated
24. (25 of CMG) Lateral stay of synarcual: (0) originates ventral to spinal nerve foramina; (1) originates dorsal to spinal nerve foramina; (2) contacting synarcual both dorsally and ventrally to foramina

25. (26 of CMG) Fossa on dorsal scapular region: (0) absent; (1) present
26. (27 of CMG, modified) Contact between pro- and mesopterygium in the pectoral fin: (0) present; (1) absent. **Remarks**: this character was originally described by Lovejoy (1996) who recognized the absence of articulation between pro- and mesopterygium as a derived state in *Potamotrygon* and *Plesiotrygon*, unique among myliobatiforms. Subsequent phylogenetic analyses (Carvalho et al., 2004; Claeson et al., 2010) have considered the presence of this articulation as derived state (1) but coding incorrectly the state for all taxa considered. For this character we therefore followed the original description of Lovejoy (1996) and coded the derived state (absence) only for *Potamotrygon* and *Plesiotrygon*. *Tethytrygon* shows the basalmost condition (0).
27. (28 of CMG) Distinct components of the mesopterygium: (0) mesopterygium single element; (1) fragmented; (2) missing altogether
28. (29 of CMG) Lateral expansion of radials in pectoral region: (0) absent; (1) present
29. (30 of CMG) External margin of mesopterygium: (0) more or less straight, not fused to radials; (1) undulated, not fused to radials; (2) highly sinuous, appearing to be fused with articulating radial elements
30. (31 of CMG) Median prepelvic process: (0) absent or weakly developed; (1) very elongated
31. (32 of CMG) Pelvic girdle shape: (0) not arched or only moderately so; (1) greatly arched
32. (33 of CMG) Dorsal fin: (0) present; (1) absent
33. (34 of CMG) Cartilaginous rod in tail: (0) absent; (1) present
34. (35 of CMG) Caudal fin: (0) present; (1) reduced to tail−folds; (2) absent
35. (36 of CMG) Adductor mandibulae complex: (0) without posteromedial extension; (1) posteromedial extension present
36. (37 of CMG) Spiracularis muscle: (0) projecting ventrally to insert on either palatoquadrate, Meckel’s cartilage, and or hyomandibula; (1) projecting ventrally and posteriorly beyond hyomandibulae and both sets of jaws to insert dorsal to coracomandibularis; (2) projecting ventrally and posteriorly beyond hyomandibulae and both sets of jaws to insert ventral to coracomandibularis
37. (38 of CMG) Depressor mandibularis muscle: (0) present; (1) absent
38. (39 of CMG) Coracohyoideus muscle: (0) not connected at midline; (1) connected at midline
39. (40, CMG) Urea retention: (0) urea retained in blood; (1) urea excreted in urine
40. (41 of CMG) Rectal gland: (0) present; (1) reduced
41. (42 of CMG) Spiracular tentacle: (0) absent; (1) present

42. (43 of CMG) Cephalic lobes: (0) absent; (1) single and continuous; (2) single with an indentation; (3) paired
43. (44 of CMG) Nasal curtain: (0) not reaching mouth region; (1) extending posteriorly as far as mouth opening
44. (modified from 19 of CMG) Tooth type in both upper and lower jaws: (0) minute; (1) broad
45. (modified from 19 of CMG) Arrangement of teeth in both upper and lower jaws: (0) arranged in separate diagonal rows or ribbons; (1) horizontal conveyor or pavement−like arrangement
46. (modified from 19 of CMG) Tooth shape: (0) square to rounded; (1) hexagonal, six distinct sides; (2) rectangular with posteriorly deflected lateral margins. **Remarks**: although *Pastinachus* has some teeth hexagonal or quincuncial in shape (Herman et al., 1998; Adnet et al., 2018), these are clearly derived from the basal condition (0) which is still retained in some teeth. Therefore, the hexagonal teeth of *Pastinachus* are not homologous to those of most of myliobatids.
47. Lateral teeth: (0) present; (1) absent
48. Differentiation of median teeth from lateral teeth: (0) median and lateral teeth are similar; (1) median teeth relatively expanded
49. Differentiation among lateral teeth: (0) lateral teeth unexpanded; (1) some lateral teeth expanded
50. Relative amount of curvature in expanded lower teeth: (0) straight and uncurved; (1) moderately curved; (2) strongly curved
51. Upper tooth curvature: (0) uncurved; (1) curved
52. Direction of tooth curvature: (0) concave; (1) flat/horizontal; (2) convex
53. Tooth association: (0) loosely interlocking; (1) sometimes loosely interlocking or tightly interlocking; (2) tightly interlocking
54. Tooth Interlocking mechanism: (0) overlapping; (1) tongue and groove; (2) no direct contact
55. Shape of interlocking tongue: (0) bulbous; (1) short shelf; (2) long shelf. **Remarks**: this character has been recoded has unknown for *Protohimantura*, due to an erroneous coding in the previous analysis (Marramà et al., 2018a).
56. Crown height: (0) high, the crown height exceeds root depth on unworn teeth; (1) low crown
57. Occlusal surface: (0) cusped; (1) smooth; (2) depressed
58. Crown shape in anterior or posterior view: (0) straight; (1) domed; (2) deep
59. Lateral margins: (0) not pinched; (1) pinched
60. Root type: (0) holaulacorhizous; (1) polyaulacorhizous
61. Number of roots: (0) 2 roots; (1) more than 2 roots. **Remarks**: we only included two states instead of the original three of Claeson et al. (2010) since their state (1) (3 to 4 roots) is only

characteristic of *Brachyrhizodus*, which is not included here. States are therefore re-coded consequently. *Tethytrygon* show the basal condition of myliobatiforms with two roots (state 0).

62. Roots in basal view: (0) triangles; (1) narrow blocks; (2) fine edges. **Remarks:** we only included three states instead of the original four of Claeson et al. (2010) since the state (1) of Claeson et al. (2010) (wide blocks) is only characteristic of extinct myliobatids, which are not included here. States are therefore re-coded consequently. *Tethytrygon* gen. n. shows the basal condition of myliobatiforms with root triangular in basal view (state 0).
63. Distance between roots: (0) narrower than root laminae; (1) broad, groove wider than root laminae. **Remarks:** the polarity of the states follows here Claeson et al. (2010) at pag. 666, contrary to Claeson et al. (2010) at pag. 674, and consequently Marramà et al. (2018a), in which the polarity was accidentally inverted.
64. Inclination of roots: (0) no inclination; (1) offset and step–like; (2) long and strongly inclined
65. Root groove position: (0) regularly spaced between laminae; (1) irregularly spaced between laminae

Characters 66 to 88 are selected characters from Aschliman et al. (2012a) (ASC) which are useful to better define the relationships within the Myliobatiformes and that were not included in the analysis of Carvalho et al. (2004) and Claeson et al. (2010). Amongst the 89 characters of Aschliman et al. (2012a) only 23 characters were conserved herein since the others are uninformative for myliobatiforms and outgroups selected.

66. (9 of ASC) Levator and depressor rostri muscles: (0) absent; (1) present
67. (14 of ASC) Serrated tail stings: (0) absent; (1) present.
68. (15 of ASC, modified) Placoid scales: (0) uniformly present; (1) limited; (2) absent. **Remarks**: Aschliman et al. (2012a) considered myliobatiforms as largely to totally free of denticles over the entire body surface and, consequently, coded this character as (2) for all myliobatiforms. However, *Raja* and most of the stingrays (*Himantura, Plesiobatis, Pastinachus, Paratrygon, Styracura, Plesiotrygon, Potamotrygon,* and *Pteroplatytrygon*) actually still retain dermal denticles, although they are reduced and not uniformly present (1) (see Last et al., 2016a). Fossil stingrays *Heliobatis* and *Asterotrygon* (see Carvalho et al., 2004) and *Protohimantura* are coded (1) as well. On the contrary, *Hexatrygon, Urolophus, Trygonoptera, Myliobatis, Aetobatus, Rhinoptera* are totally free of dermal denticles (Last et al., 2016) and were coded (2). Depending on the species and ontogenetic stage, the genera

Urobatis, Urotrygon, Taeniura, Neotrygon, Tethytrygon, Dasyatis, Gymura, Aetomylaeus,* and *Mobula* can show both the derived states (1 and 2) (Last et al., 2016a). The limited (1) and absence (2) of dermal denticles are considered derived states.

69. (16 of ASC, modified) Thorns: (0) present; (1) absent. **Remarks**: Although Aschliman et al. (2012a) considered the presence of thorns only in Dasyatidae among myliobatiforms, we recoded and modified this character based on the most recent descriptions of Last et al. (2016a). Since *Rhinobatos* and *Raja* are our outgroups we considered the presence of thorns as the basal condition (0). The absence of thorns (1) can be considered as derived condition in *Hexatrygon, Plesiobatis, Urolophus, Trygonoptera, Pastinachus, Gymnura* and all pelagic stingrays (except *Aetomylaeus*) (Last et al., 2016a). The states for *Neotrygon** (0&1) and the fossil *Heliobatis** and *Asterotrygon** (0) have been recoded, following Carvalho et al. (2004), and Last et al. (2016a). Thorns are absent in *Tethytrygon* (1).
70. (18 of ASC) Pulp cavities in tooth roots: (0) large; (1) broad and elongated; (2) small; (3) absent
71. (19 of ASC, modified) Tooth vascularization: (0) orthodont; (1) osteodont; (2) modified osteodont. **Remarks**: Aschliman et al. (2012a) reported the presence of osteodentine only in rajids, although osteodentine was not observed in *Raja* (Herman et al., 1995, 1996). Moreover, Herman et al. (2000) considered of osteodont type (1) teeth of *Plesiotrygon, Potamotrygon, Taeniura, Pteroplatytrygon, Dasyatis* and *Manta*. A distinct type of osteodentine (modified) was observed only in *Aetobatus*, *Aetomylaeus*, *Myliobatis, Rhinoptera* and *Pastinachus* (2). The tooth vascularization is unknown in *Tethytrygon*. and orthodont in *Neotrygon** according to Herman et al. (1998).
72. (21 of ASC) Infraorbital loop of suborbital and infraorbital canals: (0) absent; (1) present and forming a simple posterolaterally directed loop; (2) present and forming a complex reticular pattern or a number of loops; (3) the loop is directed to the anterior
73. (26 of ASC, modified) Rostral cartilage: (0) complete; (1) vestigial or absent. **Remarks**: we only include two states instead of the original three of ASC since the state 1 of ASC (rostral cartilage fails to reach the tip of the snout) was only coded for the batoids *Platyrhina* and *Platyrhinoidis*, not considered in our analysis. The rostral cartilage is vestigial or absent in all myliobatiforms, including *Tethytrygon.*
74. (36 of ASC, modified) Postorbital process: (0) narrow; (1) very broad and shelf-like. **Remarks**: we only include two states instead of the original three of ASC since the state 1 of ASC (absent) is only characteristic of torpediniforms, which are not included here. *Tethytrygon* shows a broad and shelf-like postorbital process (1).

75. (39 of ASC, modified) Jugal arch: (0) present; (1) absent. **Remarks:** our states are inverted with respect those of Aschliman et al. (2012a) since the presence of this character is basal in outgroups that we considered, whereas its absence is a derived condition in myliobatiforms. The character is unknown in *Tethytrygon*.
76. (48 of ASC) Basihyal and first hypobranchial: (0) both present and unsegmented; (1) basihyal is segmented; (2) basihyal is absent; (3) basihyal and first hypobranchial cartilages absent
77. (50 of ASC) Suprascapulae: (0) articulates with vertebral column; (1) fused medially to synarcual (= pectoral arch); (2) fused medially and laterally to synarcual. **Remarks:** we only include three states instead of the original four of ASC since the state (0) of ASC (free of vertebral column) is only characteristic of torpediniforms, which are not included here. States are therefore re-coded consequently. *Tethytrygon* has suprascapulae which are fused medially and laterally to synarcual as in all myliobatiforms (2).
78. (53 of ASC) Ball and socket articulation between scapular process and synarcual: (0) absent; (1) present.
79. (54 of ASC) Second (thoracolumbar) synarcual: (0) absent; (1) present.
80. (55 of ASC) Ribs: (0) present; (1) absent. **Remarks**: The absence of ribs has been suggested to be a synapomorphic character of stingrays of the order Myliobatiformes (see e.g., Carvalho et al., 2004; Aschliman et al., 2012a) and are also absent in *Tethytrygon*. However, our examination of the comparative material and the radiographs in the available literature, detected their absence also in skates (see Marramà et al., 2018d) including *Raja**.
81. (63 of ASC) Segmentation of propterygium: (0) posterior to mouth, (1) proximal segment of propterygium of pectoral girdle is between mouth and antorbital cartilage; (2) the first segment is adjacent to the nasal capsule; (3) the first segment is adjacent to anterior margin of antorbital cartilage or anterior to margin of nasal capsule
82. (74 of ASC) Pseudosiphon: (0) present; (1) absent
83. (75 of ASC) Dorsal marginal clasper cartilage: (0) lacks medial flange; (1) possesses medial flange
84. (76 of ASC) Dorsal terminal cartilage: (0) smooth margin; (1) crenate margin
85. (77 of ASC) Cartilage forming component claw: (0) present; (1) absent; (2) cartilage embedded in integument and is not visible externally; (3) cartilage lines the inner ventral margin of the clasper glans and often forms the component shield
86. (78 of ASC) Ventral terminal cartilage (accessory terminal 1 cartilage in rajids): (0) simple; (1) free distally and forms component sentinel or is fused with ventral marginal cartilage and forms component projection; (2) folded ventrally along its long axis to form a convex flange

87. (79 of ASC) Ventral terminal cartilage (accessory terminal 1 cartilage in rajids): (0) attached over length to axial cartilage; (1) free of axial cartilage

88. (85 of ASC, modified) Spiracularis: 0 = undivided; (1) splits into lateral and medial bundles, with the medial bundle inserting onto the posterior surface of Meckel's cartilage and the lateral bundle inserting onto the dorsal edge of the hyomandibula; (2) extends beyond the hyomandibula and Meckel's cartilage; (3) subdivided proximally and inserts separately onto the palatoquadrate and the hyomandibula. **Remarks:** we only include four states instead of the original five of ASC since the state 1 of ASC (spiracularis divided and one bundle enters the dorsal oral membrane underlying the neurocranium) is only characteristic of torpediniforms, which are not included here.

Characters 89 to 97 are selected from Underwood et al. (2017) (UND). Amongst the 77 characters of Underwood et al. (2017) only nine characters were conserved herein since the others are the same of Carvalho et al. (2004) and/or Claeson et al. (2010) or autapomorphic for single genera.

89. (52 of UND, modified) Sexual heterodonty: (0) absent; (1) present. **Remarks**: Underwood et al. (2017) have considered the presence of sexual heterodonty only in some pelagic stingrays (*Aetobatus* and *Mobula*) amongst living myliobatiforms. However, Herman et al. (1998, 1999, 2000) have suggested that this kind of sexual dimorphism is also present in *Urolophus, Taeniura, Neotrygon**, *Pteroplatytrygon, Dasyatis* and *Himantura*. Carvalho et al. (2004) recognized sexual heterodonty also in *Asterotrygon*. The character is coded as unknown for *Tethytrygon*.

90. (63 of UND) Medial symphyseal processes of the Meckel's cartilage: (0) absent; (1) present.

91. (68 of UND) Lateral processes of the palatoquadrate extending far anteriorly: (0) absent; (1) present.

92. (69 of UND) Anterior processes of the Meckel's cartilage: (0) absent; (1) present; (2) extending anterior past jaw joint.

93. (71 of UND) Lateral oral diastema alt: (0) diastema width greater than occlusal width; (1) occlusal width greater than diastema width.

94. (72 of UND) Upper jaw profile: (0) oval in cross-section (most batoids); (1) flat top, convex occlusal surface (myliobatids); (2) strongly flattened (mobulids).

95. (73 of UND) Upper jaw mineralization: (0) all surfaces mineralized; (1) lingual face partly unmineralized (mobulids).

96. (74 of UND) Lower jaw profile: (0) oval in cross-section; (1) strongly linguolabially expanded.

97. (75 and 76 of UND, modified): Upper and lower jaw trabeculae: (0) absent; (1) weakly developed; (2) strongly developed.

The characters 98 to 102 are the same reported in Marramà et al. (2018a), whereas ch. 103 is newly added.

98. Mesiodistally enlarged teeth up to one single tooth row: (0) absent; (1) present. **Remarks:** this character is described by Herman et al. (2000) who consider it a derived state for *Aetobatus, Aetomylaeus, Myliobatis, Pastinachus, Rhinoptera, Manta* and *Mobula*. This condition was not observed in *Tethytrygon* or described for other taxa, including *Neotrygon** (state 0).
99. Second transverse keel: (0) absent; (1) present. **Remarks**: A second transverse keel is present on teeth of *Himantura*, *Trygonoptera*, *Urobatis* and *Urolophus* according to Herman et al. (2000), and *Protohimantura* according to Marramà et al. (2018a). A second transverse is not present in the other fossil stingrays, including *Tethytrygon*.
100. Calcification pattern of radials: (0) crustal; (1) catenated. **Remarks**: Schaefer & Summers (2005) have shown that calcification of radials in batoids is strictly related to their swimming mode. Stiffer radials completely covered by mineralized tissue (“crustal calcification”) are typical of batoids with axial-undulatory or oscillatory swimming mode, including *Rhinobatos*, *Plesiobatis*, *Gymnura* and all pelagic stingrays. On the contrary, less stiffer radials calcified in chain-like patterns (“catenated calcification”) are typical of batoids with undulatory swimming mode, including *Raja*, and all myliobatiforms (except pelagic stingrays and *Plesiobatis*). All fossil stingrays in the matrix and are coded as (1) since they clearly show catenated calcification (see also Carvalho et al., 2004; Marramà et al., 2018a).
101. Body disc shape: (0) rhombus, quadrangular or oval, with pectoral fins not greatly expanded; (1) wing like, with pectoral fins greatly expanded. **Remarks**: this character is modified from Lim et al. (2015). Most of batoids have the general condition of a rhomboidal, quadrangular or oval shape of the disc, and pectoral fins are not greatly expanded (0), including outgroups and most of the myliobatiforms. *Gymnura* and all pelagic stingrays have the derived wing-like condition, with pectoral fins greatly expanded (Lim et al., 2015; but see also Last et al., 2016a). All fossil stingrays are coded as (0) since their disc clearly shows the basalmost condition.
102. Mid-dorsal surface of disc covered by heart-shaped denticles arranged in an antero-posteriorly directed patch having sharply defined outlines: (0) absent; (1) present. **Remarks:** this condition has been recognized as diagnostic for urogymnines, and distinguishes them from

all other members of the family Dasyatidae (Last et al., 2016a, b). In our matrix, this character is present in only *Himantura* and *Protohimantura* supporting their sister group relationship in the phylogenetic analysis.

103. File of enlarged 'caniniform' teeth in the upper jaw: (0) absent; (1) present. **Remarks**: amongst living stingrays, a single file of enlarged 'caniniform' teeth in the upper jaw has been recognized as a diagnostic character of the neotrygonines *Neotrygon* and *Taeniura* (Last et al. 2016a, b). This character is also present in *Tethytrygon*, and supports its polytomous relationship with the two living genera in our phylogeny.

DATA MATRIX

Taxon	1	2	3	4	5	6	7	8	9	10	11	12	13	14	15	16	17	18	19	20	21	22	23	24	25	26	27	28	29	30	31	32	33	34	35	36	37	38	39	40
Rhinobatos	0	0	0	0	0	0	0	0	0	0	0	0	0	0	0	0	0	0	0	0	0	0	0	0	0	0	0	0	0	0	0	0	0	0	0	0	0	0	0	0
Raja	0	0	0	0	0	0	0	0	0	0	0	0	0	0	0	0	0	0	0	0	0	0	0	0	0	0	0	0	0	0	0	0	0	0	0	0	0	0	0	0
Aetobatus	0	2	0	1	0	0	1	0	1	1	1	1	1	0	1	1	1	1	3	1	2	0	1	1	1	?	2	1	?	0	1	0	1	2	1	0	1	1	0	0
Aetomyleus	0	2	0	1	0	0	1	0	0	1	1	1	1	0	1	?	1	1	3	1	2	0	1	0	1	0	1	1	0	0	0	0	1	2	1	0	1	1	0	0
Asterotrygon	?	?	?	?	0	0	?	?	0	0	0	?	1	1	0	0	0	0	1	?	?	1	?	?	0	0	0	0	0	0	0	0	0	1	?	?	?	?	?	?
Dasyatis	0	1	[01]	1	0	0	0	0	0	0	0	1	1	0	0	0	0	0	2	1	1	1	0	0	1	0	[01]	0	0	0	0	1	1	1	0	0	0	0	0	0
Gymnura	0	1	0	1	0	0	0	0	0	1	0	1	0	0	0	0	0	0	1	1	1	1	0	0	0	0	1	1	1	0	1	1	0	2	0	0	0	0	0	0
Heliobatis	?	?	?	?	0	0	?	?	0	0	0	?	1	?	0	0	0	0	?	?	?	?	?	?	0	0	0	0	0	0	0	1	0	1	?	?	?	?	?	?
Heliotrygon	0	0	2	1	0	0	0	0	0	0	0	1	1	0	0	0	0	0	2	1	1	1	0	0	0	0	0	0	0	1	0	1	1	2	0	0	0	0	1	1
Hexatrygon	0	0	?	?	0	0	0	0	0	0	0	0	0	0	0	0	0	0	1	?	0	1	0	0	0	0	0	0	0	0	0	1	0	0	0	?	0	?	0	0
Himantura	0	1	[01]	1	0	0	0	0	0	0	0	1	1	0	0	0	0	0	2	1	1	1	0	0	1	0	0	0	0	0	0	1	1	2	0	0	0	0	0	0
Manta	?	?	?	?	?	?	?	?	?	?	?	?	?	?	?	?	?	?	?	?	?	?	?	?	?	?	?	?	?	?	?	?	?	?	?	?	?	?	?	?
Mobula	0	2	0	1	1	1	1	0	1	1	1	1	0	0	1	1	0	1	3	1	2	0	1	1	1	?	2	0	?	0	1	0	1	2	1	0	1	1	0	0
Myliobatis	0	2	0	1	0	0	1	0	0	1	1	1	1	0	1	?	1	1	3	1	2	0	1	0	1	0	1	1	0	0	0	0	1	2	1	0	1	1	0	0
Neotrygon	0	0	0	1	0	0	0	0	0	0	0	1	1	0	0	0	0	0	2	1	1	1	0	0	1	0	0	0	0	0	0	1	1	1	0	1	0	0	0	0
Paratrygon	0	0	2	1	0	0	0	0	0	0	0	1	1	0	0	0	0	0	2	1	1	1	0	0	0	0	0	0	0	1	0	1	1	2	0	0	0	0	1	1
Pastinachus	0	1	0	1	0	0	0	0	0	0	0	1	1	0	0	0	0	0	2	1	1	1	0	0	1	0	?	0	0	0	0	1	1	1	0	0	0	0	0	0
Plesiobatis	0	0	0	1	0	0	0	0	1	0	0	1	1	0	0	0	0	0	1	1	1	0	0	2	0	0	0	0	0	0	0	1	0	0	0	0	0	0	0	0
Plesiotrygon	0	0	1	1	0	0	0	0	0	0	0	1	1	1	0	0	0	0	2	1	1	1	0	1	0	1	0	0	0	1	0	1	1	1	0	2	0	0	1	1
Potamotrygon	0	0	1	1	0	0	0	0	0	0	0	1	1	1	0	0	0	0	2	1	1	1	0	1	0	1	0	0	0	1	0	1	1	1	0	2	0	0	1	1
Protohimantura	?	?	?	?	0	0	?	?	?	0	?	?	1	0	0	0	0	0	?	1	1	1	?	?	?	0	0	0	0	?	?	?	?	?	?	?	?	?	?	?
Pteroplatytrygon	0	1	0	1	0	0	0	0	1	0	0	1	1	0	0	0	0	0	2	1	1	1	0	0	1	0	0	0	0	0	0	1	1	1	0	0	0	0	0	0
Rhinoptera	0	2	0	1	1	1	1	0	1	1	1	1	1	0	1	1	1	1	3	1	2	0	1	?	1	?	2	0	?	0	1	0	1	2	1	0	1	1	0	0
Styracura	0	0	0	1	0	0	0	0	0	0	0	1	1	1	0	0	0	0	2	1	1	1	0	0	1	0	0	0	0	0	0	1	1	2	0	2	0	0	0	0
Taeniura	0	0	0	1	0	0	0	0	0	0	0	1	1	0	0	0	0	0	2	1	1	1	0	0	1	0	0	0	0	0	0	1	1	1	0	1	0	0	0	0
Tethytrygon	?	?	?	?	0	0	?	?	?	0	?	?	1	0	0	0	0	0	2	1	1	1	?	?	?	0	0	0	0	0	0	1	1	1	?	?	?	?	?	?
Trygonoptera	0	0	0	1	0	0	0	1	0	0	0	1	1	0	0	0	0	0	1	1	1	1	0	0	1	0	0	0	2	0	0	0	0	0	0	?	0	0	0	0
Urobatis	1	0	0	1	0	0	0	0	0	0	0	1	1	0	0	0	0	0	2	1	1	1	0	[01]	1	0	0	0	0	0	0	1	0	0	0	0	0	0	0	0
Urolophus	0	0	0	1	0	0	0	1	1	0	0	1	1	0	1	0	0	0	1	1	1	1	0	0	0	0	0	0	2	0	0	[01]	0	0	0	0	0	0	0	0
Urotrygon	1	0	0	1	0	0	0	0	0	0	0	1	1	0	0	0	0	0	3	1	1	1	0	0	1	0	0	0	0	0	0	1	0	0	0	0	0	0	0	0

Taxon	41	42	43	44	45	46	47	48	49	50	51	52	53	54	55	56	57	58	59	60	61	62	63	64	65	66	67	68	69	70	71	72	73	74	75	76	77	78	79	80
Rhinobatos	0	0	0	0	0	0	0	0	0	?	?	?	0	0	?	0	0	0	0	0	0	0	0	0	?	0	0	0	0	2	0	0	0	0	0	0	0	0	0	0
Raja	0	0	0	0	0	0	0	0	0	?	?	?	0	0	?	0	0	0	0	0	0	0	0	0	?	0	0	1	0	0	0	0	0	0	0	0	1	0	0	1
Aetobatus	0	2	1	1	1	2	1	?	?	2	1	0	2	1	2	1	1	0	0	1	1	2	0	2	0	1	1	2	1	3	2	1	1	1	1	3	2	1	1	1
Aetomyleus	0	1	1	?	?	1	0	1	0	0	0	?	1	1	1	?	1	?	?	1	1	1	0	?	?	?	1	[12]	0	?	2	?	1	?	?	?	2	1	1	1
Asterotrygon	?	0	?	0	0	0	0	0	0	?	?	?	0	0	?	0	0	0	0	0	0	0	0	0	?	?	1	1	0	?	?	?	1	1	1	0	2	1	1	1
Dasyatis	0	0	1	0	0	0	0	0	0	?	?	?	0	0	?	0	[12]	0	0	0	0	0	1	0	?	1	1	[12]	0	3	1	[12]	1	1	1	1	2	1	1	1
Gymnura	0	0	1	0	0	0	0	0	0	?	?	?	0	0	?	0	0	0	0	0	0	0	0	0	?	1	1	[12]	1	1	0	1	1	1	1	0	2	1	1	1
Heliobatis	?	0	?	0	0	0	0	0	0	?	?	?	0	0	?	0	0	0	0	0	0	0	0	0	?	?	1	1	0	?	?	?	1	1	1	0	2	1	1	1
Heliotrygon	0	0	1	?	?	0	0	0	0	?	?	?	?	?	?	?	?	?	?	?	?	?	?	?	?	?	?	?	?	?	?	?	?	?	?	?	?	?	?	?
Hexatrygon	0	0	0	0	0	0	0	0	0	?	?	?	0	0	?	0	0	0	0	0	0	0	0	0	?	1	1	2	1	1	0	?	1	1	1	0	2	1	1	1

Himantura	0	0	1	0	0	0	0	0	0	?	?	?	0	0	?	0	0	0	0	0	0	0	0	0	?	1	1	1	0	1	0	2	1	1	1	1	2	1	1	1
Manta	?	?	?	0	1	1	0	0	0	0	?	1	0	2	?	1	2	0	0	1	1	?	0	0	?	?	1	?	1	?	1	?	1	?	?	?	2	1	1	1
Mobula	0	3	1	0	1	1	0	1	1	0	?	1	0	1	?	1	[01]	0	0	1	1	?	0	0	?	1	1	[12]	1	?	0	1	1	1	1	3	2	1	1	1
Myliobatis	0	1	1	1	1	1	0	1	0	0	0	1	0	1	1	0	1	0	0	1	1	1	0	0	0	1	1	2	1	3	2	1	1	1	1	3	2	1	1	1
Neotrygon	0	0	1	0	0	0	0	0	0	?	?	?	0	0	?	0	0	0	0	0	0	0	0	0	?	0	1	[12]	[01]	1	0	1	1	1	0	1	2	1	1	1
Paratrygon	0	0	1	0	0	0	0	0	0	?	?	?	0	0	?	0	0	0	0	0	0	0	0	0	?	1	1	1	0	?	0	?	1	1	1	1	2	1	1	1
Pastinachus	0	0	1	?	?	0	0	0	0	?	0	?	0	0	0	0	[12]	?	?	0	0	0	0	?	?	?	1	1	1	?	2	?	1	?	?	?	2	1	1	1
Plesiobatis	0	0	1	0	0	0	0	0	0	?	?	?	0	0	?	0	0	0	0	0	0	0	0	0	?	1	1	1	1	?	0	1	1	1	1	0	2	1	1	1
Plesiotrygon	0	0	1	0	0	0	0	0	0	?	?	?	0	0	?	0	0	0	0	0	0	0	0	0	?	1	1	1	0	?	1	?	1	1	1	1	2	1	1	1
Potamotrygon	0	0	1	0	0	0	0	0	0	?	?	?	0	0	?	0	0	0	0	0	0	0	0	0	?	1	1	1	0	1	1	3	1	1	1	1	2	1	1	1
Protohimantura	?	?	?	0	0	0	0	0	0	?	?	?	0	0	?	0	0	0	0	0	0	0	0	0	?	?	?	1	?	?	?	?	1	1	?	?	2	?	1	1
Pteroplatytrygon	0	0	1	0	0	0	0	0	0	?	?	?	0	0	?	0	0	0	0	0	0	0	0	0	?	1	1	1	0	?	1	1	1	1	1	1	2	1	1	1
Rhinoptera	0	3	1	1	1	1	0	1	1	1	1	2	0	1	0	0	1	0	0	1	1	2	1	1	0	1	1	2	1	3	2	1	1	1	1	3	2	1	1	1
Styracura	0	0	1	0	0	0	0	0	0	?	?	?	0	0	?	0	0	0	0	0	0	0	0	0	?	1	1	1	0	?	?	1	1	1	1	1	2	1	1	1
Taeniura	0	0	1	0	0	0	0	0	0	?	?	?	0	0	?	0	0	0	0	0	0	0	0	0	?	1	1	[12]	0	3	1	1	1	1	1	1	2	1	1	1
Tethytrygon	?	0	?	0	0	0	0	0	0	?	?	?	0	0	?	0	0	0	0	0	0	0	0	0	?	?	1	2	1	?	?	?	1	1	?	1	2	1	1	1
Trygonoptera	0	0	1	0	0	0	0	0	0	?	?	?	0	0	?	0	0	0	0	0	0	0	0	0	?	1	1	2	1	?	0	?	1	1	1	?	2	1	1	1
Urobatis	1	0	1	0	0	0	0	0	0	?	?	?	0	0	?	0	0	0	0	0	0	0	0	0	?	1	1	[12]	0	?	0	1	1	1	1	1	2	1	1	1
Urolophus	0	0	1	0	0	0	0	0	0	?	?	?	0	0	?	0	0	0	0	0	0	0	0	0	?	1	1	2	1	1	0	1	1	1	1	0	2	1	1	1
Urotrygon	1	0	1	0	0	0	0	0	0	?	?	?	0	0	?	0	0	0	0	0	0	0	0	0	?	1	1	[12]	0	1	0	1	1	1	1	2	2	1	1	1

**Taxon**	**81**	**82**	**83**	**84**	**85**	**86**	**87**	**88**	**89**	**90**	**91**	**92**	**93**	**94**	**95**	**96**	**97**	**98**	**99**	**100**	**101**	**102**	**103**
Rhinobatos	1	0	0	0	0	0	0	0	0	0	0	0	0	0	0	0	0	0	0	0	0	0	0
Raja	0	1	0	0	3	1	1	0	0	0	0	0	0	0	0	0	0	0	0	1	0	0	0
Aetobatus	3	0	1	0	2	2	0	?	1	0	1	0	0	0	0	1	1	1	0	0	1	0	0
Aetomyleus	?	?	?	?	?	?	?	?	0	0	0	2	0	1	0	1	1	1	0	0	1	0	0
Asterotrygon	?	?	?	?	?	?	?	?	1	?	?	?	?	?	?	?	?	0	0	1	0	0	0
Dasyatis	3	1	0	1	1	2	1	1	1	1	0	2	0	0	0	0	0	0	0	1	0	0	0
Gymnura	3	0	1	0	2	2	0	?	0	0	0	0	1	0	0	0	0	0	0	0	1	0	0
Heliobatis	?	?	?	?	?	?	?	?	?	?	?	?	?	?	?	?	?	0	0	1	0	0	0
Heliotrygon	?	?	?	?	?	?	?	?	0	0	0	0	0	0	0	0	0	?	?	?	0	0	0
Hexatrygon	2	?	?	?	?	?	?	?	0	0	0	0	0	0	0	0	0	0	0	1	0	0	0
Himantura	3	0	1	0	1	2	1	2	1	1	0	2	0	0	0	0	0	0	1	1	0	1	0
Manta	?	?	?	?	?	?	?	?	0	0	0	0	1	2	1	1	1	1	0	?	1	0	0
Mobula	?	1	?	?	?	?	?	?	1	0	0	0	1	2	1	1	2	1	0	0	1	0	0
Myliobatis	3	0	1	0	2	2	0	0	0	0	1	2	0	1	0	1	1	1	0	0	1	0	0
Neotrygon	2	0	0	1	1	2	1	2	1	0	0	1	0	0	0	0	0	0	0	?	0	0	1
Paratrygon	?	0	?	?	?	?	?	2	0	0	0	0	1	0	0	0	0	0	0	1	0	0	0
Pastinachus	?	?	?	?	?	?	?	?	0	0	0	0	0	0	0	1	0	1	0	1	0	0	0
Plesiobatis	1	0	1	0	?	2	0	1	0	0	0	0	0	0	0	0	0	0	0	0	0	0	0
Plesiotrygon	?	0	?	?	?	?	?	2	0	0	0	0	0	0	0	0	0	0	0	1	0	0	0
Potamotrygon	2	0	1	0	1	2	0	2	0	0	0	0	0	0	0	0	0	0	0	1	0	0	0
Protohimantura	3	?	?	?	?	?	?	?	?	?	?	?	0	?	0	?	?	0	1	1	0	1	0
Pteroplatytrygon	3	1	0	1	1	2	1	1	1	0	0	0	0	0	0	0	0	0	0	1	0	0	0
Rhinoptera	?	0	1	0	2	2	0	3	?	?	0	0	?	1	0	1	1	1	0	0	1	0	0

Styracura	2	?	?	?	?	?	?	?	0	0	0	?	0	0	0	0	0	0	0	1	0	0	0
Taeniura	2	0	0	1	1	2	1	2	1	0	0	1	0	0	0	0	0	0	0	1	0	0	1
Tethytrygon	2	?	?	1	?	?	1	?	1	?	?	1	0	?	0	?	?	0	0	1	0	0	1
Trygonoptera	?	0	?	?	?	?	?	?	0	0	0	1	0	0	0	0	0	0	1	1	0	0	0
Urobatis	1	0	1	0	2	2	0	1	0	0	0	0	0	0	0	0	0	0	1	1	0	0	0
Urolophus	1	0	1	0	2	2	0	1	1	0	0	0	0	0	0	0	0	0	1	1	0	0	0
Urotrygon	1	0	1	0	2	2	0	1	0	0	0	0	0	0	0	0	0	0	0	1	0	0	0

MORPHOMETRIC DATA

Morphometric data for 11 of the 13 examined specimens of *Tethytrygon muricatus* (Volta, 1796) from the Eocene Pesciara site, Bolca Lagerstätte. Other two specimens were poorly preserved to detect any reliable measurement.

	MNHN Bol.564		MGP-PD 150Z/151Z		CM 4521		CMC2		MCSNV IG.23194		MCSNV IG.186653	
**Measurements**	**mm**	**% DW**	**mm**	**% DW**	**mm**	**% DW**	**mm**	**% DW**	**mm**	**% DW**	**mm**	**% DW**
Total length	?	?	?	?	?	?	?	?	?	?	?	?
Disc length	243.4	86.3	128.4	89.6	193.5	94.9	128.8	88.2	574.5	95.3	293.6	104.2
Disc width	282	100.0	143.3	100.0	203.9	100.0	146	100.0	603.0	100.0	281.9	100.0
Tail length	?	?	?	?	?	?	?	?	?	?	?	?
Preoral length	37.5	13.3	22.2	15.5	26.2	12.8	22.1	15.1	89.7	14.9	47	16.7
Mouth-scapulocoracoid distance	83	29.4	39.1	27.3	65.3	32.0	42.9	29.4	209.0	34.7	81.6	28.9
Scapulocoracoid width	51.2	18.2	30	20.9	49.1	24.1	30.7	21.0	118.8	19.7	61.3	21.7
Pelvic girdle width (width across pelvic-fin base)	61.3	21.7	30.5	21.3	43.1	21.1	30.6	21.0	?	?	66.9	23.7
Sting length	71.3	25.3	?	?	66.8	32.8	35.6	24.4	?	?	94.2	33.4
Pelvics-tip of tail length	?	?	?	?	?	?	?	?	?	?	?	?
Clasper length	?	?	?	?	?	?	?	?	114.8	19.0	?	?
Neurocranial length	60.9	21.6	33.6	23.4	58.8	28.8	34.5	23.6	150.8	25.0	66.9	23.7
Neurocranial width	41.1	14.6	21.1	14.7	40.6	19.9	24	16.4	?	?	41.4	14.7
Pre-sting length	484.5	171.8	?	?	?	?	220.9	151.3	?	?	490.2	173.9
Distance from tip of disc to max width disc	99.9	35.4	53.3	37.2	74.6	36.6	62.9	43.1	246.2	40.8	147.3	52.3
Prepelvic distance	199.9	70.9	109.1	76.1	157.1	77.0	102.1	69.9	?	?	218.6	77.5
Prescapular distance (head length)	122.4	43.4	61.4	42.8	86.0	42.2	66.7	45.7	302.2	50.1	120.8	42.9
Eye diameter	?	?	3.6	2.5	?	?	6.2	4.2	?	?	8.3	2.9
Interorbital width	?	?	23	16.1	?	?	12.6	8.6	?	?	49.2	17.5
Pelvic fin length	78.2	27.7	33.1	23.1	53.3	26.1	43.1	29.5	?	?	92.6	32.8
Snout to pectoral fin insertion	225.6	80.0	112.9	78.8	170.9	83.8	110.2	75.5	?	?	241.6	85.7
Orbit to pectoral fin insertion	?	?	93	64.9	128.9	63.2	82	56.2	?	?	184.5	65.4
Snout (preorbital) length	?	?	21.2	14.8	35.6	17.5	26.8	18.4	?	?	52.4	18.6
Pectoral-fin insertion to sting	247.9	87.9	?	?	?	?	103.2	70.7	?	?	249.7	88.6
Propterygial radials	50	?	51	?	?	?	51	?	51	?	53	?
Mesopterygial radials	?	?	19	?	?	?	17	?	16	?	19	?
Metapterygial radials	?	?	43	?	?	?	40	?	42	?	45	?
Total pectoral radials	?	?	113	?	?	?	108	?	109	?	117	?
Pelvic radials	25	?	27	?	26	?	25	?	25	?	25	?
Vertebrae from scapulocoracoid to pelvic girdle	25	?	24	?	25	?	23	?	?	?	24	?
Vertebrae from pelvic girdle to sting	?	?	?	?	?	?	102	?	?	?	109	?

Vertabrae posterior to sting	?	?	?	?	?	?	54	?	?	?	46	?
Total vertebrae	?	?	?	?	?	?	179	?	?	?	179	?
Sting serrations per side	?	?	?	?	?	?	24	?	?	?	30	?

	MCSNV T.1020/1		MCSNV VII.B.92/3		MCZ 13183		MGGC 7456		MGP-PD 159/160	
**Measurements**	**mm**	**% DW**	**mm**	**% DW**	**mm**	**% DW**	**mm**	**% DW**	**mm**	**% DW**
Total length	759.2	263.0	627.2	249.5	?	?	728.7	252.4	?	?
Disc length	279.9	97.0	236.8	94.2	110.1	90.2	250.7	86.8	252.2	89.1
Disc width	288.7	100.0	251.4	100.0	122.1	100.0	288.7	100.0	283	100.0
Tail length	533.3	184.7	428.3	170.4	?	?	518.2	179.5	?	?
Preoral length	51.4	17.8	?	?	18.2	14.9	?	?	38	13.4
Mouth-scapulocoracoid distance	78	27.0	?	?	37.7	30.9	?	?	87.3	30.8
Scapulocoracoid width	59	20.4	57.4	22.8	24.2	19.8	54.9	19.0	68.6	24.2
Pelvic girdle width (width across pelvic-fin base)	63.6	22.0	56.6	22.5	34.6	28.3	64.3	22.3	66.1	23.4
Sting length	82.5	28.6	?	?	?	?	89.6	31.0	91.8	32.4
Pelvics-tip of tail length	452.5	156.7	356.4	141.8	?	?	428.5	148.4	?	?
Clasper length	?	?	?	?	?	?	?	?	?	?
Neurocranial length	71.5	24.8	57.3	22.8	?	?	60	20.8	69.9	24.7
Neurocranial width	?	?	46.1	18.3	?	?	?	?	42.1	14.9
Pre-sting length	472.5	163.7	361	143.6	213	174.4	469.2	162.5	443.1	156.6
Distance from tip of disc to max width disc	110.6	38.3	112	44.6	?	?	113.5	39.3	95	33.6
Prepelvic distance	224.4	77.7	199.9	79.5	97.3	79.7	211.6	73.3	203.6	71.9
Prescapular distance (head length)	126.8	43.9	132.2	52.6	?	?	144.1	49.9	126	44.5
Eye diameter	13.5	4.7	10.5	4.2	?	?	?	?	?	?
Interorbital width	36.7	12.7	45.1	17.9	?	?	?	?	?	?
Pelvic fin length	85.6	29.7	76.2	30.3	?	?	70.5	24.4	69.6	24.6
Snout to pectoral fin insertion	248	85.9	221.5	88.1	?	?	215.1	74.5	239.2	84.5
Orbit to pectoral fin insertion	197.1	68.3	175.9	70.0	?	?		?		?
Snout (preorbital) length	43.9	15.2	42	16.7	?	?		?		?
Pectoral-fin insertion to sting	220.8	76.5	?	?	?	?	216.6	75.0	214.3	75.7
Propterygial radials	50	?	49	?	?	?	50	?	50	?
Mesopterygial radials	20	?	16	?	?	?	17	?	18	?
Metapterygial radials	44	?	45	?	?	?	?	?	45	?
Total pectoral radials	114	?	110	?	?	?	?	?	113	?
Pelvic radials	25	?	24	?	?	?	25	?	25	?
Vertebrae from scapulocoracoid to pelvic girdle	26	?	25	?	?	?	23	?	24	?

Vertebrae from pelvic girdle to sting	105	?	?	?	?	?	?	?	100	?
Vertabrae posterior to sting	45	?	?	?	?	?	?	?	?	?
Total vertebrae	176	?	175	?	?	?	?	?	?	?
Sting serrations per side	33	?	43	?	?	?	45	?	39	?

SUPPLEMENTARY REFERENCES

Adnet, S., Mouana, M., Charruault, A.-L., Essid, E. M., Khayati Ammar, H., Marzougui, W., Merzeraud, G., Tabuce, R., Vianey-Liaud, M., & Marivaux, L. (2018). Teeth, fossil record and evolutionary history of the cowtail stingray Pastinachus Rüppell, 1829. *Historical Biology*. https://doi.org/10.1080/08912963.2018.1431779.

Agassiz, L. (1833–1844). *Recherches sur les Poissons fossiles*. Neuchâtel, Petitpierre et Prince.

Agassiz, L. (1835). *Revue critique des Poissons fossiles figurés dans l'Ittiolitologia Veronese*. Neuchâtel, Petitpierre et Prince.

Aschliman, N. C, Claeson, K. M., & McEachran, J. D. (2012a). Phylogeny of Batoidea. In J. C. Carrier, J. A. Musick, & M. R. Heithaus (Eds.), *Biology of sharks and their relatives, 2nd edn*. (pp. 57–96), Boca Raton: CRC Press.

Blainville, H. D. de. (1818). Sur les ichthyolites ou les poissons fossiles. *Nouveau Dictionnaire d'Histoire Naturelle*, 27, 310–391.

Blot, J. (1980). La faune ichthyologique des gisements du Monte Bolca (Province de Verone, Italie). Catalogue systématique présentant l'état actuel des 160 recherches concernant cette faune. *Bulletin du Muséum national d'histoire naturelle Paris*, 2, 339–396.

Bronn, H. G. (1831). *Italiens Tertiär-Gebilde und deren organische Einschlüsse*. Heidelberg, Neue akademische Buschandlung von Karl Groos.

Cappetta, H. (1980). Les selaciens du Cretace superieur du Liban. II: batoides. *Palaeontographica, Abteilung A*, 168, 149–229.

Cappetta, H. (1987). *Handbook of paleoichthyology, 3B – Chondrichthyes II – Mesozoic and Cenozoic Elasmobranchii*. Stuttgart, Gustav Fischer Verlag.

Cappetta, H. (2012). *Handbook of paleoichthyology – Chondrichthyes – Mesozoic and Cenozoic elasmobranchii: teeth*. Munich, Verlag Dr. Friedrich Pfeil.

Carnevale, G., Bannikov, A. F., Marramà, G., Tyler, J. C., & Zorzin, R. (2014). The Pesciara-Monte Postale Fossil-Lagerstätte: 2. Fishes and other vertebrates. In C. A. Papazzoni, L. Giusberti, G. Carnevale, G. Roghi, D. Bassi, & R. Zorzin (Eds.),*The Bolca Fossil-Lagerstätte: A window into the Eocene World* (pp. 37–63), Modena: Società Paleontologica Italiana.

Carvalho, M. R., Loboda, T. S, & Silva, J. P. (2016). A new subfamily, Styracurinae, and new genus, *Styracura*, for *Himantura schmardae* (Werner, 1904) and *Himantura pacifica* (Beebe & Tee-Van, 1941) (Chondrichthyes: Myliobatiformes). *Zootaxa*, 4175, 201–221.

Carvalho, M. R., Maisey, J. C., & Grande, L. (2004). Freshwater stingrays of the Green River Formation of Wyoming (Early Eocene), with the description of a new genus and species and

an analysis of its phylogenetic relationships (Chondrichthyes: Myliobatiformes). *Bulletin of the American Museum of Natural History*, 284, 1–136.

Catullo, T. A. (1827). *Saggio di zoologia fossile*. Padova, Dalla Tipografia del Seminario.

Claeson, K. M., O'Leary, M. A., Roberts, E. M., Sissoko, F., Bouaré, M., Tapanila, L., … Gottfried, M. D. (2010). First Mesozoic record of the stingray *Myliobatis wurnoensis* from Mali and a phylogenetic analysis of Myliobatidae incorporating dental characters. *Acta Palaeontologica Polonica*, 55, 655–674.

Compagno, L. J. V. (1973). Interrelationships of living elasmobranchs. In P. H. Greenwood, R. S. Miles, & C. Patterson (Eds.) *Interrelationships of fishes* (pp. 15–61). New York: Academic Press.

Compagno, L. J. V. (1977). Phyletic relationships of living sharks and rays. *American Zoologist*, 17, 303–322.

D'Erasmo, G. (1922). Catalogo dei pesci fossili delle Tre Venezie. *Memorie dell'Istituto di Geologia della Regia Università di Padova*, 6, 1–181.

Dal Sasso, C., & Maganuco, S. (2011). *Scipionyx samniticus* (Theropoda: Compsognathidae) from the Lower Cretaceous of Italy Osteology, ontogenetic assessment, phylogeny, soft tissue anatomy, taphonomy and palaeobiology. *Memorie della Società Italiana di Scienze Naturali e del Museo Civico di Storia Naturale di Milano*, 37, 1–281.

Dean, M. N., & Summers, A. P. (2006). Mineralized cartilage in the skeleton of chondrichthyan fishes. *Zoology*, 109, 164–168.

Eastman, C. R. (1904). Description of Bolca fishes. *Bulletin of the Museum of Comparative Zoology*, 46, 1–36.

Eastman, C. R. (1905a). Les types de Poissons fossiles du Monte Bolca au Muséum d'Histoire Naturelle de Paris. *Mémoires de la Société géologique de France*, 34, 1–33.

Eastman, C. R. (1905b). Catalog of fossil fishes in the Carnegie Museum. Part I. *Fishes from the Upper Eocene of Monte Bolca*, 4, 349–415.

Eastman, C. R. (1911). Catalog of the fossil fishes in the Carnegie Museum. Part II. Supplement to the catalog of fishes from the Upper Eocene of Monte Bolca. *Memoirs of the Carnegie Museum*, 6, 315–348.

Frickhinger, K. A. (1991). *Fossilien Atlas: Fische*. Mergus, Melle.

Giebel, C. G. (1848). *The fish of the previous world, with constant consideration of the living fish. First volume: vertebrate animals. Third Division: Pisces: I-XII*. Leipzig, Brockhaus.

Herman, J., Hovestadt-Euler, M., Hovestadt, D. C., & Stehmann, M. (1998). Contributions to the study of the comparative morphology of teeth and other relevant ichthyodorulites in living

supra-specific taxa of Chondrichthyan fishes. Part B: Batomorphii 4a: Order Rajiformes - Suborder Myliobatoidei - Superfamily Dasyatoidea - Family Dasyatidae - Subfamily Dasyatinae - Genera: *Amphotistius, Dasyatis, Himantura, Pastinachus, Pteroplatytrygon, Taeniura, Urogymnus* and *Urolophoides* (incl. supraspecific taxa of uncertain status and validity), Superfamily Myliobatoidea - Family Gymnuridae - Genera: *Aetoplatea* and *Gymnura*, Superfamily Plesiobatoidea - Family Hexatrygonidae - Genus: *Hexatrygon*. *Bulletin de l'Institut Royal des Sciences Naturelles de Belgique, Biologie*, 68, 145–197.

Herman, J., Hovestadt-Euler, M., Hovestadt, D. C., & Stehmann, M. (1999). Contributions to the study of the comparative morphology of teeth and other relevant ichthyodorulites in living supra-specific taxa of Chondrichthyan fishes. Part B: Batomorphii 4b: Order Rajiformes - Suborder Myliobatoidei - Superfamily Dasyatoidea - Family Dasyatidae - Subfamily Dasyatinae - Genera: *Taeniura, Urogymnus, Urolophoides* - Subfamily Potamotrygoninae - Genera: *Disceus, Plesiotrygon,* and *Potamotrygon* (incl. supraspecific taxa of uncertain status and validity), Family Urolophidae - Genera: *Trygonoptera, Urolophus* and Urotrygon - Superfamily Myliobatidea - Family: Gymnuridae - Genus: Aetoplatea. Bul*letin de l'Institut Royal des Sciences Naturelles de Belgique, Biologie,* 69, 161–200.

Herman, J., Hovestadt-Euler, M., Hovestadt, D. C., & Stehmann, M. (2000). Contributions to the study of the comparative morphology of teeth and other relevant ichthyodorulites in living supra-specific taxa of Chondrichthyan fishes. Part B: Batomorphii 4c: Order Rajiformes - Suborder Myliobatoidei - Superfamily Dasyatoidea - Family Dasyatidae - Subfamily Dasyatinae - Genus: *Urobatis*, Subfamily Potamotrygoninae Genus: *Paratrygon*, Superfamily Plesiobatoidea - Family Plesiobatidae - Genus: *Plesiobatis*, Superfamily Myliobatoidea - Family Myliobatidae - Subfamily Myliobatinae - Genera: *Aetobatus, Aetomylaeus*, *Myliobatis* and *Pteromylaeus,* Subfamily Rhinopterinae - Genus: *Rhinoptera* and Subfamily Mobulinae - Genera: *Manta* and *Mobula*. Addendum 1 to 4a: erratum to Genus *Pteroplatytrygon. Bulletin de l'Institut Royal des Sciences Naturelles de Belgique, Biologie*, 70, 5–67.

Hueter, R. E., Mann, D. A., Maruska, K. P., Sisneros, J. A., & Demski, L.S. (2004). Sensory biology of elasmobranchs. In J. C. Carrier, J. A. Musick, & M. R. Heithaus (Eds.), *Biology of sharks and their relatives* (pp. 325–368). Boca Raton: CRC Press.

Huxley, T. H. (1880). On the application of the laws of evolution to the arrangement of the Vertebrata, and more particularly of the Mammalia. *Proceedings of the Zoological Society, London,* 43, 649–662.

Jaekel, O. (1894). *Die eocänen Selachier vom Monte Bolca: ein Beitrag zur Morphogenie der Wirbelthiere.* Berlin, J. Springer.

Jordan, D. S. (1888). Description of two new species of fishes from South America. *Proceedings of the Academy of Natural Sciences of Philadelphia*, 39, 387–388.

Last, P. R., Naylor, G. J. P., & Manjaji-Matsumoto, B. M. (2016b). A revised classification of the family Dasyatidae (Chondrichthyes: Myliobatiformes) based on new morphological and molecular insights. *Zootaxa*, 4139, 345–368.

Last, P. R., & White, W. T. (2008). Resurrection of the genus *Neotrygon* Castelnau (Myliobatoidei: Dasyatidae) with the description of *Neotrygon picta* sp. n., a new species from the Northern Australia. In P. R. Last, W. T. White, & J. J. Pogonoski (Eds.), *Descriptions of New Australian Chondrichthyans* (pp. 315–325), Hobart: CSIRO Marine and Atmospheric Research.

Last, P. R., White, W., Carvalho, M. R., Séret, B., Stehmann, M., & Naylor, G. J. P. (2016a). *Rays of the world*. Clayton North, CSIRO Publishing.

Last, P. R., White, W. T., & Naylor, G. J. P. (2016c). Three new stingrays (Myliobatiformes: Dasyatidae) from the Indo–West Pacific. *Zootaxa*, 4147, 377–402.

Leriche, M. (1906). Contribution a l'étude des poissons fossiles du Nord de la France et des régions voisines. *Mémoires de la Société Géologique du Nord*, 5, 1–430.

Lim, K. C., Lim, P. E., Chong, V. C., & Loh, K. H. (2015). Molecular and morphological analyses reveal phylogenetic relationships of stingrays focusing on the family Dasyatidae (Myliobatiformes). *PLoS ONE*, 10, e0120518.

Lovejoy, N. R. (1996). Systematics of myliobatoid elasmobranchs: with emphasis on the phylogeny and historical biogeography of Neotropical freshwater stingrays (Potamotrygonidae: Rajiformes). *Zoological Journal of the Linnean Society*, 117, 207–257.

Marramà, G., Bannikov, A. F., Tyler, J. C., Zorzin, R. & Carnevale, G. (2016c). Controlled excavations in the Pesciara and Monte Postale sites provide new insights about the paleoecology and taphonomy of the fish assemblages of the Eocene Bolca Konservat-Lagerstätte, Italy. *Palaeogeography, Palaeoclimatology, Palaeoecology*, 454, 228–245.

Marramà, G., & Carnevale, G. (2015a). The Eocene sardine *Bolcaichthys catopygopterus* (Woodward, 1901) from Monte Bolca, Italy: osteology, taxonomy, and paleobiology. *Journal of Vertebrate Paleontology*, 35, e1014490. https://doi.org/10.1080/02724634.2015.1014490.

Marramà, G., & Carnevale, G. (2015b). Eocene round herring from Monte Bolca, Italy. *Acta Palaeontologica Polonica*, 60, 701–710.

Marramà, G., & Carnevale, G. (2016). An Eocene anchovy from Monte Bolca, Italy: The earliest known record for the family Engraulidae. *Geological Magazine*, 153, 84–94.

Marramà, G., & Carnevale, G. (2018). *Eoalosa janvieri* gen. et sp. n., a new clupeid fish (Teleostei, Clupeiformes) from the Eocene of Monte Bolca, Italy. *Paläontologische Zeitschrift*, 92, 107–120. http://dx.doi.org/10.1007/s12542-017-0378-0.

Marramà, G., Carnevale, G., Engelbrecht, A., Claeson, K. M., Zorzin, R., Fornasiero, M., & Kriwet, J. (2018b). A synoptic review of the Eocene (Ypresian) cartilaginous fishes (Chondrichthyes: Holocephali, Elasmobranchii) of the Bolca Konservat-Lagerstätte, Italy. *Paläontologische Zeitschrift*, 92, 283–313. http://dx.doi.org/10.1007/s12542-017-0387-z

Marramà, G., Carnevale, G., & Kriwet, J. (2018c). New observations on the anatomy and paleobiology of the Eocene requiem shark *Eogaleus bolcensis* (Carcharhiniformes, Carcharhinidae) from Bolca Lagerstätte, Italy. *Comptes Rendus Palevol*, 17, 443–459. https://doi.org/10.1016/j.crpv.2018.04.005

Marramà, G., Klug, S., De Vos, J., & Kriwet, J. (2018a). Anatomy, relationships and palaeobiogeographic implications of the first Neogene holomorphic stingray (Myliobatiformes: Dasyatidae) from the early Miocene of Sulawesi, Indonesia, SE Asia. *Zoological Journal of the Linnean Society*. https://doi.org/10.1093/zoolinnean/zly020

Marramà, G., Schultz, O., & Kriwet J. (2018d). A new Miocene skate from Central Paratethys (Upper Austria): The first unambiguous skeletal record for the Rajiformes (Chondrichthyes: Batomorphii). *Journal of Systematic Palaeontology*. https://doi.org/10.1080/14772019.2018.1486336

Martin, T., Marugán-Lobón, J., Vullo, R., Martín-Abad, H., Luo, Z.-X., & Buscalion, A. D. (2015). A Cretaceous eutriconodont and integument evolution in early mammals. *Nature*, 526, 380–384. https://doi.org/10.1038/nature14905

McEachran, J. D., Dunn, K. A., & Miyake, T. (1996). Interrelationships of the batoid fishes (Chondrichthyes: Batoidea). In M. J. Stiassny, L. R. Parenti, & G. D. Johnson (Eds.), *Interrelationships of fishes* (pp. 63–82), London: Academic Press.

Miyake, T. (1988). The systematics of the stingray Genus *Urotrygon* with comments on the interrelationships within Urolophidae (Chondrichthyes: Myliobatiformes). Unpublished D. Phil. Thesis, College Station: Texas A&M University.

Miyake, T., & McEachran, J. D. (1991). The morphology and evolution of the ventral gill arch skeleton in batoid fishes (Chondrichthyes: Batoidea). *Zoological Journal of the Linnean Society*, 102, 75–100.

Miyake, T., McEachran, J. D., Walton, P. J., & Hall, B. K. (1992). Development and morphology of rostral cartilages in batoid fishes (Chondrichthyes: Batoidea), with comments on homology within vertebrates. *Biological Journal of the Linnean Society*, 46, 259–298.

Molin, R. (1861). De Rajidis tribus bolcanis. *Sitzungsberichte der Kaiserlichen Akademie der Wissenschaften (Mathematisch-naturwissenschaftliche Klasse)*, 42, 576–582.

Nishida, K. (1990). Phylogeny of the suborder Myliobatoidei. *Hokkaido University Fisheries Memoir*, 37, 1–108.

Papazzoni, C. A., Carnevale, G., Fornaciari, E., Giusberti, L., & Trevisani, E. (2014). The Pesciara-Monte Postale Fossil-Lagerstätte: 1. Biostratigraphy, sedimentology and depositional model. In C.A. Papazzoni, L. Giusberti, G. Carnevale, G. Roghi, D. Bassi, & R. Zorzin (Eds.), *The Bolca Fossil-Lagerstätte: A window into the Eocene World* (pp. 29–36), Modena: Società Paleontologica Italiana.

Papazzoni, C. A., & Trevisani, E. 2006. Facies analysis, palaeoenvironmental reconstruction, and biostratigraphy of the "Pesciara di Bolca" (Verona, northern Italy): An early Eocene Fossil-Lagerstätte. *Palaeogeography, Palaeoclimatolology, Palaeoecology*, 242, 21–35.

Pavan-Kumar, A., Kumar, R., Pitale, P., Shen, K.-N., & Borsa, P. (2018). *Neotrygon indica* sp. n., the Indian-Ocean blue spotted maskray (Myliobatoidei, Dasyatidae). *Comptes Rendus Biologies*, 341, 120–130. https://doi.org/10.1016/j.crvi.2018.01.004

Rosenberger, L. J. (2001). Phylogenetic relationships within the stingray genus *Dasyatis* (Chondrichthyes: Dasyatidae). *Copeia*, 2001, 615–627.

Schaefer, J. T., & Summers, A. P. (2005). Batoid wing skeletal structure: novel morphologies, mechanical implications, and phylogenetic patterns. *Journal of Morphology*, 264, 298–313.

Schwartz, F. J. (2005). Tail spine characteristics of stingrays (order Myliobatiformes) found in the northeast Atlantic, Mediterranean, and Black Seas. *Electronic Journal of Ichthyology*, 1, 1–9.

Schwartz, F. J. (2007). Tail spine characteristics of stingrays (Order Myliobatiformes) frequenting the FAO Fishing Area 61 (20°N 120°E - 50°N 150°E) of the northwest Pacific Ocean. *The Raffles Bulletin of Zoology*, 14, 121–130.

Schwartz, F. J. (2008). A survey of tail spine characteristics of stingrays frequenting African, Arabian to Chagos-Maldive Archipelago waters. Smithiana Bulletin, 8, 41–52.

Trevisani, E. (2015). Upper Cretaceous–Lower Eocene succession of the Monte Postale and its relationship with the 'Pesciara di Bolca' (Lessini Mountains, northern Italy): deposition of a fossil-fish lagerstätte. *Facies*, 61, 1–17.

Underwood, C. J., Johanson, Z., Welten, M., Metscher, B., Rasch, L. J., Fraser, G. J., & Smith, M. M. (2015). Development and evolution of dentition pattern and tooth order in the skates and rays (Batoidea; Chondrichthyes). *PLoS ONE*, 10, e0122553. https://doi.org/10.1371/journal.pone.0122553

Underwood, C. J., Kolmann, M. A., & Ward, D. J. (2017). Paleogene origin of planktivory in the Batoidea. *Journal of Vertebrate Paleontology*. https://doi.org/10.1080/02724634.2017.1293068

Underwood, C. J., Ward, D. J., King, C., Antar, S. M., Zalmout, I. S., & Gingerich, P. D. (2011). Shark and ray faunas in the middle and late Eocene of the Fayum Area, Egypt. *Proceedings of the Geologists' Association*, 122, 47–66.

Volta, G. S. (1796). *Ittiolitologia Veronese del Museo Bozziano ora annesso a quello del Conte Giovambattista Gazola e di altri gabinetti di fossili veronesi*. Verona, Stamperia Giuliari.

Vullo, R., Guinot, G., & Barbe, G. (2016). The first articulated specimen of the Cretaceous mackerel shark *Haimirichia amonensis* gen. n. (Haimirichiidae fam. n.) reveals a novel ecomorphological adaptation within the Lamniformes (Elasmobranchii). *Journal of Systematic Palaeontology*, 14, 1003–1024, http://dx.doi.org/10.1080/14772019.2015.1137983.

Woodward, A. S. (1889). *Catalogue of the fossil fishes in the British Museum. Part. I. Elasmobranchii.* London, British Museum (Natural History).

Zigman, S. (1991). Comparative biochemistry and biophysics of elasmo-branch lenses. *Journal of Experimental Zoology*, 256, 29–40.

Zigno, A. de. (1874a). Annotazioni paleontologiche. Pesci fossili nuovi del calcare eoceno dei monti Bolca e Postale. *Memorie del Reale Istituto Veneto di Scienze, Lettere ed Arti*, 18, 287–301.

Zigno, A. de. (1874b). *Catalogo Ragionato dei Pesci Fossili del Calcare Eoceno di M. Bolca e M. Postale*. Venice, Stabilimento Tipografia Grimaldo e C.
